# Spatial profiling of longitudinal glioblastoma reveals consistent changes in cellular architecture, post-treatment

**DOI:** 10.1093/neuonc/noaf190

**Published:** 2025-09-01

**Authors:** Shoaib Ajaib, Joshua Winter-Luke, Richard J Digby, Steven Pollock, Gemma Hemmings, Arief Gusnanto, Aruna Chakrabarty, Azzam Ismail, Erica Wilson, Bethany Hunter, Andrew Filby, David McDonald, Asa A Brockman, Rebecca A Ihrie, Lucy F Stead

**Affiliations:** Leeds Institute of Medical Research, University of Leeds, Leeds, UK; Leeds Institute of Medical Research, University of Leeds, Leeds, UK; Department of Neuropathology, Leeds Teaching Hospitals NHS Trust, Leeds, UK; Leeds Institute of Medical Research, University of Leeds, Leeds, UK; Leeds Institute of Medical Research, University of Leeds, Leeds, UK; Leeds Institute of Medical Research, University of Leeds, Leeds, UK; School of Mathematics, University of Leeds, Leeds, UK; Department of Neuropathology, Leeds Teaching Hospitals NHS Trust, Leeds, UK; Department of Neuropathology, Leeds Teaching Hospitals NHS Trust, Leeds, UK; Leeds Institute of Medical Research, University of Leeds, Leeds, UK; Flow Cytometry Core Facility, Newcastle University, Newcastle upon Tyne, UK; Flow Cytometry Core Facility, Newcastle University, Newcastle upon Tyne, UK; Flow Cytometry Core Facility, Newcastle University, Newcastle upon Tyne, UK; Department of Cell & Developmental Biology, Vanderbilt University School of Medicine; Vanderbilt Brain Institute, Vanderbilt-Ingram Cancer Center, Department of Neurological Surgery, Vanderbilt University Medical Center, Nashville, Tennessee, USA; Pediatrics – Section of Child Neurology, University of Colorado Anschutz Medical Campus, Aurora, Colorado, USA; Department of Cell & Developmental Biology, Vanderbilt University School of Medicine; Vanderbilt Brain Institute, Vanderbilt-Ingram Cancer Center, Department of Neurological Surgery, Vanderbilt University Medical Center, Nashville, Tennessee, USA; Leeds Institute of Medical Research, University of Leeds, Leeds, UK

**Keywords:** glioblastoma, GBM, IDHwt, TME, imaging mass cytometry, IMC

## Abstract

**Background:**

Glioblastoma (GBM), the most aggressive adult brain cancer, comprises a complex tumor microenvironment (TME) with diverse cellular interactions that drive progression and pathobiology. The aim of this study was to understand how these spatial patterns and interactions evolve with treatment.

**Methods:**

To explore these relationships, we employed imaging mass cytometry to measure the expression of 34 protein markers, enabling the identification of GBM-specific cell types and their interactions at the single-cell protein level in paired primary (pre-treatment) and recurrent (post-treatment) GBM samples from five patients.

**Results:**

We find a significant post-treatment increase in normal brain cells alongside a reduction in vascular cells. Moreover, despite minimal overall change in cellular diversity, interactions among astrocytes, oligodendrocytes, and vascular cells increase post-treatment, suggesting reorganization of the TME. The GBM TME cells form spatially organized layers driven by hypoxia pre-treatment, but this influence diminishes post-treatment, giving way to less organized layers with organization driven by reactive astrocytes and lymphocytes.

**Conclusions:**

These findings provide insight into treatment-induced shifts in GBM’s cellular landscape, highlighting aspects of the evolving TME that appear to facilitate recurrence and are, therefore, potential therapeutic targets.

**Key Points**

- Spatial organization in primary GBM consists of layers driven by hypoxia.
- The layers in recurrent GBM are driven more by the presence of reactive astrocytes.
- Increased cellular cross-talk in recurrent GBM presents novel therapeutic targets.

**Importance of the Study**

To fully understand glioblastoma requires us to not only recognize the distinct niches within its tumor microenvironment but also the complex interactions that sustain them and allow them to proliferate. Previous spatial studies in GBM have identified cellular neighborhoods and key interactions, including those between immune and GBM cancer cells. More recently, the GBM tumors have been shown to organize into structured regions defined by a five-layer architecture, with hypoxia playing a central role. Building on this work, here we show that this layered organization appears to be lacking in recurrent GBM, wherein interactions between the tumor cells and non-malignant brain cells seem to become more prevalent.

Furthering this understanding has far-reaching implications for improving patient outcomes by developing better treatments; optimizing current immunotherapies and refining models so that they more accurately reflect GBM evolution post-treatment.

Isocitrate dehydrogenase (IDH)-wildtype glioblastoma (GBM) is the most common and aggressive form of adult diffuse glioma, with a median survival of ~15 months.^1^ Standard treatment consists of surgical resection followed by radiation and chemotherapy with temozolomide.^2^ However, tumor recurrence is inevitable due to (a) the infiltrative nature of primary GBM, which precludes complete surgical removal and (b) significant intra- and inter-tumor heterogeneity, which enables residual cells to resist chemoradiation and continue proliferating.^3,4^ Characterizing how unresected GBM cells respond to treatment can highlight potential mechanisms of treatment resistance that could be additionally targeted with combined therapies.

It is known that IDH-wildtype GBM cells exhibit plasticity across four neoplastic cell states along a proneural to mesenchymal axis^5–7^: neural progenitor-like (NPC), oligodendrocyte progenitor-like (OPC), astrocyte-like (AC), and mesenchymal-like (MES). However, these neoplastic cells do not function in isolation. In their updated hallmarks of cancer, Hanahan and Weinberg remarked that any understanding of tumors “must encompass the contributions of the tumor microenvironment (TME).”^8^ In GBM, the TME comprises a diverse array of tumor cells and also a complex network of immune cells, stromal cells, and vascular elements that play a critical role in GBM progression and treatment resistance, acting as a dynamic ecosystem that influences tumor behavior and therapeutic response.^9^

To truly understand GBM tumor response to treatment, therefore, requires characterization at single-cell level in ways that incorporate information about interactions with the TME. This is now possible through the use of spatial molecular profiling technologies.^10^ Such approaches have recently been applied to GBM tumors, revealing niches containing specific neoplastic cells and distinct immune-associated programs.^11–13^ These niches have also been shown to organize into structured layers, beyond what is visible via conventional microscopy and histopathology, and are associated with cellular states such as hypoxia.^11^

These findings describe consistent organizational patterns across GBM tumors, suggesting that neoplastic phenotypes are driven by environmental interactions. However, one crucial aspect remains unexplored: how the spatial patterns and interactions within the TME are impacted by treatment to enable some neoplastic cells to survive. To begin to address this, we analyzed multiplex imaging mass cytometry (IMC) data,^14^ from five paired pre- and post-treatment IDH-wildtype GBM patient samples, focusing on protein-level changes that reveal alterations in cellular prevalence and states. This extends previous studies by analyzing longitudinal samples to try and understand how cellular landscapes are altered at recurrence and provide insights into tumor adaptation. Our use of spatial proteomics enables profiling at single-cell resolution (in contrast to previous results from spot-based transcriptomics, which profiles, on average, 8 cells simultaneously) using more stably expressed markers that have been validated through decades of use in the analogous approach of immune-histochemistry (IHC). The trade-off is that spatial proteomics is limited to significantly fewer marker genes with which to accurately assign cell types. To account for this, we have mapped our findings to previous studies, but also taken advantage of the significant advances in technologies in the past 12 months to perform spatial transcriptomics profiling of ~6000 genes at single-cell resolution in two of the same paired samples that we profiled via IMC, and one independent pair. This has enabled us to further probe key findings using an orthogonal approach.

## Materials and Methods

For full details, please see the Supplementary Methods.

### Imaging Mass Cytometry (IMC) Analysis

Paired glioblastoma (GBM) samples from five patients were analyzed at primary surgery and first recurrence as per Supplementary Figure 1 (30 total regions of interest [ROIs]; 3 per sample)^15^. A 34-antibody panel was validated on control tissues (Supplementary Table 3) and used to stain 5 µm FFPE sections. IMC data were acquired via laser ablation (1 µm, 200 Hz), and resulting MCD files were exported in OME-TIFF format.

Image processing and downstream analyses were performed using R (≥ 4.3.0) and Python (3.11.3). Cell–cell interactions and spatial contexts were computed using imcRtools (v1.10.0); visualizations were created using ggplot2 (v3.5.1).

### Cell Segmentation

Raw MCD files were converted to multi-channel TIFFs using Steinbock^16^ (v0.13.5), then cropped into 100 µm² sections. Nuclear (Ir191/Ir193) and cytoplasmic channels (Sm149, Eu153, etc.) were merged into RGB images. Segmentation was performed using Cellpose^17^ (v2.0) with the “cyto2” model, refined iteratively. The final model was applied to 1000 µm² ROIs. Mean intensity values and spatial features were extracted using Steinbock’s measurement functions.

### Single-Cell and Image Processing

Single-cell expression values were transformed using the asinh function (cofactor = 5), and batch effects across patients were corrected using Harmony.

### Cell Phenotyping

Cells with broadly high marker expression were excluded. Remaining cells were z-score normalized and ranked by expression level. Cell types were assigned using a logical gating strategy (Supplementary Table 4) based on marker-specific thresholds. Hypoxia (HIF1A+) and EMT (SNAI1+) statuses were classified using z-score cutoffs (<−1.2 or > 1.2).

### Intra-patient Heterogeneity

Shannon entropy was used to quantify cell type diversity across ROIs, calculated using 1,000 randomly sampled cells per ROI over 10 iterations. Entropy values were compared between primary and recurrent samples using the Wilcoxon rank-sum test.

### Spatial Analyses

Spatial interaction graphs were created using Delaunay triangulation (imcRtools)^18^ and pruned (max_dist = 50 µm). Cell–cell interactions were tested using test interactions, comparing observed counts against a null model via permutation testing.

### Cell Neighborhoods and Spatial Contexts

Neighborhoods were defined based on local cell-type proportions (aggregateNeighbors), then clustered using k-means (k = 12). Spatial contexts (SCs) were inferred using detectSpatialContext and filtered to retain dominant SCs (> 3 patients and > 5% of total cells per group)^19^.

### Spatial Transcriptomics

GBM tissue microarray (TMA) cores were profiled using the NanoString CosMX platform with the Human 6k RNA Panel. FFPE sections were stained with IF markers and imaged at subcellular resolution. Fields of view were placed using H&E guidance.

### Transcriptomic Cell Phenotyping

Cells were segmented using machine learning on IF images. Transcripts were background-corrected, normalized, and low-quality cells were excluded. Dimensionality reduction (UMAP) and clustering (Leiden, InSituType) were performed on high-variance genes. Clusters were annotated via marker gene expression and spatial localization.

### Mapping to Greenwald Metaprograms (MPs)

Top 20 marker genes per cluster were used for enrichment analysis against MPs defined by Greenwald et al. (2024). Cluster-MP assignments were confirmed via z-scored average expression profiles and adjusted DE gene sets (*P* < .01), yielding robust biological mappings.

## Results

### Identifying and Labeling Cell Types in GBM

**Figure 1.**
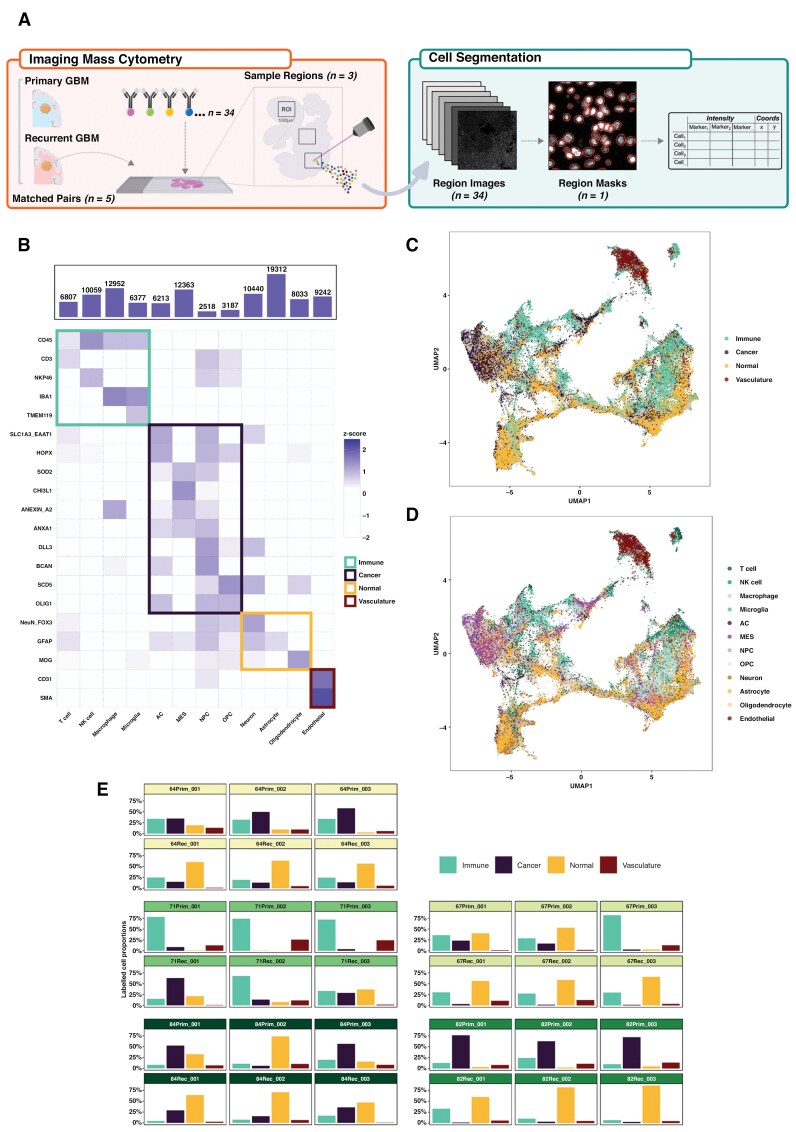
Cell segmentation and phenotyping overview. **(A)** Schematic detailing the imaging mass cytometry (IMC) process for one patient sample, including the downstream analysis steps comprising object segmentation and marker abundance quantification. (**B)** Heatmap of protein marker abundances (rows) for each of the labeled cell types (columns). The tile colors denote the scaled (z-score) marker intensities, and the tile highlight colors represent the four main cell categories. (**C–D)** UMAP of all (patients and surgeries) cell objects identified following segmentation, batch correction, and phenotyping: cells are colored by cell category (C) and cell type (D). (**E)** Proportion of labeled cell categories (columns) across each region of interest (ROI). The facets are grouped by patient/surgery, and each of the facet header colors denotes an individual patient.

To assess the spatial evolution of GBM tumors through treatment, we collected tumor samples from five patients who had undergone surgical resections of both primary and recurrent IDH-wildtype GBM. Each primary tumor developed de novo, and all patients received radiation, chemotherapy with temozolomide, and had a local recurrence. For patient and surgery information, see Supplementary Table 1 and Supplementary Figure 1. Three spatially distinct 1 mm^2^ regions of interest (ROIs) were selected for each tumor sample, following immunohistochemical staining for key markers of proliferation (Ki67), hypoxia (HIF1A), and immune cells (CD45), to capture intra-tumor heterogeneity and avoid the bias of examining only a single small region (Figure 1A, Supplementary Figure 2 and Supplementary Table 2).

We designed a panel of 34 protein markers to identify GBM-specific cell types (neoplastic, immune, and normal brain cells) along with markers of cell states such as proliferation and hypoxia (see Supplementary Table 3). Using a deep learning-based image segmentation approach, we assigned cell type labels to each segmented object and also subsequently grouped cells into four categories: immune, cancer, normal brain, and vasculature (Figure 1B). Approximately 107,000 cells were labeled across all samples (Figure 1C–D) after applying batch effect correction to account for variability between individual patients and to ensure that expression profiles were comparable (Supplementary Figures 4–5).

To ensure that our approach was accurately delineating GBM cells, we performed two independent reviews. Firstly, we extracted and sequenced RNA within corresponding regions of each tumor from consecutive FFPE sections and performed cellular deconvolution of bulk RNAseq.^15^ This showed significant concordance with the spatial proteomics across both neoplastic and immune cell types.^15^ Secondly, we asked a neuropathologist to independently quantify immune and vascular cells within all 30 ROIs, and compared this to the quantification from our automated pipeline. There was a significant correlation in score for both immune (*r* = 0.55, *P* = .02) and vascular (*r* = 0.84, *P* = 5.8e-9). The more moderate correlation in immune scoring was discussed with the pathologist, who believed it was likely owing to their own subjectivity in relation to thresholding/apparent intensity, and distinguishing true staining from artefact. This gave us confidence in our approach. However, we also performed spatial transcriptomics, using the Nanostring CosMX 6k panel, of distinct regions within paired samples from two patients for whom we had IMC (patients 64 and 84) and one additional patient (patient 40). We used these data to further investigate some key findings from the proteomics results, as relayed throughout this section, where relevant.

A comparison of cell categories across each ROI (Figure 1E) showed surprisingly consistent within-sample distributions, confirming that there is intra-tumor TME heterogeneity but that this is not as significant as inter-tumor TME heterogeneity. ANOVA analysis (see Supplementary Table 5) confirmed that the effect of patient and surgery was significant for all cell types (*P* < .001), indicating considerable inter-tumor heterogeneity. In contrast, intra-tumor heterogeneity, represented by differences across ROIs, was not significant for any cell category, suggesting that intra-tumor TME variability is less pronounced compared to inter-tumor heterogeneity. Therefore, we combined the three ROIs per sample, prior to subsequent downstream analyses, to increase the number of cells per sample whilst minimizing sampling bias from specific regions.

### Alterations in Cellular Prevalence Through Treatment in GBM

**Figure 2.**
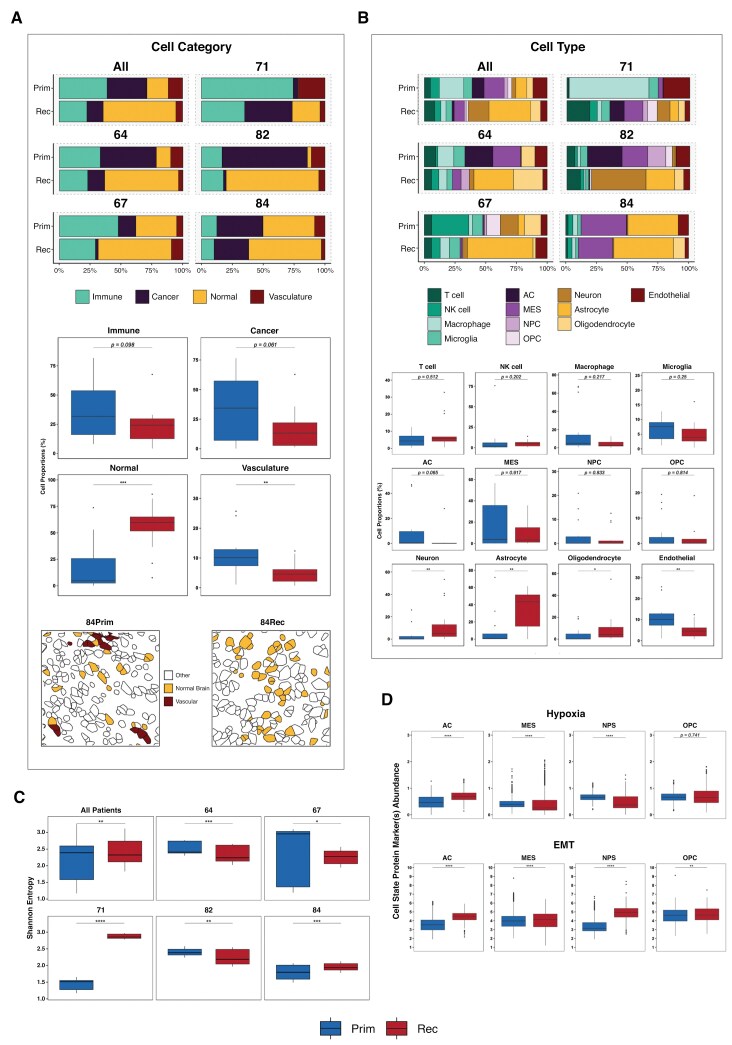
Changes in GBM cell categories and types through treatment. (**A)** Top: stacked bar charts showing the labeled cell category prevalences across all patients (top left) and also separately for each individual patient. Middle: boxplots showing the distribution of each cell category proportion across all patients grouped by primary and recurrent surgeries. Bottom: representative spatial transcriptomic (CosMX) images showing single-cell segmentation masks colored by normal brain and vasculature cell category labels for primary (left) and recurrent (right) surgery from one patient (patient 84). Cells with white fill color represent cell categories other than normal brain or vasculature. (**B)** Top: stacked bar charts showing the labeled cell type prevalences across all patients (top left) and also separately for each individual patient. Bottom: boxplots showing the distribution of each cell type proportion across all patients grouped by primary and recurrent surgeries. (**C)** Boxplots showing the distribution of Shannon’s entropy values grouped by surgery and split across all patients (top left) and also for each individual patient. (**D)** Boxplots, grouped by surgery, showing the distribution of protein marker abundance for markers that define hypoxia (top) and the epithelial-to-mesenchymal transition (bottom). The black horizontal boxplot lines represent the median, and the upper and lower box bounds denote the 25th and 75th quantiles, respectively. Astrocyte-like (AC); mesenchymal-like (MES); neural progenitor-like (NPC); oligodendrocyte progenitor-like (OPC); epithelial-to-mesenchymal transition (EMT). Significance thresholds: *P < .05; **P < .01; ***P < .001; ****P < .0001.

We first assessed how the prevalence of each cell category changed through treatment, between primary and recurrent samples (Figure 2A and Supplementary Table 6). Whilst a reduction in the percentage of both immune and neoplastic cells was observed, from primary to recurrence, only the decrease in vasculature cells was significant (Wilcoxon *P* = .09, 0.06, and 9.88E-03, respectively). The only significant increase was in the proportion of normal brain cells (Wilcoxon *P* = 4.52E-04). These changes were further validated using a different spatial transcriptomics approach with the Nanostring CosMx platfrom (Figure 2A).

Drilling down into how specific cell types change through treatment showed that no individual immune cell type exhibited significant changes (Figure 2B and Supplementary Table 7). Similarly, no individual cancer cell types altered in a consistent direction, although the astrocyte-like (AC) cancer cell type showed the largest and most consistent decrease (Wilcoxon *P* = .065). All normal brain cell types showed significant increases during treatment, with astrocytes exhibiting a particularly notable increase from primary to recurrence (Wilcoxon *P* = 3.02E-03), which was consistent across each patient. Of note, astrocytes appear to be the most prevalent normal brain cell type overall, consistent with reports of their high prevalence in both normal brain and the GBM TME.^20,21^

These changes agree with those from our larger cohort studies, where we performed deconvolution from bulk RNAseq, validating our approach.^22,23^

The limited number of consistent, significant changes in the prevalence of cell categories or types over time highlights the variability in immune and neoplastic cell categories, post-treatment, across patients (Figure 2A–B). We, therefore, decided to systematically evaluate how cell diversity changes through treatment, both overall and at an individual patient level, to determine if any consistent patterns emerge.

### Alterations in Cellular Diversity Through Treatment in GBM

To inspect cellular diversity in our samples, we quantified the Shannon’s entropy (H) for each one (Figure 2C and Supplementary Table 8). A high Shannon’s entropy value indicates a tumor with many different cell types of similar frequency, whereas low entropy suggests that the tumor is dominated by few(er) cell types. This metric thus serves as a good proxy for assessing intra-tumor cellular heterogeneity for each sample, for example, pre- and post-treatment.

We found that, overall, Shannon’s entropy significantly decreased from primary to recurrence (Wilcoxon *q* = 3.93E-03, Figure 2C), suggesting that cell distributions become less diverse, likely owing to certain cell types becoming more dominant within the distribution at recurrence. Linking this back to the results in Figure 2A and B, this appears to be driven by the greater abundance of normal brain cells, and especially astrocytes, in the recurrent tumors. However, analysis of individual patients revealed variability in how cellular heterogeneity changed over time. Two patients (71 and 84) had significantly increased diversity through treatment (Wilcoxon *q* = 8.45E-17 and 7.10E-04, respectively, Figure 2C). In patient 84, this increase was primarily driven by the appearance of oligodendrocytes at recurrence, which weren’t present in the primary tumor (Figure 2B). Conversely, for patient 71, the increase in entropy was associated with a reduction of dominating macrophages in the primary and presence of a larger neoplastic and normal brain cell fraction at recurrence (Figure 2B).

Given a lack of consistent trends in how treatment affects cell type prevalence or dominance, we proceeded to investigate whether changes in cell state could indicate how treatment shapes cancer cell phenotypes.

### Alterations in Neoplastic Cellular States Through Treatment in GBM

The mesenchymal (MES) phenotype in GBM cancer cells is characterized by high proliferative and metastatic potential, often leading to a poorer prognosis compared to proneural subtypes.^24–27^ Moreover, elevated hypoxia and the expression of epithelial-to-mesenchymal transition (EMT) genes, typically involved in neural tube formation or wound healing, have been shown to be closely linked to the MES cell state.^28^

In our IMC panel, we included antibodies against proteins indicating hypoxia (HIF1A) and epithelial to mesenchymal transition (SNAI1 & TGFBeta) to assess the proportion of each of the four identified neoplastic cancer cell types that are in these cellular states, and how they changed through treatment. We found that significantly more AC cancer cells expressed hypoxia markers post-treatment (Wilcoxon *P* = 4.98E-115), whilst significantly fewer MES and NPC cells did (Wilcoxon *P* = 9.62E-125 and *P* = 5.49E-70, respectively) (Figure 2D and Supplementary Table 9).

All four neoplastic cell types had a significantly higher proportion of cells expressing markers of EMT post-treatment, with the largest effect sizes observed in AC and NPC cells (Wilcoxon *P* = 5.29E-161 and *P* = 2.32E-183, respectively).

The power of our approach is not just in inspecting paired longitudinal GBM samples at single-cell resolution but also describing how treatment alters the cellular landscape in terms of spatial context. Hence, we moved on to looking at in situ cellular interactions, that is, cells directly adjacent to one another.

### Alterations in Cellular Interactions Through Treatment in GBM

**Figure 3.**
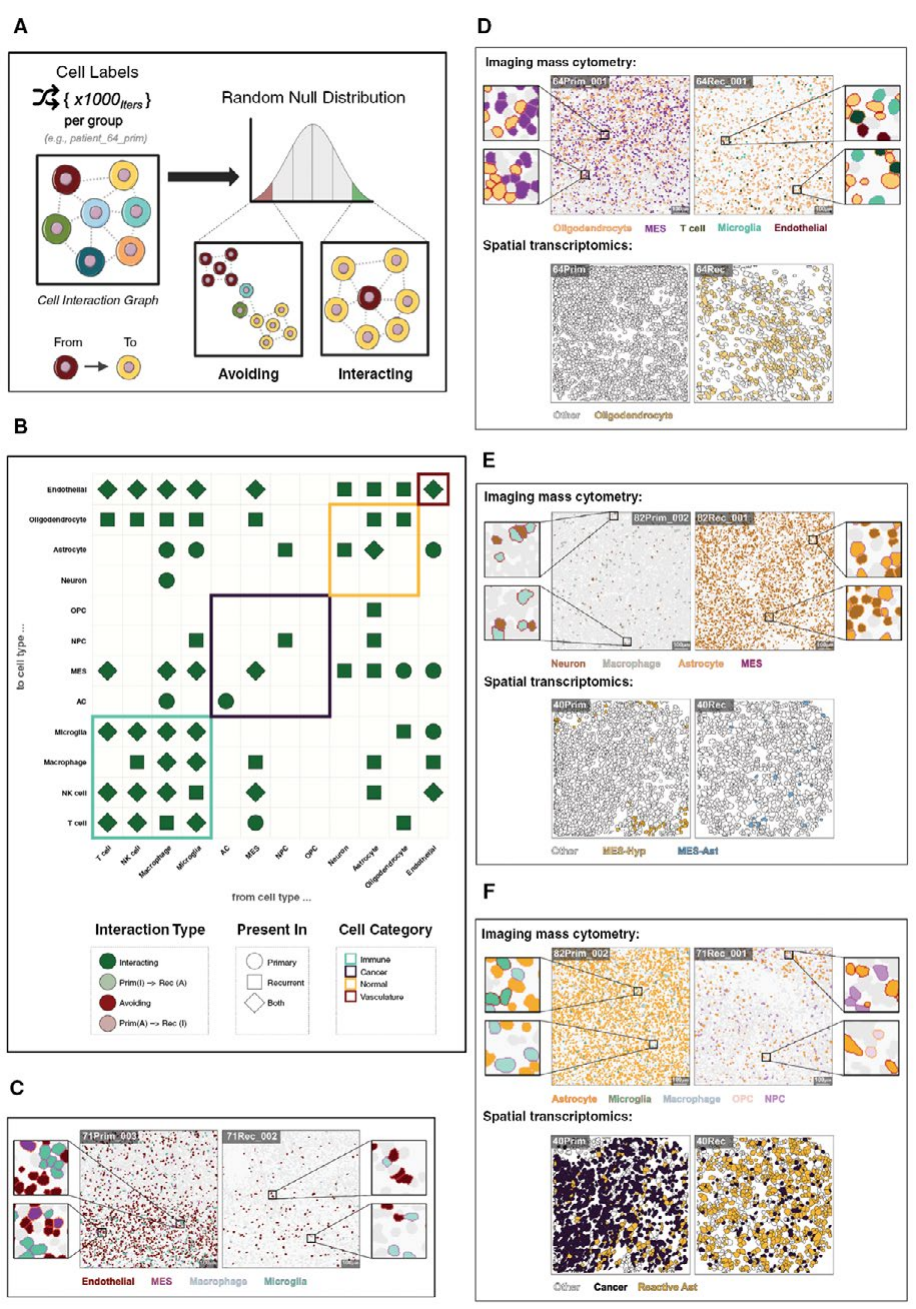
Identifying significant GBM cell–cell interactions through treatment. **(A)** Schematic showing the process for testing if cell types interact more or less frequently than random. Each cell–cell interaction (edge) from an interaction graph is counted and averaged across a defined group (eg, patient, surgery, etc.). These average cell–cell interactions are then divided by the number of cells of type A that have at least one neighbor of type B. Finally, each observed cell–cell interaction count is compared against a null distribution that is generated by shuffling the cell-type labels 1000 times (1000 iterations) and counting the interactions between two specific cell types, giving the interaction counts under spatial randomness. Two cell types are “avoiding” when there are significantly fewer interactions compared to random expectation for a given *P* value threshold. Conversely, when there are significantly more interactions between the two cell types, they are “interacting.” (**B)** Dotplot showing the significant (*P* < .01) cell–cell interactions that are present across a minimum of three patients. Shape denotes whether a specific cell–cell interaction is significant across either primary, recurrent, or both surgeries. Point colors denote the type of significant interaction, that is, interacting/avoiding, and also cases where the interaction type changes through surgery. The tile highlights denote the cell category of each cell type. (**C)** Representative IMC images showing single-cell segmentation masks colored by the corresponding cell type labels for primary (left) and recurrent (right) surgery regions of interest. (**D**–**F) Top:** Representative IMC images showing single-cell segmentation masks colored by the corresponding cell type labels for primary (left) and recurrent (right) surgery regions of interest. Bottom: representative spatial transcriptomic (CosMX) images showing single-cell segmentation corresponding with cell categories and types. The “other” white filled cells denote cells which do not correspond to the respective labels shown. The Reactive-Ast, MES-hyp, and the MES-Ast cell types correspond to those identified in Greenwald et al. 2024.^11^

We evaluated pairwise cell–cell adjacency, serving as an indicator of cell interaction partners, to assess whether any were significantly more likely (cells are “interacting”) or less likely (cells are “avoiding”) compared to the null hypothesis of spatial randomness (Figure 3A and Supplementary Tables 10 and 11). Herein, these cellular localizations are referred to as “interactions” to remain consistent with previously used terminology^11^; however, this does not imply any direct or mechanistic interactions between the cell types.

We performed this analysis on the primary and recurrent samples separately to see which significant findings were timepoint dependent. Many cell types predominantly interacted with themselves in the primary tumors (Figure 3B). This is in keeping with previous spatial analysis of GBM that used a “spot-based” technology, which is not resolved at the single-cell level but rather aggregated over a small defined area (spot), which found that signal from the majority of spots seemed to emanate from a single cell type.^11^ Our expansion to recurrent samples shows that these “self” interactions remained consistent through treatment (Figure 3B). Two additional, clear observations from our results are that there are no cells significantly avoiding one another, and there are many more recurrence-specific, significant cell–cell interactions than primary-specific ones (27 versus 10). Hence, despite finding an overall reduction in cell diversity at recurrence (Figure 2C), there are more interactions between differing cell types, suggesting that these are non-random and, thus, phenotypically important.

#### Neoplastic cells

Amongst the GBM cancer cell types, MES cells formed the highest number of significant interactions with other cell types. MES interactions with immune cells remained consistent between paired samples, but interactions with normal brain cells were increased at recurrence.

#### Vasculature

Despite decreasing through treatment (Figure 2A), endothelial cells still formed significant interactions at both time points (Figure 3C). Unique to the primary tumors were significant interactions from the endothelial cells to the microglia (permutation test, *P* = 9.99E-04) and MES cancer cells (permutation test, *P* = 9.99E-04). MES cells interacting with myeloid lineage cells (eg, macrophages and microglia) have been shown to lead to a highly proliferative state, increasing angiogenesis and contributing to a more invasive phenotype, which may explain these findings in the primary tumor.^29^ Moreover, these interactions have also been shown to induce chemoresistance in GBM, which has the potential to be addressed therapeutically.^30^

The interactions from the endothelial cells to the macrophages were particular to recurrent tumors (permutation test, *P* = 9.99E-04). These findings could be visualized in the IMC data (Figure 3C), where a clear reduction in endothelial cells over time coincided with changes in the cells interacting with the remaining vasculature. It has previously been shown that bone-derived macrophages populate a GBM tumor post-treatment, via the vascular system, which may explain this result and further indicate that therapies which hijack this infiltration could be effective for preventing or prolonging GBM recurrence.^31^ Interactions from all normal brain cells to endothelial cells were also specific to the recurrent tumor. The post-treatment increase in normal brain cell abundance within the resected tissue (Figure 2A) may reflect the brain’s wound healing response, with neuronal and glial cells re-populating the void left by surgery.^32^

### Normal Brain Cells

Significant interactions from and to oligodendrocytes almost universally occurred in the recurrent tumors, barring those from oligodendrocytes to MES cancer cells, which were primary-specific (Figure 3B). This could be observed in the IMC visualizations and also further validated using spatial transcriptomics (Figure 3D). The prevalence of oligodendrocytes increases from primary to recurrent (Figure 2B), suggesting that this population did not simply expand in situ but rather infiltrated the recurrent TME. In GBM, oligodendrocyte lineage cells have commonly been reported to reside at tumor border niches, including the invasion front/resection border, where they co-localize with macrophages/microglia.^33^ Moreover, oligodendrocytes have been shown to support GBM tumorigenicity and migration by promoting angiogenesis in GBM.^34,35^ We also found evidence supporting the model of interactions, as microglia and endothelial cells were significantly interacting with oligodendrocytes at recurrence (Figure 3D).

Of all the normal brain cells, astrocytes were found to significantly interact most frequently and significantly with the cancer cells, though this is mostly specifically at recurrence. In fact, aside from “self” interactions, which were consistent through treatment, normal astrocytes only formed significant interactions during recurrence.

Crosstalk between microglia and macrophages is known to induce reactive astrocyte phenotypes, which are crucial for the brain’s wound healing process—a key aspect in GBM.^36,37^ Moreover, the MES phenotypes, as described by Wang et al. and Neftel et al., have been shown to overlap significantly with the presence of reactive astrocytes, indicating that these cells may migrate to injury sites after resection as part of the healing process.^32^ In our samples, we found significant interactions between normal neurons and astrocytes, suggesting the activation of cellular programs that could restore normal tissue function (Figure 3E and 3F). Moreover, these findings were further validated using spatial transcriptomics, which allowed us to refine the identification of a reactive astrocyte phenotype based on the meta-module described by Greenwald et al.^11^ We also observed treatment-associated changes in the two MES-like cancer cell states defined in their study: one linked to hypoxia (MES-Hyp) and the other to reactive astrocytes (MES-Ast) (Figure 3E). Leveraging this approach and the expanded set of markers available for cell phenotyping, we were further able to assess the dynamics between cancer cells and reactive astrocytes, where we found the latter to be increasing through treatment (Figure 3F).

### Alterations in Cellular Neighborhoods Through Treatment in GBM

**Figure 4.**
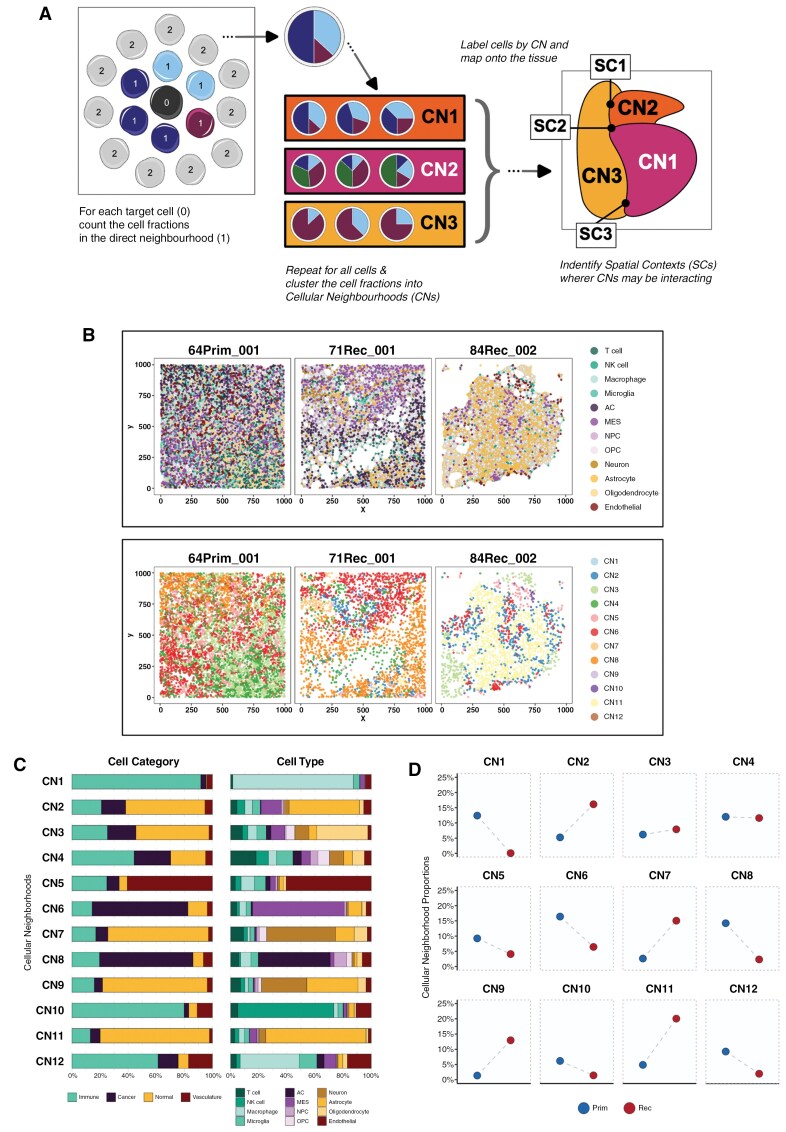
Identifying distinct cellular neighborhoods present across GBM samples through treatment. (**A)** Schematic showing the process of identifying cellular neighborhoods (CNs): each cell's direct neighborhood (as defined by an interaction graph), cell fraction is aggregated and clustered across each patient/surgery. The resulting CN cluster labels are then mapped to each cell object. The spatial contexts (SCs) are then defined as locations where the most dominant CNs interactions are also interacting. (**B)** Plots showing the single-cell spatial locations of three representative patient/surgery sample regions of interest visualized as nodes on a two-dimensional plane, with cell–cell interactions shown in the form of undirected edges between nodes (top). The nodes are colored according to the cell type label (top) and also by the cellular neighborhoods they belong to (bottom). (**C)** Stacked bar charts showing the proportion of each cell category (left) and cell type (right) that is present across each CN (rows). (**D)** Dot plots showing the relative proportion of cells in each of the CNs (facets) across each surgery type..

Cell interactions within the GBM TME are heavily influenced by the spatial context, as GBM tumors consist of distinct anatomical regions.^38^ To generalize groups of interacting cell types, we defined cellular neighborhoods (CNs) using a nearest-neighbor approach (Figure 4A). These cellular neighborhoods refer to recurring patterns or groupings of cell types that tend to co-localize together within the GBM TME, and often reflect functional or biologically significant units.

This method defined 12 distinct cellular neighborhoods that provided a different level of structure from that observed based just on individual cells (as exemplified in Figure 4B). As expected, owing to the fact that each cell significantly associates with itself in both the primary and recurrent tumors (Figure 3B), we found that most cell neighborhoods are dominated by a specific type (Figure 4C). CNs capture multiple cells in close proximity (Figure 4A), so are akin to the information captured by spot-based spatial technologies such as the 10X Visium platform. Our finding of dominance of a given cell type in each defined CN agrees with Greenwald et al.’s recently published results from application of the Visium platform to primary GBM samples.^11^ Extending these results using imaging mass cytometry, which provides single-cell resolution, we can further see that this dominance rarely equates to more than 50% of the cell types in a given CN, meaning there is clear ad-mixture and heterogeneity in interacting cells even when signal from one type predominates (Figure 4C).

Greenwald et al proceeded to cluster their spot-based gene expression profiles into 16 “metaprograms” (MPs). These programs are derived in an unsupervised manner and represent recurrent transcriptional profiles that are present across multiple cells, tissues, or conditions. Moreover, they reflect shared biological processes such as hypoxia and proliferation and also cellular states such as reactive astrocytes. Our CNs map to these MPs (Table 1 and Supplementary Table 12), though with some differences due to the level of cellular resolution and the differences in dimensionality and modality between the two studies. Specifically, Visium spots capture signals from 1–35 cells, so some MPs result from more than just nearest neighbors; and MPs are derived from gene expression (typically 7000 parameters our CNs derive from protein expression (34 parameters). It is worth noting that we aligned both CN4 (T-cell dominated) and CN10 (NK cell dominated) with the T-cell MP, owing to the functional similarities between T- and NK-cells.^39^

**Table 1.** Mapping of Previously Defined Spatial GBM Metaprograms to the Cellular Neighborhoods Defined in This Study.

Greenwald et al Metaprogram (MP)	Metaprogram description	Cellular neighborhood (Figure 4C)
MES-Hyp	Hypoxic mesenchymal cancer cells	CN6
MES-Ast	Astrocytic-like mesenchymal cancer cells	CN2
MES	Mesenchymal (other) cancer cells	
OPC	Oligodendrocyte progenitor cell-like cancer cells	CN3
AC	Astrocytic like cancer cells	CN8
NPC	Neural progenitor cell-like cancer cells	
Oligo	Oligodendrocytes	CN3
Neuron	Neurons	CN7 & CN9
Reactive Ast	Reactive astrocytes	CN9 & CN11
Inflammatory Mac	Inflammatory macrophages	CN12
Mac	Macrophage and microglia	CN1
T-cell	T-cells	CN4 & CN10
B-cell	B-cells	
Vasc	Vasculature	CN5
Chromatin-Reg	Chromatin regulation	
Prolif-Metab	Proliferation and metabolism	

Having aligned with previous findings from GBM tumors at a single time point, we wished to see how the prevalence of CNs changes over time. We see that certain CNs increased in abundance from primary to recurrent tumors, and others decreased (Figure 4D). Primary samples were enriched in neighborhoods that included immune cells, particularly macrophages (CN1 and CN12) and lymphocytes (CN10), vasculature (CN5), hypoxic MES (CN6), and AC cancer cells (CN8). In contrast, the recurrent surgery samples were enriched in neighborhoods dominated by normal brain cells: astrocytes (CN9 and CN11); neurons (CN7); and oligodendrocytes (CN3). Interestingly, we found that whilst hypoxic mesenchymal-driven CN6 decreased, astrocytic-like mesenchymal-driven CN2 was increased from primary to recurrence.

Ultimately these results reconfirm what was seen when looking at cell type or category prevalences in isolation (Figure 2), that is, that immune cell-driven (CN1, CN10, and CN12), vascular-cell driven (CN5) and cancer cell-driven (CN6 and CN8) neighborhoods decreased from primary to recurrence, whereas normal brain cell driven (CN2, CN3, CN7, CN9, and CN11) neighborhoods increased. CN4, which was dominated by T-cells, changed least in prevalence over time.

Greenwald et al.’s seminal finding was that, in some primary GBMs, MPs form organized layers that result in a global tumor architecture, which is seemingly driven by the presence of hypoxic niches. We, therefore, proceeded to investigate whether this organization was evident in our primary samples and whether it was maintained post-treatment.^11^

### Alterations in Spatial Organization Through Treatment in GBM

**Figure 5.**
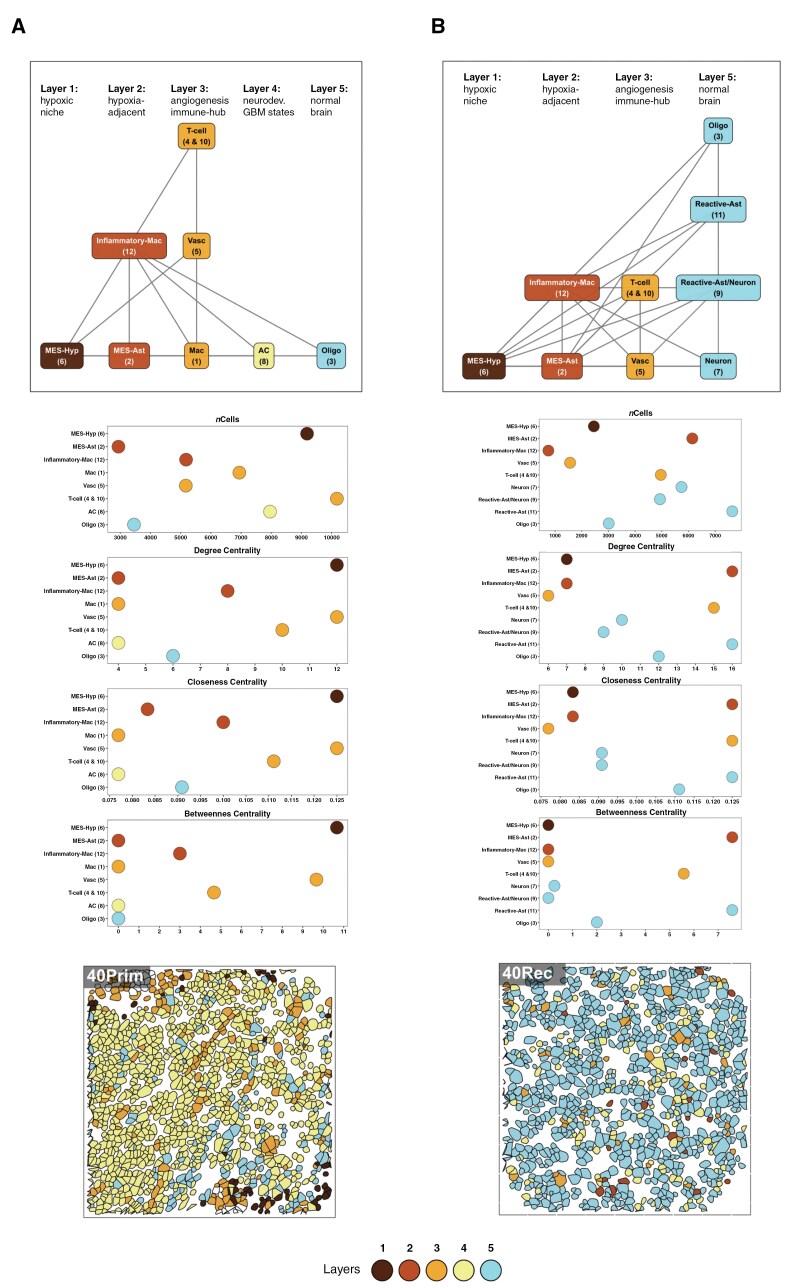
Spatial organization of cellular neighborhoods across surgeries. (**A**–**B)** Top: network graphs with nodes labeled according to the cell metaprograms identified in Greenwald et al. (2024) along with their corresponding cellular neighborhoods (shown in the brackets below). The edges represent the most dominant interactions present across primary surgery (A) and recurrent surgery (B) regions of interest (ROI), respectively. Middle: dot plots showing the number of cells present across each surgery-specific cellular neighborhood, and also three network-specific centrality measures: degree, closeness, and betweenness. Bottom: representative spatial transcriptomic (CosMX) images showing single-cell segmentation masks colored by the Greenwald et al. layers for primary (left) and recurrent (right) surgery from one patient sample. Cells with white fill do not align to any of the corresponding layers. Both network graphs and their corresponding metrics are colored and ordered according to the structured GBM spatial layers described in Greenwald et al. (2024).^11^

To better understand higher-order structuring of our cellular neighborhoods, we classified spatial contexts (SCs); locations where distinct cellular neighborhoods were found to consistently interact (Figure 4A). When considering the most dominant CN interactions present across primary and recurrent surgeries, our results reproduce similar ordered layers to those reported in Greenwald et.al.^11^ However, the prevalence and importance of states which make up the layers differs greatly through treatment, as revealed by the structure and parameters of the calculated CN interaction networks (Figure 5).

In the primary samples, the most influential and prevalent cellular neighborhoods were those characterized by layers 1 and layers 3, which denote the hypoxic/necrotic core niche and the angiogenesis-immune hub, respectively (Figure 5A). These layers were comprised of CNs with high network centrality scores across three key measures: degree (the number of direct connections a CN has to others, indicating its interaction density); closeness (how centrally positioned a CN is based on its average distance to all other CNs); and betweenness (the extent to which a CN lies on the shortest paths between other CNs, reflecting its role as a bridge between other CNs). This concurs with previous findings suggesting that hypoxia potentially drives the presence of the organized layers owing to phenotypic consequences of reduced oxygen, especially at the tumor core.^11^

Conversely, in recurrent samples, there were many more significant interactions between CNs in different layers (Figure 5B) in agreement with our findings from pairwise cellular interaction analysis (Figure 3B). Additionally, the most influential and prevalent cellular neighborhoods in recurrent samples were mostly in layers 2 and 5, which represented the hypoxia-adjacent and normal infiltrative brain layers (Figure 5B). This suggests a reduced global structure with less well-organized layers, potentially owing to a reduction in the presence of hypoxic niches in recurrent versus primary GBM.

Worth noting is that T-cell dominated CN4, which remained the most stably prevalent between primary and recurrent samples (Figure 4D), in combination with CN10 (together these CNs align to the previously denoted T-cell MP: Table 1) maintain high network parameters in both primary and recurrent GBMs (Figure 5), implying they are important in driving spatial contexts both pre- and post-treatment.

## Discussion

While we observe validated longitudinal changes in the GBM TME cellular architecture, we acknowledge that this was a small study (*n* = 5) and based primarily on correlational analyses. As such, we cannot definitively determine whether the observed changes are direct consequences of treatment or reflect the natural course of tumor evolution. Further studies with larger cohorts and experimental validation will be needed to clarify these dynamics. Moreover, the technical limitations of protein-based expression profiling confined the number of markers that could be used to assign cell types (*n* = 34). This meant that some cells (eg, B-cells) were not included, and there were fewer markers available with which to conclusively discern between neoplastic and normal brain cell types, which are closely related, or between different phenotypes of specific cell types (eg, M1 vs M2 polarization of macrophages).^6^ Notwithstanding, protein markers exhibit less stochastic expression and lower signal dropout (false negatives) compared to single-cell sequencing approaches. In this study, we utilized archival FFPE patient samples, which can exhibit variable preservation over time, and therefore, this is also a constraint of this study.

Our results reveal an influx of normal brain cells into the GBM microenvironment post-treatment, alongside a reduction in vascular cells (Figure 2A). The latter is expected, as surgery aims to debulk the highly vascularized tumor core.^40,41^ However, the reduction in endothelial cells in recurrent GBM suggests a reduced functional reliance on vasculature, which may explain the failure of angiogenic therapies like bevacizumab (Avastin) in clinical trials.^42^ It should be noted that although throughout this study we refer to stromal cells present within the GBM TME as “normal” brain cells, this is solely to distinguish them from malignant, neoplastic populations. In reality, these stromal cells reside in tumor-influenced brain parenchyma and likely exhibit altered phenotypes in response to the surrounding milieu.

Several large cohort studies that deconvoluted cellular signals from paired GBM using bulk and single-cell RNA-seq have reported an increased presence of oligodendrocytes at recurrence.^22,43,44^ Herein, we confirm this finding (Figure 2B) and further show that oligodendrocytes integrate into the GBM TME, as their interactions with other cells significantly increase at recurrence (Figure 3B). Oligodendrocytes are essential for cerebral homeostasis and regulate neuronal activity via axon myelination.^33^ We characterized oligodendrocytes using the myelin oligodendrocyte glycoprotein (MOG) marker (Figure 1B), suggesting that the increases we observe relate to myelinating oligodendrocytes integrating into the tissue and implying a potential functional role for myelination within the GBM TME post-treatment. Interestingly, the increased interactions and integration of oligodendrocytes we observed in recurrent tumors, primarily involve non-neoplastic cells. Among cancer cells, recurrence-specific interactions involving oligodendrocytes are restricted to MES neoplastic cells (Figure 3B). Oligodendrocytes have been shown to upregulate the invasive capacity of GBM cancer cells via Angiopoietin-2 signaling, and MES are the most invasive neoplastic subtype.^34,45^

We found that all neoplastic GBM cells showed increased epithelial to mesenchymal transition (EMT) at recurrence (Figure 2D). Moreover, the cellular neighborhood dominated by oligodendrocytes (CN3) had higher closeness and degree centrality at recurrence (Figure 5), indicating greater connectivity and interaction with other CNs. We propose that the role of oligodendrocytes in driving post-treatment recovery of GBM is worthy of further exploration.

Astrocytes exhibit the largest increase of normal brain cells (Figure 2B) within the recurrent GBM TME and also the highest number of recurrence-specific interactions, particularly with neoplastic cells (Figure 3B). Within the healthy brain parenchyma, astrocytes are crucial for neuronal cell homeostasis and also help drive the brain’s injury response by acquiring a reactive phenotype. Consistent with this role, cellular neighborhoods that map to the previously defined reactive astrocytic metaprogram (CN9 and CN11) and the astrocytic mesenchymal metaprogram (CN2) are increased at recurrence (Figure 4D and Table 1). Astrocytes also exhibit resistance to apoptosis triggered by death receptors during inflammation, such as apoptosis antigen 1 and TNF-related apoptosis-inducing ligands (FAS, TRAIL), indicating their resilience under inflammatory conditions. Together, this suggests a phenotypic response within the (infiltrating) astrocytic population of the TME that could serve to protect neoplastic cells.

Previous research has indicated that there is a shift toward a more mesenchymal state in bulk tumors at recurrence.^22^ Single-cell analyses have further refined this understanding, showing that, whilst some GBMs show an increase in MES cancer cells post-treatment, others show increases in more proneural (OPC and NPC) cells at recurrence.^46,47^ We also find that there is no significant, consistent change in neoplastic cell types at recurrence, but instead, a universal increase in EMT markers across all the neoplastic cell (Figure 2D). This potentially explains the shift to mesenchymal expression signatures observed from bulk tumor profiling.^22^

In keeping with our previous findings, AC-like cancer cells reduce most consistently at recurrence.^23^ However, the remaining AC-like cells had elevated levels hypoxia (Figure 2B and D), while these decreased within MES and NPC cell populations. Hypoxia can induce a reactive astrocyte phenotype within the TME, which may extend to AC-like cancer cells, potentially even promoting plastic conversion to this neoplastic subtype.^48,49^

Overall, we find no consistent changes in cellular diversity between primary and recurrent GBM (Figure 2C), suggesting that while cellular heterogeneity is maintained, post-treatment GBM tumor have greater interactions between differing cell types (greater admixture, Figures 3 and 4). A recent spatial profiling study of primary GBM tumors by Greenwald et al. concluded that hypoxia drives organization of a GBM architecture, composed of layers.^11^ Our findings concur with theirs for primary GBM but expand further, revealing that this layering is less structured post-treatment (Figure 5). The decrease in CN6, which maps to their hypoxic MES cancer cell metaprogram (Table 1), but an increase in CN2, which maps to their astrocytic MES cancer cell metaprogram (Table 1), at recurrence suggests that an overall reduction in hypoxia post-treatment could drive this increased disorder. This influx and integration of normal brain cells in the GBM TME at recurrence corresponds with these cells becoming much more influential in terms of the interaction between cellular layers, particularly CN11, which maps to the reactive astrocyte metaprogram of Greenwald et al. (Figure 5B).

Whilst lymphocyte abundance remains unchanged between primary and recurrent GBM (Figure 2B), neighborhoods (CN4 and CN10) mapping to the T-cell metaprogram (Table 1) become much more influential in the recurrent GBM (Figure 5B). T-cells and tertiary lymphoid structures (regions enriched in lymphocytes, resembling CN4 and CN10) have been shown to increase in subsets of paired primary and recurrent GBM,^46,50^ which has renewed interest in understanding their potential role in immunotherapy. In support of this, activated T-cells have been shown to associate specifically with astrocytic MES, which is the subtype we also find increased recurrent tumors.^24^

This study highlights prominent post-treatment changes in the GBM cellular landscape and offers novel insight into the importance of specific interactions between GBM cancer cells and the TME during tumor survival and regrowth. Within the last decade the characterization of how glioblastoma brain tumors interact with surrounding non-neoplastic neuronal and glial cells, in ways which impact tumor phenotype, have become central to the emerging field of cancer neuroscience.^51^ Our study indicates that these may be some of the most important cellular interactions that enable the tumor to survive standard treatment, and thus a further, more-mechanistic, exploration of them may yield important therapeutic targets.

## Supplementary material

Supplementary material is available online at *Neuro-Oncology* (https://academic.oup.com/neuro-oncology).

noaf190_Supplementary_Figures_1-5_Tables_1-12

## Data Availability

The data used in this study (including the raw high-dimensional TIFF images, spillover correction files; cell-object segmentation masks; patient and sample metadata; phenotype labeled single-cell data and the cell-object spatial information) are deposited and available online at Zenodo: (https://doi.org/10.5281/zenodo.14679442). The analysis code that was used to process the IMC data and produce the results in this paper can be found on GitHub: (https://github.com/GliomaGenomics/GBM_IMC_Analysis)
