# Supplementary material for: Spatial profiling of longitudinal glioblastoma reveals consistent changes in cellular architecture, post-treatment
Source: Neuro Oncol. 2025 Sep 1;27(12):3145–60. doi: 10.1093/neuonc/noaf190 (PMC12916744; doi:10.1093/neuonc/noaf190)
Supplement: noaf190_Supplementary_Figures_1-5_Tables_1-12 [file noaf190_supplementary_figures_1-5_tables_1-12.pdf]

Supplementary Information

Spatially charactering IDH-wildtype glioblastoma tumours pre- and post-treatment using multiplex imaging mass cytometry

Shoaib Ajaib[1], Steven Pollock[1], Gemma Hemmings[1], Arief Gusnanto[2], Aruna Chakrabarty[3], Azzam Ismail[3], Erica Wilson[1], Bethany Hunter[4], Andrew Filby[4], David McDonald[4], Asa Brockman[5], Rebecca Ihrie[5], Lucy F. Stead[1*]

[1]Leeds Institute of Medical Research, University of Leeds, Leeds, UK
[2]School of Mathematics, University of Leeds, Leeds, UK
[3]Department of Neuropathology, Leeds Teaching Hospitals NHS Trust, Leeds, UK
[4]Flow Cytometry Core Facility, Newcastle University, Newcastle upon Tyne, UK
[5]Department of Cell & Developmental Biology, Vanderbilt University School of Medicine; Vanderbilt Brain Institute, Vanderbilt-Ingram Cancer Center, Department of Neurological Surgery, Vanderbilt University Medical Center, Nashville, Tennessee, United States

This file contains the supplementary figures and tables that are detailed in the above paper.

Supplementary Table 1. Clinical and molecular data of patients included in the study.

patient	sex	primary tumour location	diagnosis age	PFS (months)	OS (months)	status (1=deceased;0=alive)	responder type
64	Male	Frontal	57	22.55	48.76	1.00	up
67	Male	Temporal	51	40.73	75.19	1.00	up
71	Female	Temporal	60	17.10	39.12	1.00	down
82	Female	Parietal	59	23.24	33.21	1.00	up
84	Female	Parietal	72	17.49	45.76	1.00	up

Supplementary Figure 1. Survival time-events associated with the patients in the study.

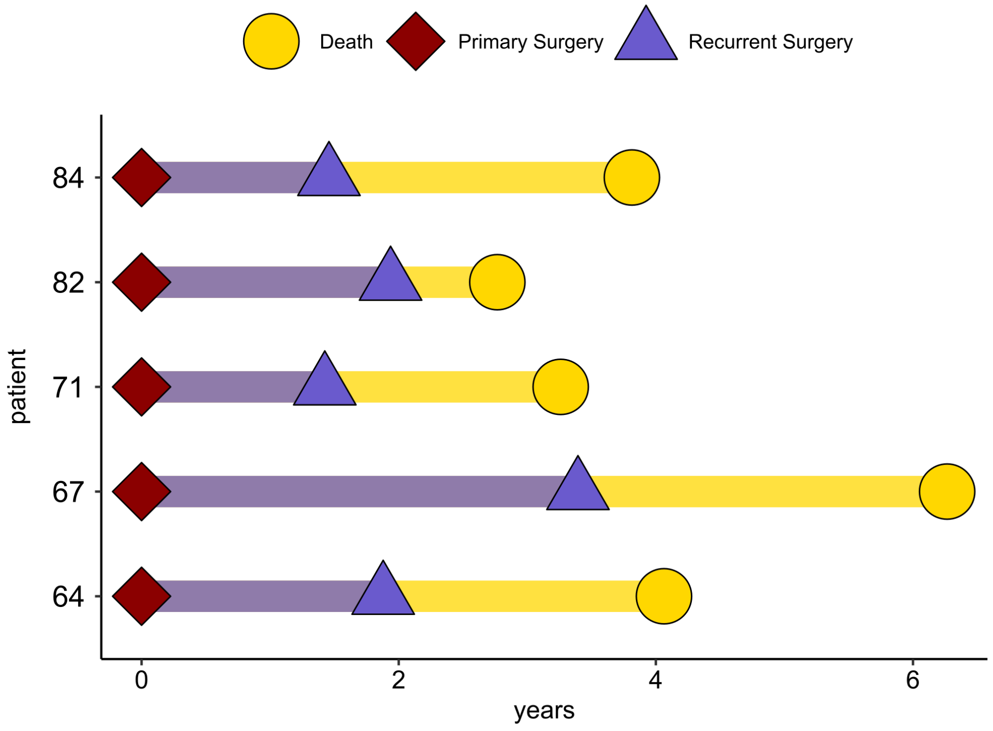

Supplementary Table 2. Imaging mass cytometry (IMC) regions of interest (ROI) analysed in this study.

image id	patient	surgery	ROI	IHC annotation
64Prim_001	64	primary	001	prolif
64Prim_002	64	primary	002	hypoxia
64Prim_003	64	primary	003	immune
64Rec_001	64	recurrent	001	immune
64Rec_002	64	recurrent	002	hypoxia
64Rec_003	64	recurrent	003	prolif
67Prim_001	67	primary	001	immune
67Prim_002	67	primary	002	immune
67Prim_003	67	primary	003	unknown
67Rec_001	67	recurrent	001	prolif
67Rec_002	67	recurrent	002	hypoxia
67Rec_003	67	recurrent	003	unknown
71Prim_001	71	primary	001	hypoxia
71Prim_002	71	primary	002	immune
71Prim_003	71	primary	003	prolif
71Rec_001	71	recurrent	001	prolif
71Rec_002	71	recurrent	002	immune
71Rec_003	71	recurrent	003	hypoxia
82Prim_001	82	primary	001	unknown
82Prim_002	82	primary	002	immune
82Prim_003	82	primary	003	prolif
82Rec_001	82	recurrent	001	prolif
82Rec_002	82	recurrent	002	hypoxia
82Rec_003	82	recurrent	003	immune
84Prim_001	84	primary	001	prolif
84Prim_002	84	primary	002	hypoxia
84Prim_003	84	primary	003	immune
84Rec_001	84	recurrent	001	hypoxia
84Rec_002	84	recurrent	002	immune
84Rec_003	84	recurrent	003	prolif

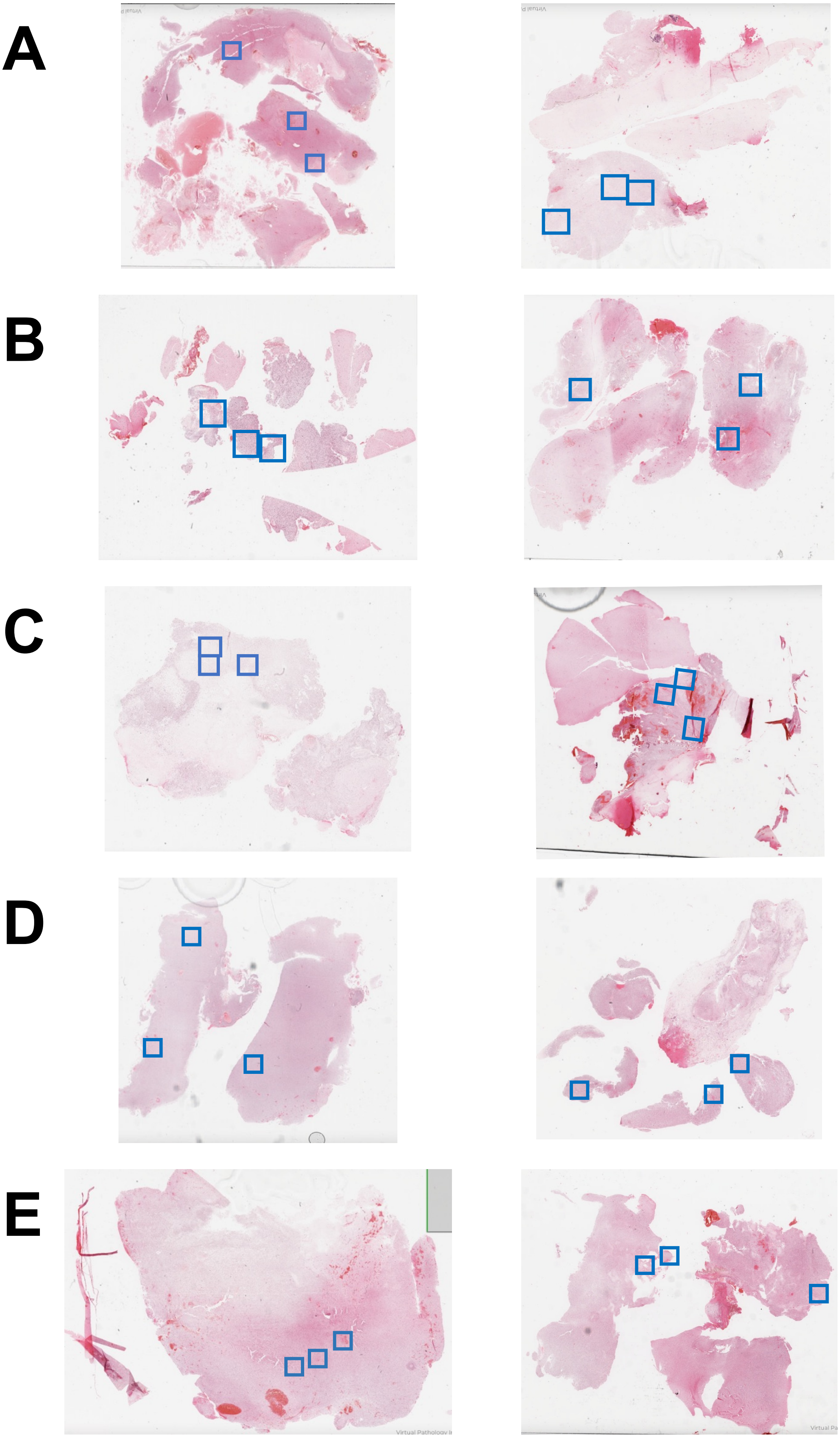

Supplementary Figure 2. H&E stained FFPE sections of the five matched, primary (P) and recurrent (R) IDHwt glioblastoma tumour samples used in the study. The blue demarcate the $1mm^2$ regions of interest (ROI) that underwent imaging mass cytometry (IMC). The FFPE section correspond (from left to right) to the following patient/surgeries: **A)** 64P and 64R; **B)** 67P and 67R; **C)** 71P and 71R; **D)** 82P and 82R; **E)** 84P and 84R.

Supplementary Table 3. Imaging mass cytometry (IMC) marker panel used in this study, including justification of marker/antibody selection. Astrocyte-like (AC-like); bovine serum albumin (BSA); Cytometry by time of flight (CyTOF); Endoplasmic reticulum (ER); Epithelial to mesenchymal transition (EMT); Immunocytochemistry (ICC); Immunohistochemistry (IHC); immunofluorescence (IF); Knock-out (KO); Mesenchymal-like (MES-like); neural progenitor-like (NPC-like); oligodendrocyte progenitor-like (OPC-like); Phosphate buffered saline (PBS); PMID (PubMed Identifier).

marker	cell category	cell type	cell state	location 1	location 2	marker justification (PMIDs)	manufacturer (antibody clone)	antibody justification	antibody concentration (µg/mL)	metal-isotope	control tissue
HOPX	cancer	AC-like		cytoplasm		31327527; 31554641; 32641768	abcam (ab230544)		100	Yb171	Brain, Tonsil
SLC1A3_EAAT1	cancer	AC-like		membrane		31327527; 31554641; 32641768	abcam(ab240235)	BSA and azide free	1000	Gd158	Brain
GFAP	normal	astrocyte		cytoplasm		25726916	abcam (ab218309)	PMID: 34174183	100	Sm149	Brain
CD56	normal	neuron		membrane	extracellular	28791027	biolegend (318345)	PMID: 28369679	100	Dy162	Brain
IBA1	immune	macrophage		cytoplasm		32848611	abcam (ab220815)	PMID: 34174183	200	Eu153	Spleen, Tonsil
NeuN_FOX3	normal	neuron		nucleus		20452351	biolegend (834502)	PMID: 34174183	400	Sm147	Brain
ANXA_A1	cancer	MES-like		cytoplasm	membrane	31327527; 31554641; 32641768	abcam (ab222398)	BSA and azide free		Yb172	Tonsil
ANXA_A2	cancer	MES-like		cytoplasm	membrane	31327527; 31554641; 32641768	rndsystems (mab3928)	In PBS with Trehalose	8-25	Er166	Prostate, Tonsil
CHI3L1	cancer	MES-like		cytoplasm	extracellular	31327527; 31554641; 32641768	abcam (ab255864)	BSA and azide free	250	Sm154	Spleen, Brain
SOD2	cancer	MES-like		mitochondria		31327527; 31554641; 32641768	abcam (ab227846)	BSA and azide free, used in IHC	100	Nd146	Prostate
P2Y12R	immune	microglia		membrane		32848611	abcam (ab274386)	BSA and azide free	1000	Lu175	Brain
TMEM119	immune	microglia		cytoplasm	membrane	32848611	sigmaaldrich (HPA051870)	PMID: 31740814	500-1000	Gd155	Brain, Tonsil
NKP46	immune	NK cell		membrane		31784984	rndsystems (mab1850)	PMID: 36689332	5-25	Nd144	Spleen
BCAN	cancer	NPC-like		extracellular	nucleus	31327527; 31554641; 32641768	thermofisher (MA5-27639)	BSA free, used in ICC	50	Gd160	Brain
DLL3	cancer	NPC-like		membrane		31327527; 31554641; 32641768	abcam (ab255694)	BSA and azide free	100	Nd148	Brain
MOG	normal	oligodendrocyte		membrane		2649509	rndsystems (mab1850)		5-25	Gd157	Brain
OLIG1	cancer	OPC-like		nucleus		31327527; 31554641; 32641768	rndsystems (mab2417)	carrier free, used in IHC	8-25	Yb174	Skin
SCD5	cancer	OPC-like		ER		31327527; 31554641; 32641768	thermofisher (PA5-59963)	used in IHC	50	Tm169	Brain
CD3	immune	T cell		membrane		29768164	fluidigm (3170019D)	PMID: 36689332	75-200	Er170	Spleen, Tonsil
CD8	immune	T cell		membrane		29768164	biolegend (344727)	PMID: 28369679	200	Ho165	Spleen, Tonsil
DNA1	DNA intercalator			nucleus			fluidigm (201192B)	Preconugated to 191Ir		Ir191	
DNA2	DNA intercalator			nucleus			fluidigm (201192B)	Preconugated to 193Ir		Ir193	
CD45	immune			membrane		12414720	fluidigm (91H029152)	Preconugated to 152Sm	300	Sm152	Spleen, Tonsil
CD31	vasculature			membrane		27055047	fluidigm (3151025D)	Preconugated to 151Eu		Eu151	Skin, Tonsil, Prostate
SMA	vasculature			cytoplasm		19929197	rndsystems (mab1420)	used in Cytof	8-25	Dy164	Prostate, Skin, Tonsil
EZH2			transcript respressive	nucleus		23720055	abcam (ab231165)	BSA and azide free	250	Nd145	Tonsil
HIF1A			hypoxia	cytoplasm	nucleus	11606368	thermofisher (700505)	PMID: 32868913	400	Dy161	Bone marrow
JARID2_C_Terminus			active	nucleus		30573669	developed in house			Nd143	Brain
JARID2_N_Terminus			repressed	nucleus		30573669	abcam (ab251123)	BSA free version, validated (by KO)		Yb173	Brain
Ki67			proliferating	nucleus		29322240	fluidigm (3168001B)	Preconugated to 168Er		Er168	Skin, Tonsil
SNAI1			EMT	nucleus	cytoplasm	33806868	rndsystems (af3639)	BSA and azide free, used in IHC	5-15	Tb159	Ubiquitous
SOX2			proliferating	nucleus		30952620	fluidigm (3150019B)	Preconugated to 150Nd		Nd150	Brain, Tonsil
TGFBeta			quiescent	extracellular		30952620	fluidigm (3163010B)	Pre-conjugated to 163Dy		Dy163	Spleen, Bone marrow, Prostate
TNC			quiescent	extracellular		30952620	rndsystems (mab2138)	used in IF	8-25	Gd156	Uterus

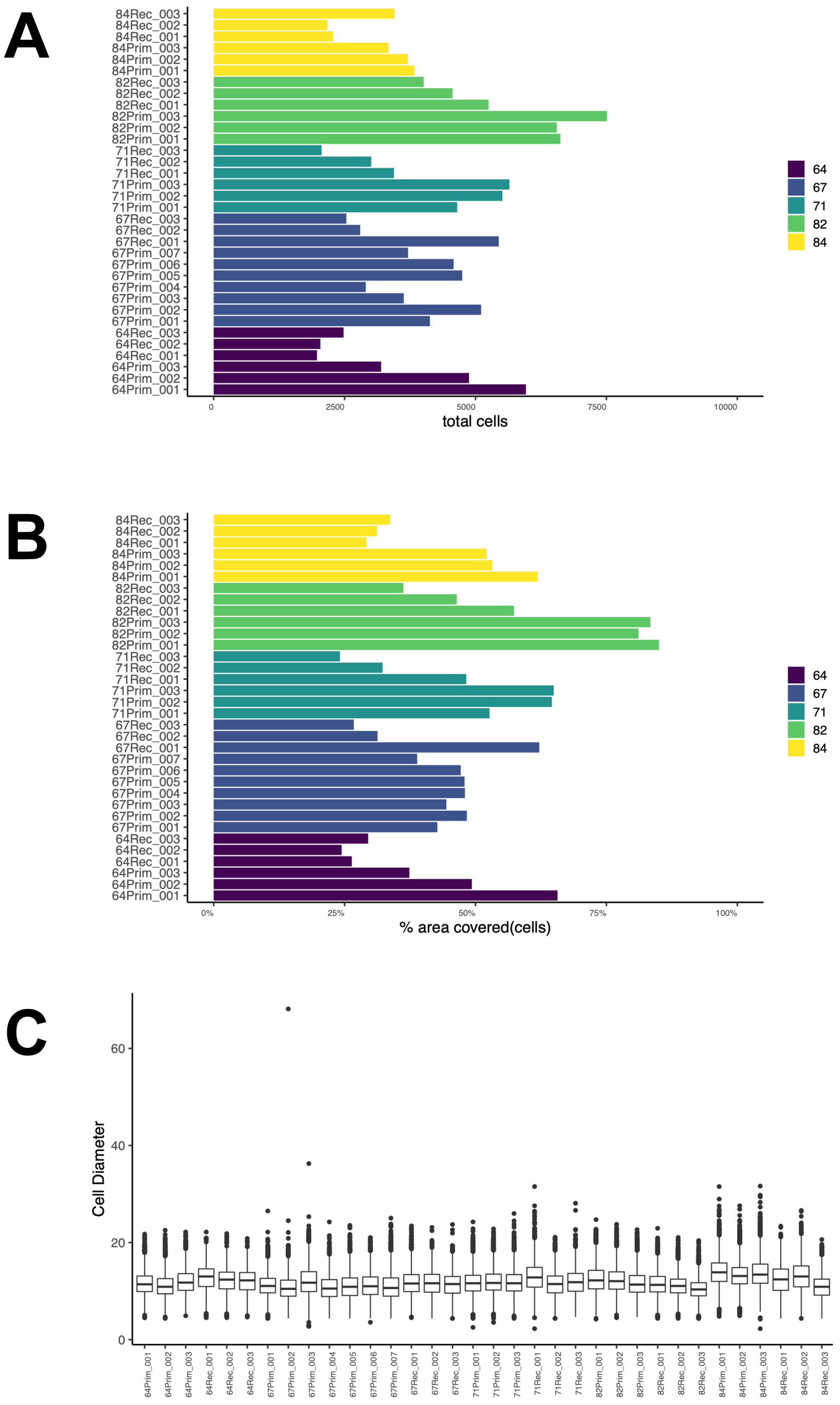

Supplementary Figure 3. Segmented single-cell object metrics. A) The total number of cells present in each region of interest (ROI) colored by patient; **B)** The percentage area covered by the segmented cell objects present across each ROI colored by patient; **C)** The distribution of cell diameters corresponding to the segmented cell objects present in each patient/surgery ROI.

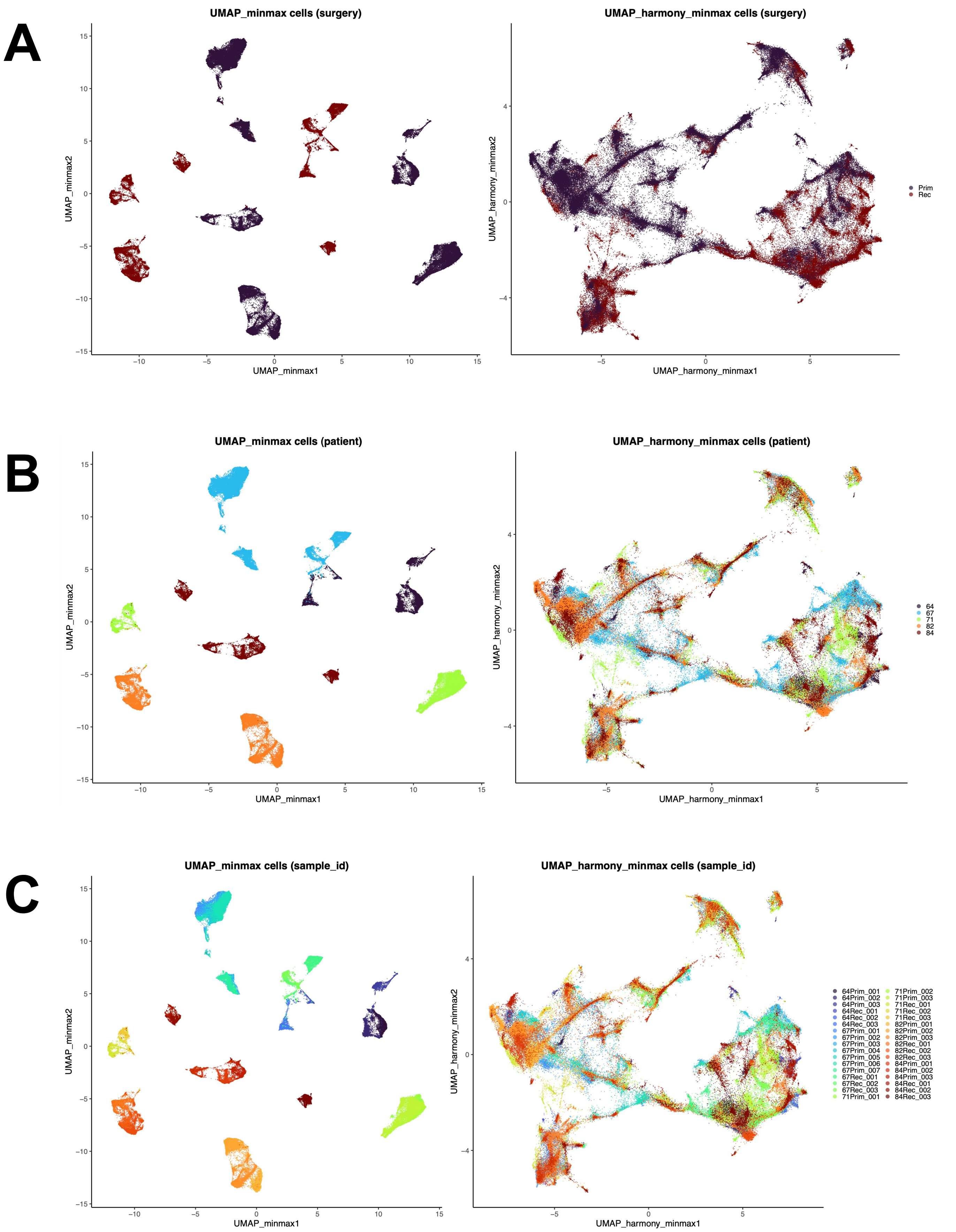

Supplementary Figure 4. UMAP projections of all segmented single-cell objects before (left) and after (right) batch correction of min-max scaled protein marker abundances. The projections are colored according to known sources of sample variation: **A)** surgery type; **B)** patient from which samples were obtained; **C)** patient and surgery-specific regions of interest (ROI).

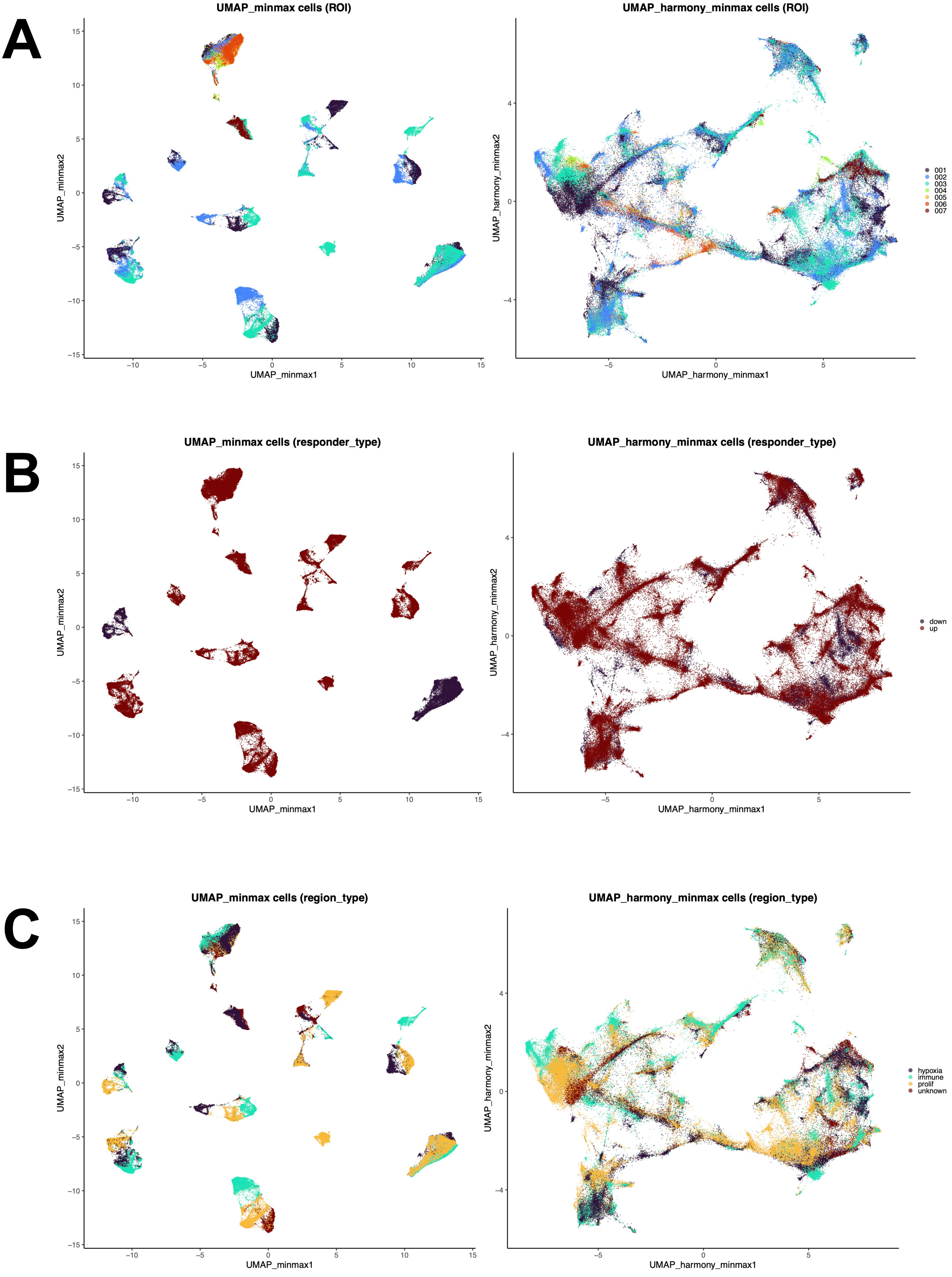

Supplementary Figure 5. UMAP projections of all segmented single-cell objects before (left) and after (right) batch correction of min-max scaled protein marker abundances. The projections are colored according to the following known sources of sample variation: **A)** regions of interest (ROI); **B)** responder types as defined in Tanner et al. *Genome Biology* 25, no. 1 (7 February 2024): 45; **C)** Immunohistochemical annotation of each ROI based on areas which have high levels of hypoxia, proliferation, and immune cells.

Supplementary Table 4. Gating criteria used to annotate cell types and cell states. Statistical significance was assessed using an unpaired Wilcoxon test, with adjusted p-values calculated using the false discovery rate (FDR) method.

Cell type/cell state	Marker(s)	Expression ranks
Endothelial Cell	SMA+ CD31+	>= 15
NK Cell	CD45+ NKP46+	>= 14
T Cell	CD45+ CD3+	>= 11
Macrophage	CD45+ IBA1+	>= 15
Microglia	CD45+ IBA1+ TMEM119+	>= 12
Neuron	NeuN+ NKP46+ CD3+	>= 16
Astrocyte	GFAP+	>= 17
Oligodendrocyte	MOG+	>= 18
Astrocyte-like	SLC1A3_EAAT1+ HOPX+	>= 17
Mesenchymal-like	SOD2+ CHI3L1+ ANEXIN_A2+ ANXA1+	>= 17
Neural progenitor cell-like	DLL3+ BCAN+	>= 15
Oligodendrocyte progenitor-like	SCD5+ OLIG1+	>= 15

Supplementary Table 5. ANOVA results comparing intra-tumour (across patient regions of interest, ROI) and inter-tumor (within patient and surgery sample) heterogeneity of cell categories. The p value significance levels are denoted using the following symbols: **** (p < 0.0001); *** (p < 0.001); ** (p < 0.01); * (p < 0.05); n.s (not significant).

cell category	source of variation	effect size (F statistic)	p value	p significance
Immune	patient & surgery	5.82	7.62E-04	***
Cancer	patient & surgery	9.47	3.33E-05	****
Normal	patient & surgery	10.11	2.11E-05	****
Vasculature	patient & surgery	4.56	3.01E-03	**
Immune	within tumor (ROI)	0.32	7.29E-01	n.s
Cancer	within tumor (ROI)	2.63	9.97E-02	n.s
Normal	within tumor (ROI)	1.23	3.15E-01	n.s
Vasculature	within tumor (ROI)	1.75	2.02E-01	n.s

Supplementary Table 6. Comparison of cell category prevalence between primary and recurrent samples. Statistical significance was assessed using an unpaired Wilcoxon test, with adjusted p-values calculated using the false discovery rate (FDR) method.

cell category	comparison groups	n (per comparison group)	p value	adjusted p value
Immune	Prim vs Rec	15	9.75E-02	9.75E-02
Cancer	Prim vs Rec	15	6.13E-02	8.17E-02
Normal	Prim vs Rec	15	1.13E-04	4.52E-04
Vasculature	Prim vs Rec	15	4.94E-03	9.88E-03

Supplementary Table 7. Changes in cell type prevalence between primary and recurrent samples. Statistical significance was assessed using the unpaired Wilcoxon test, with adjusted p-values calculated using the false discovery rate (FDR) method.

cell type	comparison groups	n (per comparison group)	p value	adjusted p value
T cell	Prim vs Rec	15	5.12E-01	6.83E-01
NK cell	Prim vs Rec	15	2.02E-01	3.72E-01
Macrophage	Prim vs Rec	15	2.17E-01	3.72E-01
Microglia	Prim vs Rec	15	2.50E-01	3.75E-01
AC	Prim vs Rec	15	6.53E-02	1.57E-01
MES	Prim vs Rec	15	9.17E-01	9.17E-01
NPC	Prim vs Rec	15	8.33E-01	9.09E-01
OPC	Prim vs Rec	15	8.14E-01	9.09E-01
Neuron	Prim vs Rec	15	4.17E-03	1.98E-02
Astrocyte	Prim vs Rec	15	3.02E-03	1.98E-02
Oligodendrocyte	Prim vs Rec	15	2.25E-02	6.75E-02
Endothelial	Prim vs Rec	15	4.94E-03	1.98E-02

Supplementary Table 8. Comparison of Shannon's entropy (H) between primary and recurrent samples, quantifying intra-tumour cellular heterogeneity. Statistical significance was assessed using the unpaired, Wilcoxon test, with adjusted p-values calculated using the false discovery rate (FDR) method.

patient(s)	comparision group	n (per comparison group)	p value	adjusted p value
All	Prim vs Rec	150	3.93E-03	3.93E-03
64	Prim vs Rec	30	1.43E-04	3.58E-04
67	Prim vs Rec	30	2.63E-02	2.63E-02
71	Prim vs Rec	30	1.69E-17	8.45E-17
82	Prim vs Rec	30	1.52E-03	1.90E-03
84	Prim vs Rec	30	4.26E-04	7.10E-04

Supplementary Table 9. Changes in cellular states (hypoxia and Epithelial to mesenchymal transition - EMT) across neoplastic GBM cancer cell types in primary and recurrent samples. Statistical significance was assessed using the unpaired, Wilcoxon test, with adjusted p-values calculated using the false discovery rate (FDR) method. The p value significance levels are denoted using the following symbols: **** ($p < 0.0001$); *** ($p < 0.001$); ** ($p < 0.01$); * ($p < 0.05$); n.s (not significant).

cancer cell type	cellular state	comparison groups	n cells (primary)	n cells (recurrent)	p value	adjusted p value	p significance
AC	hypoxia	Prim vs Rec	5522	686	4.98E-115	9.96E-115	****
MES	hypoxia	Prim vs Rec	9141	3119	9.62E-125	3.85E-124	****
NPC	hypoxia	Prim vs Rec	1664	613	5.49E-70	7.32E-70	****
OPC	hypoxia	Prim vs Rec	1743	616	7.41E-01	7.41E-01	n.s
AC	EMT	Prim vs Rec	5522	686	5.29E-161	1.06E-160	****
MES	EMT	Prim vs Rec	9141	3119	1.03E-07	1.37E-07	****
NPC	EMT	Prim vs Rec	1664	613	2.32E-183	9.28E-183	****
OPC	EMT	Prim vs Rec	1743	616	4.40E-03	4.40E-03	**

Supplementary Table 10. Patient-specific, observed, cell-cell interactions compared to a null model of spatial randomness. The results are separated by patient and surgery type (primary or recurrent). Statistical significance and direction is determined using a permutation test, with p-values indicating interactions more or less likely than random: 1 (significant positive interactions); -1 (significant avoidance interactions); 0 (neutral and/or non- statistically significant interactions).

row	patient	surgery	from cell type	to cell type	observed count	permutations (greater than)	permutations (less than)	p value	interaction significance/direction
1	64	primary	AC	AC	2.96	9.99E-04	1.00E+00	9.99E-04	1
2	64	primary	AC	Astrocyte	1.05	2.90E-02	9.72E-01	2.90E-02	0
3	64	primary	AC	Endothelial	1.42	9.99E-04	1.00E+00	9.99E-04	1
4	64	primary	AC	MES	1.62	1.00E+00	9.99E-04	9.99E-04	-1
5	64	primary	AC	Macrophage	1.49	9.99E-04	1.00E+00	9.99E-04	1
6	64	primary	AC	Microglia	1.24	9.16E-01	8.49E-02	8.49E-02	0
7	64	primary	AC	NK cell	1.16	9.99E-04	1.00E+00	9.99E-04	1
8	64	primary	AC	NPC	1.00	1.00E+00	9.91E-01	9.91E-01	0
9	64	primary	AC	Neuron	1.06	1.10E-02	9.92E-01	1.10E-02	0
10	64	primary	AC	OPC	1.00	9.57E-01	9.99E-01	9.57E-01	0
11	64	primary	AC	Oligodendrocyte	1.33	1.31E-01	8.70E-01	1.31E-01	0
12	64	primary	AC	T cell	1.31	2.00E-03	9.99E-01	2.00E-03	1
13	64	primary	Astrocyte	AC	2.24	9.99E-04	1.00E+00	9.99E-04	1
14	64	primary	Astrocyte	Astrocyte	1.08	1.80E-02	9.83E-01	1.80E-02	0
15	64	primary	Astrocyte	Endothelial	1.53	1.70E-02	9.84E-01	1.70E-02	0
16	64	primary	Astrocyte	MES	2.60	9.99E-04	1.00E+00	9.99E-04	1
17	64	primary	Astrocyte	Macrophage	1.58	2.90E-02	9.73E-01	2.90E-02	0
18	64	primary	Astrocyte	Microglia	1.14	9.21E-01	8.19E-02	8.19E-02	0
19	64	primary	Astrocyte	NK cell	1.00	9.98E-01	7.54E-01	7.54E-01	0
20	64	primary	Astrocyte	NPC	1.00	1.53E-01	1.00E+00	1.53E-01	0
21	64	primary	Astrocyte	Neuron	1.00	4.21E-01	1.00E+00	4.21E-01	0
22	64	primary	Astrocyte	OPC	0.00	1.00E+00	9.34E-01	9.34E-01	0
23	64	primary	Astrocyte	Oligodendrocyte	1.19	9.00E-01	1.06E-01	1.06E-01	0
24	64	primary	Astrocyte	T cell	1.10	9.74E-01	2.80E-02	2.80E-02	0
25	64	primary	Endothelial	AC	1.88	9.99E-04	1.00E+00	9.99E-04	1
26	64	primary	Endothelial	Astrocyte	1.09	9.99E-03	9.91E-01	9.99E-03	1
27	64	primary	Endothelial	Endothelial	2.06	9.99E-04	1.00E+00	9.99E-04	1
28	64	primary	Endothelial	MES	1.90	9.99E-04	1.00E+00	9.99E-04	1
29	64	primary	Endothelial	Macrophage	1.44	2.20E-02	9.79E-01	2.20E-02	0
30	64	primary	Endothelial	Microglia	1.35	9.99E-04	1.00E+00	9.99E-04	1
31	64	primary	Endothelial	NK cell	1.24	9.99E-04	1.00E+00	9.99E-04	1
32	64	primary	Endothelial	NPC	1.00	9.54E-01	9.98E-01	9.54E-01	0
33	64	primary	Endothelial	Neuron	1.00	1.00E+00	9.71E-01	9.71E-01	0
34	64	primary	Endothelial	OPC	1.00	7.01E-01	1.00E+00	7.01E-01	0
35	64	primary	Endothelial	Oligodendrocyte	1.39	9.99E-04	1.00E+00	9.99E-04	1
36	64	primary	Endothelial	T cell	1.33	9.99E-04	1.00E+00	9.99E-04	1
37	64	primary	MES	AC	1.77	4.80E-02	9.53E-01	4.80E-02	0
38	64	primary	MES	Astrocyte	1.07	5.00E-03	9.96E-01	5.00E-03	1
39	64	primary	MES	Endothelial	1.38	9.99E-04	1.00E+00	9.99E-04	1
40	64	primary	MES	MES	3.59	9.99E-04	1.00E+00	9.99E-04	1
41	64	primary	MES	Macrophage	1.30	1.00E+00	9.99E-04	9.99E-04	-1
42	64	primary	MES	Microglia	1.28	7.79E-02	9.23E-01	7.79E-02	0
43	64	primary	MES	NK cell	1.25	9.99E-04	1.00E+00	9.99E-04	1
44	64	primary	MES	NPC	1.00	9.98E-01	9.99E-01	9.98E-01	0
45	64	primary	MES	Neuron	1.00	1.00E+00	9.46E-01	9.46E-01	0
46	64	primary	MES	OPC	0.00	1.00E+00	5.00E-02	5.00E-02	0
47	64	primary	MES	Oligodendrocyte	1.59	9.99E-04	1.00E+00	9.99E-04	1
48	64	primary	MES	T cell	1.33	9.99E-04	1.00E+00	9.99E-04	1
49	64	primary	Macrophage	AC	2.60	9.99E-04	1.00E+00	9.99E-04	1
50	64	primary	Macrophage	Astrocyte	1.12	9.99E-04	1.00E+00	9.99E-04	1
51	64	primary	Macrophage	Endothelial	1.50	9.99E-04	1.00E+00	9.99E-04	1
52	64	primary	Macrophage	MES	2.42	9.99E-04	1.00E+00	9.99E-04	1
53	64	primary	Macrophage	Macrophage	1.59	9.99E-04	1.00E+00	9.99E-04	1
54	64	primary	Macrophage	Microglia	1.48	9.99E-04	1.00E+00	9.99E-04	1
55	64	primary	Macrophage	NK cell	1.19	9.99E-04	1.00E+00	9.99E-04	1
56	64	primary	Macrophage	NPC	1.00	9.86E-01	9.97E-01	9.86E-01	0
57	64	primary	Macrophage	Neuron	1.00	1.00E+00	9.66E-01	9.66E-01	0
58	64	primary	Macrophage	OPC	1.00	8.16E-01	9.99E-01	8.16E-01	0
59	64	primary	Macrophage	Oligodendrocyte	1.46	9.99E-04	1.00E+00	9.99E-04	1
60	64	primary	Macrophage	T cell	1.32	2.00E-03	9.99E-01	2.00E-03	1
61	64	primary	Microglia	AC	2.40	9.99E-04	1.00E+00	9.99E-04	1
62	64	primary	Microglia	Astrocyte	1.09	7.99E-03	9.93E-01	7.99E-03	1
63	64	primary	Microglia	Endothelial	1.52	9.99E-04	1.00E+00	9.99E-04	1
64	64	primary	Microglia	MES	1.94	9.99E-04	1.00E+00	9.99E-04	1
65	64	primary	Microglia	Macrophage	1.52	9.99E-04	1.00E+00	9.99E-04	1
66	64	primary	Microglia	Microglia	1.72	9.99E-04	1.00E+00	9.99E-04	1
67	64	primary	Microglia	NK cell	1.44	9.99E-04	1.00E+00	9.99E-04	1
68	64	primary	Microglia	NPC	1.00	9.43E-01	9.98E-01	9.43E-01	0
69	64	primary	Microglia	Neuron	0.00	1.00E+00	9.99E-04	9.99E-04	-1
70	64	primary	Microglia	OPC	0.00	1.00E+00	3.25E-01	3.25E-01	0
71	64	primary	Microglia	Oligodendrocyte	1.50	9.99E-04	1.00E+00	9.99E-04	1
72	64	primary	Microglia	T cell	1.49	9.99E-04	1.00E+00	9.99E-04	1
73	64	primary	NK cell	AC	2.17	9.99E-04	1.00E+00	9.99E-04	1
74	64	primary	NK cell	Astrocyte	1.00	9.98E-01	8.84E-01	8.84E-01	0
75	64	primary	NK cell	Endothelial	1.49	2.00E-03	9.99E-01	2.00E-03	1
76	64	primary	NK cell	MES	1.38	1.00E+00	9.99E-04	9.99E-04	-1
77	64	primary	NK cell	Macrophage	1.46	1.05E-01	8.96E-01	1.05E-01	0
78	64	primary	NK cell	Microglia	1.79	9.99E-04	1.00E+00	9.99E-04	1
79	64	primary	NK cell	NK cell	1.78	9.99E-04	1.00E+00	9.99E-04	1
80	64	primary	NK cell	NPC	0.00	1.00E+00	5.77E-01	5.77E-01	0
81	64	primary	NK cell	Neuron	1.00	7.89E-01	9.98E-01	7.89E-01	0
82	64	primary	NK cell	OPC	1.00	1.97E-01	1.00E+00	1.97E-01	0
83	64	primary	NK cell	Oligodendrocyte	1.06	1.00E+00	9.99E-04	9.99E-04	-1
84	64	primary	NK cell	T cell	1.70	9.99E-04	1.00E+00	9.99E-04	1
85	64	primary	NPC	AC	2.33	1.03E-01	9.15E-01	1.03E-01	0
86	64	primary	NPC	Astrocyte	1.00	1.53E-01	9.94E-01	1.53E-01	0
87	64	primary	NPC	Endothelial	2.00	1.13E-01	9.81E-01	1.13E-01	0
88	64	primary	NPC	MES	1.50	7.03E-01	4.05E-01	4.05E-01	0
89	64	primary	NPC	Macrophage	1.00	9.86E-01	3.71E-01	3.71E-01	0
90	64	primary	NPC	Microglia	1.50	2.40E-01	8.69E-01	2.40E-01	0
91	64	primary	NPC	NK cell	0.00	1.00E+00	5.77E-01	5.77E-01	0
92	64	primary	NPC	NPC	0.00	1.00E+00	9.99E-01	9.99E-01	0
93	64	primary	NPC	Neuron	0.00	1.00E+00	9.67E-01	9.67E-01	0
94	64	primary	NPC	OPC	0.00	1.00E+00	9.96E-01	9.96E-01	0
95	64	primary	NPC	Oligodendrocyte	0.00	1.00E+00	4.30E-02	4.30E-02	0
96	64	primary	NPC	T cell	1.33	3.73E-01	7.19E-01	3.73E-01	0
97	64	primary	Neuron	AC	2.83	2.00E-03	9.99E-01	2.00E-03	1
98	64	primary	Neuron	Astrocyte	1.00	4.21E-01	9.90E-01	4.21E-01	0
99	64	primary	Neuron	Endothelial	1.50	1.63E-01	8.96E-01	1.63E-01	0
100	64	primary	Neuron	MES	4.50	9.99E-04	1.00E+00	9.99E-04	1

row	patient	surgery	from cell type	to cell tye	observed count	permutations (greater than)	permutations (less than)	p value	interaction significance/direction
101	64	primary	Neuron	Macrophage	1.33	6.02E-01	4.83E-01	4.83E-01	0
102	64	primary	Neuron	Microglia	0.00	1.00E+00	9.99E-04	9.99E-04	-1
103	64	primary	Neuron	NK cell	1.00	7.89E-01	9.36E-01	7.89E-01	0
104	64	primary	Neuron	NPC	0.00	1.00E+00	9.67E-01	9.67E-01	0
105	64	primary	Neuron	Neuron	0.00	1.00E+00	9.55E-01	9.55E-01	0
106	64	primary	Neuron	OPC	0.00	1.00E+00	9.85E-01	9.85E-01	0
107	64	primary	Neuron	Oligodendrocyte	1.50	2.30E-01	8.49E-01	2.30E-01	0
108	64	primary	Neuron	T cell	1.00	9.99E-01	2.35E-01	2.35E-01	0
109	64	primary	OPC	AC	4.00	1.80E-02	9.97E-01	1.80E-02	0
110	64	primary	OPC	Astrocyte	0.00	1.00E+00	9.34E-01	9.34E-01	0
111	64	primary	OPC	Endothelial	1.00	7.01E-01	7.93E-01	7.01E-01	0
112	64	primary	OPC	MES	0.00	1.00E+00	5.00E-02	5.00E-02	0
113	64	primary	OPC	Macrophage	2.00	1.99E-01	9.60E-01	1.99E-01	0
114	64	primary	OPC	Microglia	0.00	1.00E+00	3.25E-01	3.25E-01	0
115	64	primary	OPC	NK cell	1.00	1.97E-01	9.89E-01	1.97E-01	0
116	64	primary	OPC	NPC	0.00	1.00E+00	9.96E-01	9.96E-01	0
117	64	primary	OPC	Neuron	0.00	1.00E+00	9.85E-01	9.85E-01	0
118	64	primary	OPC	OPC	0.00	1.00E+00	1.00E+00	1.00E+00	0
119	64	primary	OPC	Oligodendrocyte	1.00	7.40E-01	7.57E-01	7.40E-01	0
120	64	primary	OPC	T cell	0.00	1.00E+00	3.13E-01	3.13E-01	0
121	64	primary	Oligodendrocyte	AC	2.31	9.99E-04	1.00E+00	9.99E-04	1
122	64	primary	Oligodendrocyte	Astrocyte	1.00	1.00E+00	5.58E-01	5.58E-01	0
123	64	primary	Oligodendrocyte	Endothelial	1.48	9.99E-04	1.00E+00	9.99E-04	1
124	64	primary	Oligodendrocyte	MES	1.84	9.99E-04	1.00E+00	9.99E-04	1
125	64	primary	Oligodendrocyte	Macrophage	1.38	5.84E-01	4.18E-01	4.18E-01	0
126	64	primary	Oligodendrocyte	Microglia	1.32	2.00E-03	9.99E-01	2.00E-03	1
127	64	primary	Oligodendrocyte	NK cell	1.09	2.50E-02	9.79E-01	2.50E-02	0
128	64	primary	Oligodendrocyte	NPC	0.00	1.00E+00	4.30E-02	4.30E-02	0
129	64	primary	Oligodendrocyte	Neuron	1.00	1.00E+00	9.70E-01	9.70E-01	0
130	64	primary	Oligodendrocyte	OPC	1.00	7.40E-01	1.00E+00	7.40E-01	0
131	64	primary	Oligodendrocyte	Oligodendrocyte	1.86	9.99E-04	1.00E+00	9.99E-04	1
132	64	primary	Oligodendrocyte	T cell	1.56	9.99E-04	1.00E+00	9.99E-04	1
133	64	primary	T cell	AC	2.13	9.99E-04	1.00E+00	9.99E-04	1
134	64	primary	T cell	Astrocyte	1.00	1.00E+00	5.98E-01	5.98E-01	0
135	64	primary	T cell	Endothelial	1.48	9.99E-04	1.00E+00	9.99E-04	1
136	64	primary	T cell	MES	2.04	9.99E-04	1.00E+00	9.99E-04	1
137	64	primary	T cell	Macrophage	1.37	6.53E-01	3.48E-01	3.48E-01	0
138	64	primary	T cell	Microglia	1.36	9.99E-04	1.00E+00	9.99E-04	1
139	64	primary	T cell	NK cell	1.48	9.99E-04	1.00E+00	9.99E-04	1
140	64	primary	T cell	NPC	1.00	9.30E-01	9.97E-01	9.30E-01	0
141	64	primary	T cell	Neuron	1.00	9.99E-01	9.71E-01	9.71E-01	0
142	64	primary	T cell	OPC	0.00	1.00E+00	3.13E-01	3.13E-01	0
143	64	primary	T cell	Oligodendrocyte	1.78	9.99E-04	1.00E+00	9.99E-04	1
144	64	primary	T cell	T cell	1.81	9.99E-04	1.00E+00	9.99E-04	1
145	64	recurrent	AC	AC					
146	64	recurrent	AC	Astrocyte					
147	64	recurrent	AC	Endothelial					
148	64	recurrent	AC	MES					
149	64	recurrent	AC	Macrophage					
150	64	recurrent	AC	Microglia					
151	64	recurrent	AC	NK cell					
152	64	recurrent	AC	NPC					
153	64	recurrent	AC	Neuron					
154	64	recurrent	AC	OPC					
155	64	recurrent	AC	Oligodendrocyte					
156	64	recurrent	AC	T cell					
157	64	recurrent	Astrocyte	AC					
158	64	recurrent	Astrocyte	Astrocyte	2.99	9.99E-04	1.00E+00	9.99E-04	1
159	64	recurrent	Astrocyte	Endothelial	1.15	4.00E-03	9.97E-01	4.00E-03	1
160	64	recurrent	Astrocyte	MES	1.24	3.00E-03	9.98E-01	3.00E-03	1
161	64	recurrent	Astrocyte	Macrophage	1.13	9.65E-01	3.60E-02	3.60E-02	0
162	64	recurrent	Astrocyte	Microglia	1.16	1.50E-02	9.87E-01	1.50E-02	0
163	64	recurrent	Astrocyte	NK cell	1.17	1.29E-01	8.72E-01	1.29E-01	0
164	64	recurrent	Astrocyte	NPC	1.34	9.99E-04	1.00E+00	9.99E-04	1
165	64	recurrent	Astrocyte	Neuron	1.20	9.99E-04	1.00E+00	9.99E-04	1
166	64	recurrent	Astrocyte	OPC	0.00	1.00E+00	1.42E-01	1.42E-01	0
167	64	recurrent	Astrocyte	Oligodendrocyte	1.91	9.99E-04	1.00E+00	9.99E-04	1
168	64	recurrent	Astrocyte	T cell	1.11	9.50E-01	5.09E-02	5.09E-02	0
169	64	recurrent	Endothelial	AC					
170	64	recurrent	Endothelial	Astrocyte	2.46	9.99E-04	1.00E+00	9.99E-04	1
171	64	recurrent	Endothelial	Endothelial	1.88	9.99E-04	1.00E+00	9.99E-04	1
172	64	recurrent	Endothelial	MES	1.17	5.55E-01	4.65E-01	4.65E-01	0
173	64	recurrent	Endothelial	Macrophage	1.28	3.20E-02	9.69E-01	3.20E-02	0
174	64	recurrent	Endothelial	Microglia	1.22	5.09E-02	9.50E-01	5.09E-02	0
175	64	recurrent	Endothelial	NK cell	1.83	9.99E-04	1.00E+00	9.99E-04	1
176	64	recurrent	Endothelial	NPC	1.30	2.40E-02	9.78E-01	2.40E-02	0
177	64	recurrent	Endothelial	Neuron	1.16	1.28E-01	8.73E-01	1.28E-01	0
178	64	recurrent	Endothelial	OPC	0.00	1.00E+00	8.17E-01	8.17E-01	0
179	64	recurrent	Endothelial	Oligodendrocyte	1.35	1.00E+00	9.99E-04	9.99E-04	-1
180	64	recurrent	Endothelial	T cell	1.05	9.50E-01	5.29E-02	5.29E-02	0
181	64	recurrent	MES	AC					
182	64	recurrent	MES	Astrocyte	1.85	9.93E-01	7.99E-03	7.99E-03	-1
183	64	recurrent	MES	Endothelial	1.17	3.50E-02	9.73E-01	3.50E-02	0
184	64	recurrent	MES	MES	1.70	9.99E-04	1.00E+00	9.99E-04	1
185	64	recurrent	MES	Macrophage	1.39	9.99E-04	1.00E+00	9.99E-04	1
186	64	recurrent	MES	Microglia	1.18	3.50E-02	9.67E-01	3.50E-02	0
187	64	recurrent	MES	NK cell	1.13	5.86E-01	4.19E-01	4.19E-01	0
188	64	recurrent	MES	NPC	1.43	9.99E-04	1.00E+00	9.99E-04	1
189	64	recurrent	MES	Neuron	1.00	1.00E+00	5.00E-03	5.00E-03	-1
190	64	recurrent	MES	OPC	1.00	3.16E-01	1.00E+00	3.16E-01	0
191	64	recurrent	MES	Oligodendrocyte	3.02	9.99E-04	1.00E+00	9.99E-04	1
192	64	recurrent	MES	T cell	1.17	2.43E-01	7.61E-01	2.43E-01	0
193	64	recurrent	Macrophage	AC					
194	64	recurrent	Macrophage	Astrocyte	1.82	9.95E-01	5.99E-03	5.99E-03	-1
195	64	recurrent	Macrophage	Endothelial	1.40	9.99E-04	1.00E+00	9.99E-04	1
196	64	recurrent	Macrophage	MES	1.44	9.99E-04	1.00E+00	9.99E-04	1
197	64	recurrent	Macrophage	Macrophage	1.50	9.99E-04	1.00E+00	9.99E-04	1
198	64	recurrent	Macrophage	Microglia	1.25	2.00E-03	9.99E-01	2.00E-03	1
199	64	recurrent	Macrophage	NK cell	1.47	9.99E-04	1.00E+00	9.99E-04	1
200	64	recurrent	Macrophage	NPC	1.35	2.00E-03	9.99E-01	2.00E-03	1
201	64	recurrent	Macrophage	Neuron	1.23	3.00E-03	9.98E-01	3.00E-03	1
202	64	recurrent	Macrophage	OPC	1.00	3.08E-01	1.00E+00	3.08E-01	0
203	64	recurrent	Macrophage	Oligodendrocyte	2.86	9.99E-04	1.00E+00	9.99E-04	1
204	64	recurrent	Macrophage	T cell	1.20	8.29E-02	9.18E-01	8.29E-02	0
205	64	recurrent	Microglia	AC					
206	64	recurrent	Microglia	Astrocyte	2.53	9.99E-04	1.00E+00	9.99E-04	1
207	64	recurrent	Microglia	Endothelial	1.36	9.99E-04	1.00E+00	9.99E-04	1
208	64	recurrent	Microglia	MES	1.40	9.99E-04	1.00E+00	9.99E-04	1
209	64	recurrent	Microglia	Macrophage	1.53	9.99E-04	1.00E+00	9.99E-04	1
210	64	recurrent	Microglia	Microglia	1.24	5.09E-02	9.51E-01	5.09E-02	0

row	patient	surgery	from cell type	to cell tye	observed count	permutations (greater than)	permutations (less than)	p value	interaction significance/direction
211	64	recurrent	Microglia	NK cell	1.33	2.00E-03	9.99E-01	2.00E-03	1
212	64	recurrent	Microglia	NPC	1.35	2.00E-03	9.99E-01	2.00E-03	1
213	64	recurrent	Microglia	Neuron	1.00	1.00E+00	4.90E-02	4.90E-02	0
214	64	recurrent	Microglia	OPC	0.00	1.00E+00	7.61E-01	7.61E-01	0
215	64	recurrent	Microglia	Oligodendrocyte	2.37	9.99E-04	1.00E+00	9.99E-04	1
216	64	recurrent	Microglia	T cell	1.15	4.23E-01	5.87E-01	4.23E-01	0
217	64	recurrent	NK cell	AC					
218	64	recurrent	NK cell	Astrocyte	2.56	9.99E-04	1.00E+00	9.99E-04	1
219	64	recurrent	NK cell	Endothelial	1.94	9.99E-04	1.00E+00	9.99E-04	1
220	64	recurrent	NK cell	MES	1.31	6.99E-03	9.95E-01	6.99E-03	1
221	64	recurrent	NK cell	Macrophage	1.49	9.99E-04	1.00E+00	9.99E-04	1
222	64	recurrent	NK cell	Microglia	1.26	2.00E-03	9.99E-01	2.00E-03	1
223	64	recurrent	NK cell	NK cell	1.81	9.99E-04	1.00E+00	9.99E-04	1
224	64	recurrent	NK cell	NPC	1.35	9.99E-04	1.00E+00	9.99E-04	1
225	64	recurrent	NK cell	Neuron	1.11	3.01E-01	7.20E-01	3.01E-01	0
226	64	recurrent	NK cell	OPC	0.00	1.00E+00	7.13E-01	7.13E-01	0
227	64	recurrent	NK cell	Oligodendrocyte	2.15	9.99E-04	1.00E+00	9.99E-04	1
228	64	recurrent	NK cell	T cell	1.20	8.49E-02	9.16E-01	8.49E-02	0
229	64	recurrent	NPC	AC					
230	64	recurrent	NPC	Astrocyte	2.69	9.99E-04	1.00E+00	9.99E-04	1
231	64	recurrent	NPC	Endothelial	1.17	4.40E-02	9.60E-01	4.40E-02	0
232	64	recurrent	NPC	MES	1.18	4.55E-01	5.54E-01	4.55E-01	0
233	64	recurrent	NPC	Macrophage	1.10	9.47E-01	5.59E-02	5.59E-02	0
234	64	recurrent	NPC	Microglia	1.11	4.76E-01	5.30E-01	4.76E-01	0
235	64	recurrent	NPC	NK cell	1.15	4.43E-01	5.64E-01	4.43E-01	0
236	64	recurrent	NPC	NPC	1.42	9.99E-04	1.00E+00	9.99E-04	1
237	64	recurrent	NPC	Neuron	1.23	4.00E-03	9.97E-01	4.00E-03	1
238	64	recurrent	NPC	OPC	0.00	1.00E+00	7.06E-01	7.06E-01	0
239	64	recurrent	NPC	Oligodendrocyte	1.11	1.00E+00	9.99E-04	9.99E-04	-1
240	64	recurrent	NPC	T cell	1.04	9.98E-01	3.00E-03	3.00E-03	-1
241	64	recurrent	Neuron	AC					
242	64	recurrent	Neuron	Astrocyte	2.70	9.99E-04	1.00E+00	9.99E-04	1
243	64	recurrent	Neuron	Endothelial	1.10	3.59E-01	6.62E-01	3.59E-01	0
244	64	recurrent	Neuron	MES	1.30	3.20E-02	9.71E-01	3.20E-02	0
245	64	recurrent	Neuron	Macrophage	1.07	9.59E-01	4.50E-02	4.50E-02	0
246	64	recurrent	Neuron	Microglia	1.05	8.64E-01	1.42E-01	1.42E-01	0
247	64	recurrent	Neuron	NK cell	1.11	7.00E-01	3.09E-01	3.09E-01	0
248	64	recurrent	Neuron	NPC	1.52	9.99E-04	1.00E+00	9.99E-04	1
249	64	recurrent	Neuron	Neuron	1.28	1.40E-02	9.87E-01	1.40E-02	0
250	64	recurrent	Neuron	OPC	0.00	1.00E+00	8.22E-01	8.22E-01	0
251	64	recurrent	Neuron	Oligodendrocyte	1.21	1.00E+00	9.99E-04	9.99E-04	-1
252	64	recurrent	Neuron	T cell	1.10	7.54E-01	2.52E-01	2.52E-01	0
253	64	recurrent	OPC	AC					
254	64	recurrent	OPC	Astrocyte	0.00	1.00E+00	1.42E-01	1.42E-01	0
255	64	recurrent	OPC	Endothelial	0.00	1.00E+00	8.17E-01	8.17E-01	0
256	64	recurrent	OPC	MES	1.00	3.16E-01	9.49E-01	3.16E-01	0
257	64	recurrent	OPC	Macrophage	1.00	3.08E-01	9.60E-01	3.08E-01	0
258	64	recurrent	OPC	Microglia	0.00	1.00E+00	7.61E-01	7.61E-01	0
259	64	recurrent	OPC	NK cell	0.00	1.00E+00	7.13E-01	7.13E-01	0
260	64	recurrent	OPC	NPC	0.00	1.00E+00	7.06E-01	7.06E-01	0
261	64	recurrent	OPC	Neuron	0.00	1.00E+00	8.22E-01	8.22E-01	0
262	64	recurrent	OPC	OPC	0.00	1.00E+00	1.00E+00	1.00E+00	0
263	64	recurrent	OPC	Oligodendrocyte	5.00	5.00E-03	9.99E-01	5.00E-03	1
264	64	recurrent	OPC	T cell	0.00	1.00E+00	7.29E-01	7.29E-01	0
265	64	recurrent	Oligodendrocyte	AC					
266	64	recurrent	Oligodendrocyte	Astrocyte	1.36	1.00E+00	9.99E-04	9.99E-04	-1
267	64	recurrent	Oligodendrocyte	Endothelial	1.41	9.99E-04	1.00E+00	9.99E-04	1
268	64	recurrent	Oligodendrocyte	MES	1.46	9.99E-04	1.00E+00	9.99E-04	1
269	64	recurrent	Oligodendrocyte	Macrophage	1.40	9.99E-04	1.00E+00	9.99E-04	1
270	64	recurrent	Oligodendrocyte	Microglia	1.17	8.99E-03	9.92E-01	8.99E-03	1
271	64	recurrent	Oligodendrocyte	NK cell	1.20	1.10E-02	9.90E-01	1.10E-02	0
272	64	recurrent	Oligodendrocyte	NPC	1.39	9.99E-04	1.00E+00	9.99E-04	1
273	64	recurrent	Oligodendrocyte	Neuron	1.12	1.34E-01	8.67E-01	1.34E-01	0
274	64	recurrent	Oligodendrocyte	OPC	1.00	7.66E-01	1.00E+00	7.66E-01	0
275	64	recurrent	Oligodendrocyte	Oligodendrocyte	3.31	9.99E-04	1.00E+00	9.99E-04	1
276	64	recurrent	Oligodendrocyte	T cell	1.21	4.00E-03	9.97E-01	4.00E-03	1
277	64	recurrent	T cell	AC					
278	64	recurrent	T cell	Astrocyte	2.46	9.99E-04	1.00E+00	9.99E-04	1
279	64	recurrent	T cell	Endothelial	1.44	9.99E-04	1.00E+00	9.99E-04	1
280	64	recurrent	T cell	MES	1.41	9.99E-04	1.00E+00	9.99E-04	1
281	64	recurrent	T cell	Macrophage	1.32	2.00E-03	9.99E-01	2.00E-03	1
282	64	recurrent	T cell	Microglia	1.17	9.99E-02	9.07E-01	9.99E-02	0
283	64	recurrent	T cell	NK cell	1.26	7.99E-03	9.93E-01	7.99E-03	1
284	64	recurrent	T cell	NPC	1.45	9.99E-04	1.00E+00	9.99E-04	1
285	64	recurrent	T cell	Neuron	1.26	3.00E-03	9.98E-01	3.00E-03	1
286	64	recurrent	T cell	OPC	0.00	1.00E+00	7.29E-01	7.29E-01	0
287	64	recurrent	T cell	Oligodendrocyte	2.52	9.99E-04	1.00E+00	9.99E-04	1
288	64	recurrent	T cell	T cell	1.47	9.99E-04	1.00E+00	9.99E-04	1
289	67	primary	AC	AC	1.00	4.58E-01	9.87E-01	4.58E-01	0
290	67	primary	AC	Astrocyte	1.14	4.35E-01	6.06E-01	4.35E-01	0
291	67	primary	AC	Endothelial	1.00	1.00E+00	2.10E-01	2.10E-01	0
292	67	primary	AC	MES	0.00	1.00E+00	8.79E-02	8.79E-02	0
293	67	primary	AC	Macrophage	1.00	9.99E-01	5.45E-01	5.45E-01	0
294	67	primary	AC	Microglia	1.26	3.92E-01	6.10E-01	3.92E-01	0
295	67	primary	AC	NK cell	1.33	1.00E+00	9.99E-04	9.99E-04	-1
296	67	primary	AC	NPC	1.00	9.95E-01	7.84E-01	7.84E-01	0
297	67	primary	AC	Neuron	1.64	4.90E-02	9.54E-01	4.90E-02	0
298	67	primary	AC	OPC	1.74	4.00E-03	9.97E-01	4.00E-03	1
299	67	primary	AC	Oligodendrocyte	1.80	5.99E-03	9.95E-01	5.99E-03	1
300	67	primary	AC	T cell	1.13	6.03E-01	4.34E-01	4.34E-01	0
301	67	primary	Astrocyte	AC	1.00	1.00E+00	8.78E-01	8.78E-01	0
302	67	primary	Astrocyte	Astrocyte	1.49	9.99E-04	1.00E+00	9.99E-04	1
303	67	primary	Astrocyte	Endothelial	1.19	4.70E-02	9.56E-01	4.70E-02	0
304	67	primary	Astrocyte	MES	1.26	9.99E-04	1.00E+00	9.99E-04	1
305	67	primary	Astrocyte	Macrophage	1.10	2.26E-01	7.86E-01	2.26E-01	0
306	67	primary	Astrocyte	Microglia	1.40	9.99E-04	1.00E+00	9.99E-04	1
307	67	primary	Astrocyte	NK cell	1.76	1.00E+00	9.99E-04	9.99E-04	-1
308	67	primary	Astrocyte	NPC	1.08	1.54E-01	8.54E-01	1.54E-01	0
309	67	primary	Astrocyte	Neuron	1.91	9.99E-04	1.00E+00	9.99E-04	1
310	67	primary	Astrocyte	OPC	1.48	9.99E-04	1.00E+00	9.99E-04	1
311	67	primary	Astrocyte	Oligodendrocyte	1.48	1.20E-02	9.89E-01	1.20E-02	0
312	67	primary	Astrocyte	T cell	1.17	4.07E-01	5.94E-01	4.07E-01	0
313	67	primary	Endothelial	AC	1.00	1.00E+00	8.69E-01	8.69E-01	0
314	67	primary	Endothelial	Astrocyte	1.23	3.00E-03	9.98E-01	3.00E-03	1
315	67	primary	Endothelial	Endothelial	2.38	9.99E-04	1.00E+00	9.99E-04	1
316	67	primary	Endothelial	MES	1.00	1.00E+00	5.87E-01	5.87E-01	0
317	67	primary	Endothelial	Macrophage	1.10	2.01E-01	8.06E-01	2.01E-01	0
318	67	primary	Endothelial	Microglia	1.23	6.12E-01	3.95E-01	3.95E-01	0
319	67	primary	Endothelial	NK cell	3.68	9.99E-04	1.00E+00	9.99E-04	1
320	67	primary	Endothelial	NPC	1.00	1.00E+00	5.79E-02	5.79E-02	0

row	patient	surgery	from cell type	to cell tye	observed count	permutations (greater than)	permutations (less than)	p value	interaction significance/direction
321	67	primary	Endothelial	Neuron	1.54	6.99E-03	9.94E-01	6.99E-03	1
322	67	primary	Endothelial	OPC	1.44	9.99E-04	1.00E+00	9.99E-04	1
323	67	primary	Endothelial	Oligodendrocyte	1.35	9.25E-01	7.59E-02	7.59E-02	0
324	67	primary	Endothelial	T cell	1.17	3.76E-01	6.25E-01	3.76E-01	0
325	67	primary	MES	AC	0.00	1.00E+00	8.79E-02	8.79E-02	0
326	67	primary	MES	Astrocyte	1.55	2.00E-03	9.99E-01	2.00E-03	1
327	67	primary	MES	Endothelial	1.50	9.99E-04	1.00E+00	9.99E-04	1
328	67	primary	MES	MES	1.68	3.00E-03	9.98E-01	3.00E-03	1
329	67	primary	MES	Macrophage	1.27	2.60E-02	9.78E-01	2.60E-02	0
330	67	primary	MES	Microglia	1.00	1.00E+00	9.99E-04	9.99E-04	-1
331	67	primary	MES	NK cell	3.20	9.99E-04	1.00E+00	9.99E-04	1
332	67	primary	MES	NPC	0.00	1.00E+00	9.99E-04	9.99E-04	-1
333	67	primary	MES	Neuron	1.00	1.00E+00	9.99E-04	9.99E-04	-1
334	67	primary	MES	OPC	0.00	1.00E+00	9.99E-04	9.99E-04	-1
335	67	primary	MES	Oligodendrocyte	1.29	9.11E-01	9.29E-02	9.29E-02	0
336	67	primary	MES	T cell	1.32	4.50E-02	9.57E-01	4.50E-02	0
337	67	primary	Macrophage	AC	1.00	9.99E-01	9.13E-01	9.13E-01	0
338	67	primary	Macrophage	Astrocyte	1.33	9.99E-04	1.00E+00	9.99E-04	1
339	67	primary	Macrophage	Endothelial	1.39	9.99E-04	1.00E+00	9.99E-04	1
340	67	primary	Macrophage	MES	1.27	2.00E-03	9.99E-01	2.00E-03	1
341	67	primary	Macrophage	Macrophage	1.04	7.80E-01	2.21E-01	2.21E-01	0
342	67	primary	Macrophage	Microglia	1.40	9.99E-04	1.00E+00	9.99E-04	1
343	67	primary	Macrophage	NK cell	2.28	9.99E-04	1.00E+00	9.99E-04	1
344	67	primary	Macrophage	NPC	1.19	6.99E-03	9.95E-01	6.99E-03	1
345	67	primary	Macrophage	Neuron	1.69	9.99E-04	1.00E+00	9.99E-04	1
346	67	primary	Macrophage	OPC	1.48	9.99E-04	1.00E+00	9.99E-04	1
347	67	primary	Macrophage	Oligodendrocyte	1.67	9.99E-04	1.00E+00	9.99E-04	1
348	67	primary	Macrophage	T cell	1.25	2.70E-02	9.74E-01	2.70E-02	0
349	67	primary	Microglia	AC	1.00	1.00E+00	7.64E-01	7.64E-01	0
350	67	primary	Microglia	Astrocyte	1.29	9.99E-04	1.00E+00	9.99E-04	1
351	67	primary	Microglia	Endothelial	1.26	9.99E-04	1.00E+00	9.99E-04	1
352	67	primary	Microglia	MES	1.25	9.99E-04	1.00E+00	9.99E-04	1
353	67	primary	Microglia	Macrophage	1.15	3.00E-03	9.98E-01	3.00E-03	1
354	67	primary	Microglia	Microglia	1.47	9.99E-04	1.00E+00	9.99E-04	1
355	67	primary	Microglia	NK cell	1.53	1.00E+00	9.99E-04	9.99E-04	-1
356	67	primary	Microglia	NPC	1.06	3.52E-01	6.62E-01	3.52E-01	0
357	67	primary	Microglia	Neuron	1.71	9.99E-04	1.00E+00	9.99E-04	1
358	67	primary	Microglia	OPC	1.55	9.99E-04	1.00E+00	9.99E-04	1
359	67	primary	Microglia	Oligodendrocyte	1.66	9.99E-04	1.00E+00	9.99E-04	1
360	67	primary	Microglia	T cell	1.33	9.99E-04	1.00E+00	9.99E-04	1
361	67	primary	NK cell	AC	1.04	5.00E-02	9.54E-01	5.00E-02	0
362	67	primary	NK cell	Astrocyte	1.37	9.99E-04	1.00E+00	9.99E-04	1
363	67	primary	NK cell	Endothelial	1.73	9.99E-04	1.00E+00	9.99E-04	1
364	67	primary	NK cell	MES	1.38	9.99E-04	1.00E+00	9.99E-04	1
365	67	primary	NK cell	Macrophage	1.09	2.61E-01	7.40E-01	2.61E-01	0
366	67	primary	NK cell	Microglia	1.34	9.99E-04	1.00E+00	9.99E-04	1
367	67	primary	NK cell	NK cell	4.32	9.99E-04	1.00E+00	9.99E-04	1
368	67	primary	NK cell	NPC	1.13	9.99E-04	1.00E+00	9.99E-04	1
369	67	primary	NK cell	Neuron	1.61	9.99E-04	1.00E+00	9.99E-04	1
370	67	primary	NK cell	OPC	1.47	9.99E-04	1.00E+00	9.99E-04	1
371	67	primary	NK cell	Oligodendrocyte	1.56	9.99E-04	1.00E+00	9.99E-04	1
372	67	primary	NK cell	T cell	1.24	9.99E-04	1.00E+00	9.99E-04	1
373	67	primary	NPC	AC	1.00	9.95E-01	9.54E-01	9.54E-01	0
374	67	primary	NPC	Astrocyte	1.26	2.00E-02	9.81E-01	2.00E-02	0
375	67	primary	NPC	Endothelial	1.42	9.99E-04	1.00E+00	9.99E-04	1
376	67	primary	NPC	MES	0.00	1.00E+00	9.99E-04	9.99E-04	-1
377	67	primary	NPC	Macrophage	1.19	2.70E-02	9.74E-01	2.70E-02	0
378	67	primary	NPC	Microglia	1.25	4.00E-01	6.03E-01	4.00E-01	0
379	67	primary	NPC	NK cell	1.28	1.00E+00	9.99E-04	9.99E-04	-1
380	67	primary	NPC	NPC	1.12	1.54E-01	8.47E-01	1.54E-01	0
381	67	primary	NPC	Neuron	1.78	9.99E-04	1.00E+00	9.99E-04	1
382	67	primary	NPC	OPC	1.67	9.99E-04	1.00E+00	9.99E-04	1
383	67	primary	NPC	Oligodendrocyte	1.78	9.99E-04	1.00E+00	9.99E-04	1
384	67	primary	NPC	T cell	1.16	5.14E-01	4.90E-01	4.90E-01	0
385	67	primary	Neuron	AC	1.07	1.80E-02	9.83E-01	1.80E-02	0
386	67	primary	Neuron	Astrocyte	1.35	9.99E-04	1.00E+00	9.99E-04	1
387	67	primary	Neuron	Endothelial	1.30	9.99E-04	1.00E+00	9.99E-04	1
388	67	primary	Neuron	MES	1.00	1.00E+00	1.75E-01	1.75E-01	0
389	67	primary	Neuron	Macrophage	1.15	9.99E-04	1.00E+00	9.99E-04	1
390	67	primary	Neuron	Microglia	1.32	9.99E-04	1.00E+00	9.99E-04	1
391	67	primary	Neuron	NK cell	1.29	1.00E+00	9.99E-04	9.99E-04	-1
392	67	primary	Neuron	NPC	1.15	9.99E-04	1.00E+00	9.99E-04	1
393	67	primary	Neuron	Neuron	1.98	9.99E-04	1.00E+00	9.99E-04	1
394	67	primary	Neuron	OPC	1.53	9.99E-04	1.00E+00	9.99E-04	1
395	67	primary	Neuron	Oligodendrocyte	1.70	9.99E-04	1.00E+00	9.99E-04	1
396	67	primary	Neuron	T cell	1.22	2.00E-03	9.99E-01	2.00E-03	1
397	67	primary	OPC	AC	1.02	2.56E-01	7.45E-01	2.56E-01	0
398	67	primary	OPC	Astrocyte	1.34	9.99E-04	1.00E+00	9.99E-04	1
399	67	primary	OPC	Endothelial	1.17	8.79E-02	9.14E-01	8.79E-02	0
400	67	primary	OPC	MES	0.00	1.00E+00	9.99E-04	9.99E-04	-1
401	67	primary	OPC	Macrophage	1.12	4.50E-02	9.56E-01	4.50E-02	0
402	67	primary	OPC	Microglia	1.37	9.99E-04	1.00E+00	9.99E-04	1
403	67	primary	OPC	NK cell	1.29	1.00E+00	9.99E-04	9.99E-04	-1
404	67	primary	OPC	NPC	1.10	1.50E-02	9.86E-01	1.50E-02	0
405	67	primary	OPC	Neuron	1.76	9.99E-04	1.00E+00	9.99E-04	1
406	67	primary	OPC	OPC	1.75	9.99E-04	1.00E+00	9.99E-04	1
407	67	primary	OPC	Oligodendrocyte	1.70	9.99E-04	1.00E+00	9.99E-04	1
408	67	primary	OPC	T cell	1.24	9.99E-04	1.00E+00	9.99E-04	1
409	67	primary	Oligodendrocyte	AC	1.05	6.39E-02	9.40E-01	6.39E-02	0
410	67	primary	Oligodendrocyte	Astrocyte	1.28	9.99E-04	1.00E+00	9.99E-04	1
411	67	primary	Oligodendrocyte	Endothelial	1.25	9.99E-04	1.00E+00	9.99E-04	1
412	67	primary	Oligodendrocyte	MES	1.50	9.99E-04	1.00E+00	9.99E-04	1
413	67	primary	Oligodendrocyte	Macrophage	1.14	9.99E-04	1.00E+00	9.99E-04	1
414	67	primary	Oligodendrocyte	Microglia	1.34	9.99E-04	1.00E+00	9.99E-04	1
415	67	primary	Oligodendrocyte	NK cell	1.37	1.00E+00	9.99E-04	9.99E-04	-1
416	67	primary	Oligodendrocyte	NPC	1.11	2.00E-03	9.99E-01	2.00E-03	1
417	67	primary	Oligodendrocyte	Neuron	1.78	9.99E-04	1.00E+00	9.99E-04	1
418	67	primary	Oligodendrocyte	OPC	1.57	9.99E-04	1.00E+00	9.99E-04	1
419	67	primary	Oligodendrocyte	Oligodendrocyte	1.91	9.99E-04	1.00E+00	9.99E-04	1
420	67	primary	Oligodendrocyte	T cell	1.25	9.99E-04	1.00E+00	9.99E-04	1
421	67	primary	T cell	AC	1.06	1.06E-01	9.00E-01	1.06E-01	0
422	67	primary	T cell	Astrocyte	1.32	9.99E-04	1.00E+00	9.99E-04	1
423	67	primary	T cell	Endothelial	1.34	9.99E-04	1.00E+00	9.99E-04	1
424	67	primary	T cell	MES	1.57	9.99E-04	1.00E+00	9.99E-04	1
425	67	primary	T cell	Macrophage	1.12	6.89E-02	9.32E-01	6.89E-02	0
426	67	primary	T cell	Microglia	1.38	9.99E-04	1.00E+00	9.99E-04	1
427	67	primary	T cell	NK cell	2.11	4.80E-02	9.53E-01	4.80E-02	0
428	67	primary	T cell	NPC	1.09	5.79E-02	9.44E-01	5.79E-02	0
429	67	primary	T cell	Neuron	1.74	9.99E-04	1.00E+00	9.99E-04	1
430	67	primary	T cell	OPC	1.54	9.99E-04	1.00E+00	9.99E-04	1
431	67	primary	T cell	Oligodendrocyte	1.71	9.99E-04	1.00E+00	9.99E-04	1

row	patient	surgery	from cell type	to cell tye	observed count	permutations (greater than)	permutations (less than)	p value	interaction significance/direction
432	67	primary	T cell	T cell	1.34	9.99E-04	1.00E+00	9.99E-04	1
433	67	recurrent	AC	AC					
434	67	recurrent	AC	Astrocyte					
435	67	recurrent	AC	Endothelial					
436	67	recurrent	AC	MES					
437	67	recurrent	AC	Macrophage					
438	67	recurrent	AC	Microglia					
439	67	recurrent	AC	NK cell					
440	67	recurrent	AC	NPC					
441	67	recurrent	AC	Neuron					
442	67	recurrent	AC	OPC					
443	67	recurrent	AC	Oligodendrocyte					
444	67	recurrent	AC	T cell					
445	67	recurrent	Astrocyte	AC					
446	67	recurrent	Astrocyte	Astrocyte	3.83	9.99E-04	1.00E+00	9.99E-04	1
447	67	recurrent	Astrocyte	Endothelial	1.37	9.99E-04	1.00E+00	9.99E-04	1
448	67	recurrent	Astrocyte	MES	1.05	7.29E-02	9.28E-01	7.29E-02	0
449	67	recurrent	Astrocyte	Macrophage	1.25	2.00E-03	9.99E-01	2.00E-03	1
450	67	recurrent	Astrocyte	Microglia	1.30	9.99E-04	1.00E+00	9.99E-04	1
451	67	recurrent	Astrocyte	NK cell	1.27	9.99E-04	1.00E+00	9.99E-04	1
452	67	recurrent	Astrocyte	NPC	1.06	4.00E-03	9.97E-01	4.00E-03	1
453	67	recurrent	Astrocyte	Neuron	1.16	9.99E-04	1.00E+00	9.99E-04	1
454	67	recurrent	Astrocyte	OPC	1.07	4.00E-03	9.98E-01	4.00E-03	1
455	67	recurrent	Astrocyte	Oligodendrocyte	1.20	9.99E-04	1.00E+00	9.99E-04	1
456	67	recurrent	Astrocyte	T cell	1.20	9.99E-04	1.00E+00	9.99E-04	1
457	67	recurrent	Endothelial	AC					
458	67	recurrent	Endothelial	Astrocyte	2.10	1.00E+00	9.99E-04	9.99E-04	-1
459	67	recurrent	Endothelial	Endothelial	3.57	9.99E-04	1.00E+00	9.99E-04	1
460	67	recurrent	Endothelial	MES	1.12	9.99E-04	1.00E+00	9.99E-04	1
461	67	recurrent	Endothelial	Macrophage	1.27	8.99E-03	9.92E-01	8.99E-03	1
462	67	recurrent	Endothelial	Microglia	1.38	9.99E-04	1.00E+00	9.99E-04	1
463	67	recurrent	Endothelial	NK cell	1.34	9.99E-04	1.00E+00	9.99E-04	1
464	67	recurrent	Endothelial	NPC	1.20	9.99E-04	1.00E+00	9.99E-04	1
465	67	recurrent	Endothelial	Neuron	1.12	1.56E-01	8.45E-01	1.56E-01	0
466	67	recurrent	Endothelial	OPC	1.00	9.99E-01	9.83E-01	9.83E-01	0
467	67	recurrent	Endothelial	Oligodendrocyte	1.14	5.00E-03	9.97E-01	5.00E-03	1
468	67	recurrent	Endothelial	T cell	1.20	5.49E-02	9.46E-01	5.49E-02	0
469	67	recurrent	MES	AC					
470	67	recurrent	MES	Astrocyte	3.42	3.00E-02	9.71E-01	3.00E-02	0
471	67	recurrent	MES	Endothelial	1.93	9.99E-04	1.00E+00	9.99E-04	1
472	67	recurrent	MES	MES	1.10	1.29E-01	8.72E-01	1.29E-01	0
473	67	recurrent	MES	Macrophage	1.27	1.48E-01	8.56E-01	1.48E-01	0
474	67	recurrent	MES	Microglia	1.31	1.74E-01	8.28E-01	1.74E-01	0
475	67	recurrent	MES	NK cell	1.38	6.99E-03	9.94E-01	6.99E-03	1
476	67	recurrent	MES	NPC	1.00	9.98E-01	8.90E-01	8.90E-01	0
477	67	recurrent	MES	Neuron	1.19	7.79E-02	9.26E-01	7.79E-02	0
478	67	recurrent	MES	OPC	0.00	1.00E+00	3.37E-01	3.37E-01	0
479	67	recurrent	MES	Oligodendrocyte	1.25	8.99E-03	9.96E-01	8.99E-03	1
480	67	recurrent	MES	T cell	1.33	1.80E-02	9.83E-01	1.80E-02	0
481	67	recurrent	Macrophage	AC					
482	67	recurrent	Macrophage	Astrocyte	2.95	1.00E+00	9.99E-04	9.99E-04	-1
483	67	recurrent	Macrophage	Endothelial	1.65	9.99E-04	1.00E+00	9.99E-04	1
484	67	recurrent	Macrophage	MES	1.10	1.60E-02	9.85E-01	1.60E-02	0
485	67	recurrent	Macrophage	Macrophage	1.60	9.99E-04	1.00E+00	9.99E-04	1
486	67	recurrent	Macrophage	Microglia	1.56	9.99E-04	1.00E+00	9.99E-04	1
487	67	recurrent	Macrophage	NK cell	1.40	9.99E-04	1.00E+00	9.99E-04	1
488	67	recurrent	Macrophage	NPC	1.00	1.00E+00	6.29E-01	6.29E-01	0
489	67	recurrent	Macrophage	Neuron	1.15	3.80E-02	9.64E-01	3.80E-02	0
490	67	recurrent	Macrophage	OPC	1.00	9.98E-01	9.87E-01	9.87E-01	0
491	67	recurrent	Macrophage	Oligodendrocyte	1.15	2.00E-03	9.99E-01	2.00E-03	1
492	67	recurrent	Macrophage	T cell	1.19	8.99E-02	9.12E-01	8.99E-02	0
493	67	recurrent	Microglia	AC					
494	67	recurrent	Microglia	Astrocyte	2.87	1.00E+00	9.99E-04	9.99E-04	-1
495	67	recurrent	Microglia	Endothelial	1.74	9.99E-04	1.00E+00	9.99E-04	1
496	67	recurrent	Microglia	MES	1.06	1.90E-01	8.11E-01	1.90E-01	0
497	67	recurrent	Microglia	Macrophage	1.41	9.99E-04	1.00E+00	9.99E-04	1
498	67	recurrent	Microglia	Microglia	1.64	9.99E-04	1.00E+00	9.99E-04	1
499	67	recurrent	Microglia	NK cell	1.41	9.99E-04	1.00E+00	9.99E-04	1
500	67	recurrent	Microglia	NPC	1.18	9.99E-04	1.00E+00	9.99E-04	1
501	67	recurrent	Microglia	Neuron	1.13	8.39E-02	9.17E-01	8.39E-02	0
502	67	recurrent	Microglia	OPC	1.33	9.99E-04	1.00E+00	9.99E-04	1
503	67	recurrent	Microglia	Oligodendrocyte	1.30	9.99E-04	1.00E+00	9.99E-04	1
504	67	recurrent	Microglia	T cell	1.29	9.99E-04	1.00E+00	9.99E-04	1
505	67	recurrent	NK cell	AC					
506	67	recurrent	NK cell	Astrocyte	2.99	1.00E+00	9.99E-04	9.99E-04	-1
507	67	recurrent	NK cell	Endothelial	1.75	9.99E-04	1.00E+00	9.99E-04	1
508	67	recurrent	NK cell	MES	1.06	2.11E-01	7.95E-01	2.11E-01	0
509	67	recurrent	NK cell	Macrophage	1.41	9.99E-04	1.00E+00	9.99E-04	1
510	67	recurrent	NK cell	Microglia	1.48	9.99E-04	1.00E+00	9.99E-04	1
511	67	recurrent	NK cell	NK cell	1.61	9.99E-04	1.00E+00	9.99E-04	1
512	67	recurrent	NK cell	NPC	1.00	1.00E+00	6.12E-01	6.12E-01	0
513	67	recurrent	NK cell	Neuron	1.07	7.96E-01	2.05E-01	2.05E-01	0
514	67	recurrent	NK cell	OPC	1.00	9.97E-01	9.80E-01	9.80E-01	0
515	67	recurrent	NK cell	Oligodendrocyte	1.26	9.99E-04	1.00E+00	9.99E-04	1
516	67	recurrent	NK cell	T cell	1.25	2.00E-03	9.99E-01	2.00E-03	1
517	67	recurrent	NPC	AC					
518	67	recurrent	NPC	Astrocyte	3.68	9.99E-04	1.00E+00	9.99E-04	1
519	67	recurrent	NPC	Endothelial	1.00	1.00E+00	3.00E-03	3.00E-03	-1
520	67	recurrent	NPC	MES	1.00	9.98E-01	8.27E-01	8.27E-01	0
521	67	recurrent	NPC	Macrophage	1.28	2.05E-01	7.99E-01	2.05E-01	0
522	67	recurrent	NPC	Microglia	1.39	7.49E-02	9.31E-01	7.49E-02	0
523	67	recurrent	NPC	NK cell	1.14	7.22E-01	3.07E-01	3.07E-01	0
524	67	recurrent	NPC	NPC	1.00	7.44E-01	9.71E-01	7.44E-01	0
525	67	recurrent	NPC	Neuron	1.30	2.60E-02	9.80E-01	2.60E-02	0
526	67	recurrent	NPC	OPC	1.00	4.24E-01	9.99E-01	4.24E-01	0
527	67	recurrent	NPC	Oligodendrocyte	1.25	5.00E-02	9.67E-01	5.00E-02	0
528	67	recurrent	NPC	T cell	1.25	1.75E-01	8.48E-01	1.75E-01	0
529	67	recurrent	Neuron	AC					
530	67	recurrent	Neuron	Astrocyte	3.58	9.99E-04	1.00E+00	9.99E-04	1
531	67	recurrent	Neuron	Endothelial	1.56	9.99E-04	1.00E+00	9.99E-04	1
532	67	recurrent	Neuron	MES	1.12	3.10E-02	9.72E-01	3.10E-02	0
533	67	recurrent	Neuron	Macrophage	1.32	7.99E-03	9.93E-01	7.99E-03	1
534	67	recurrent	Neuron	Microglia	1.25	4.82E-01	5.19E-01	4.82E-01	0
535	67	recurrent	Neuron	NK cell	1.18	6.12E-01	3.89E-01	3.89E-01	0
536	67	recurrent	Neuron	NPC	1.00	1.00E+00	7.72E-01	7.72E-01	0
537	67	recurrent	Neuron	Neuron	1.18	6.89E-02	9.36E-01	6.89E-02	0
538	67	recurrent	Neuron	OPC	1.00	9.36E-01	9.89E-01	9.36E-01	0
539	67	recurrent	Neuron	Oligodendrocyte	1.15	1.30E-02	9.90E-01	1.30E-02	0
540	67	recurrent	Neuron	T cell	1.18	3.12E-01	6.92E-01	3.12E-01	0
541	67	recurrent	OPC	AC					
542	67	recurrent	OPC	Astrocyte	2.67	9.29E-01	7.59E-02	7.59E-02	0

row	patient	surgery	from cell type	to cell tye	observed count	permutations (greater than)	permutations (less than)	p value	interaction significance/direction
543	67	recurrent	OPC	Endothelial	3.50	9.99E-04	1.00E+00	9.99E-04	1
544	67	recurrent	OPC	MES	0.00	1.00E+00	3.37E-01	3.37E-01	0
545	67	recurrent	OPC	Macrophage	1.00	9.98E-01	4.19E-01	4.19E-01	0
546	67	recurrent	OPC	Microglia	1.00	9.98E-01	2.88E-01	2.88E-01	0
547	67	recurrent	OPC	NK cell	1.50	1.51E-01	9.24E-01	1.51E-01	0
548	67	recurrent	OPC	NPC	1.00	4.24E-01	9.92E-01	4.24E-01	0
549	67	recurrent	OPC	Neuron	1.33	1.39E-01	9.12E-01	1.39E-01	0
550	67	recurrent	OPC	OPC	0.00	1.00E+00	9.59E-01	9.59E-01	0
551	67	recurrent	OPC	Oligodendrocyte	0.00	1.00E+00	1.74E-01	1.74E-01	0
552	67	recurrent	OPC	T cell	1.20	3.66E-01	6.97E-01	3.66E-01	0
553	67	recurrent	Oligodendrocyte	AC					
554	67	recurrent	Oligodendrocyte	Astrocyte	3.25	2.64E-01	7.37E-01	2.64E-01	0
555	67	recurrent	Oligodendrocyte	Endothelial	1.90	9.99E-04	1.00E+00	9.99E-04	1
556	67	recurrent	Oligodendrocyte	MES	1.00	1.00E+00	5.24E-01	5.24E-01	0
557	67	recurrent	Oligodendrocyte	Macrophage	1.23	2.71E-01	7.30E-01	2.71E-01	0
558	67	recurrent	Oligodendrocyte	Microglia	1.36	3.70E-02	9.64E-01	3.70E-02	0
559	67	recurrent	Oligodendrocyte	NK cell	1.32	1.30E-02	9.88E-01	1.30E-02	0
560	67	recurrent	Oligodendrocyte	NPC	1.00	1.00E+00	8.61E-01	8.61E-01	0
561	67	recurrent	Oligodendrocyte	Neuron	1.36	9.99E-04	1.00E+00	9.99E-04	1
562	67	recurrent	Oligodendrocyte	OPC	0.00	1.00E+00	1.74E-01	1.74E-01	0
563	67	recurrent	Oligodendrocyte	Oligodendrocyte	1.50	9.99E-04	1.00E+00	9.99E-04	1
564	67	recurrent	Oligodendrocyte	T cell	1.21	1.60E-01	8.44E-01	1.60E-01	0
565	67	recurrent	T cell	AC					
566	67	recurrent	T cell	Astrocyte	3.30	2.80E-02	9.73E-01	2.80E-02	0
567	67	recurrent	T cell	Endothelial	1.48	9.99E-04	1.00E+00	9.99E-04	1
568	67	recurrent	T cell	MES	1.15	3.00E-03	9.99E-01	3.00E-03	1
569	67	recurrent	T cell	Macrophage	1.30	9.99E-04	1.00E+00	9.99E-04	1
570	67	recurrent	T cell	Microglia	1.47	9.99E-04	1.00E+00	9.99E-04	1
571	67	recurrent	T cell	NK cell	1.41	9.99E-04	1.00E+00	9.99E-04	1
572	67	recurrent	T cell	NPC	1.09	3.20E-02	9.79E-01	3.20E-02	0
573	67	recurrent	T cell	Neuron	1.24	9.99E-04	1.00E+00	9.99E-04	1
574	67	recurrent	T cell	OPC	1.00	9.93E-01	9.88E-01	9.88E-01	0
575	67	recurrent	T cell	Oligodendrocyte	1.31	9.99E-04	1.00E+00	9.99E-04	1
576	67	recurrent	T cell	T cell	1.26	1.30E-02	9.88E-01	1.30E-02	0
577	71	primary	AC	AC	1.00	9.99E-04	1.00E+00	9.99E-04	1
578	71	primary	AC	Astrocyte	0.00	1.00E+00	9.48E-01	9.48E-01	0
579	71	primary	AC	Endothelial	0.00	1.00E+00	1.10E-02	1.10E-02	0
580	71	primary	AC	MES	3.00	3.00E-03	1.00E+00	3.00E-03	1
581	71	primary	AC	Macrophage	4.33	3.46E-01	7.77E-01	3.46E-01	0
582	71	primary	AC	Microglia	0.00	1.00E+00	2.55E-01	2.55E-01	0
583	71	primary	AC	NK cell	0.00	1.00E+00	9.58E-01	9.58E-01	0
584	71	primary	AC	NPC					
585	71	primary	AC	Neuron					
586	71	primary	AC	OPC					
587	71	primary	AC	Oligodendrocyte	0.00	1.00E+00	9.85E-01	9.85E-01	0
588	71	primary	AC	T cell	0.00	1.00E+00	7.56E-01	7.56E-01	0
589	71	primary	Astrocyte	AC	0.00	1.00E+00	9.48E-01	9.48E-01	0
590	71	primary	Astrocyte	Astrocyte	1.30	5.00E-03	9.96E-01	5.00E-03	1
591	71	primary	Astrocyte	Endothelial	1.67	5.71E-01	4.63E-01	4.63E-01	0
592	71	primary	Astrocyte	MES	1.35	4.10E-02	9.60E-01	4.10E-02	0
593	71	primary	Astrocyte	Macrophage	3.13	1.00E+00	9.99E-04	9.99E-04	-1
594	71	primary	Astrocyte	Microglia	1.38	1.07E-01	9.03E-01	1.07E-01	0
595	71	primary	Astrocyte	NK cell	1.00	4.64E-01	9.96E-01	4.64E-01	0
596	71	primary	Astrocyte	NPC					
597	71	primary	Astrocyte	Neuron					
598	71	primary	Astrocyte	OPC					
599	71	primary	Astrocyte	Oligodendrocyte	0.00	1.00E+00	7.43E-01	7.43E-01	0
600	71	primary	Astrocyte	T cell	1.00	9.82E-01	8.36E-01	8.36E-01	0
601	71	primary	Endothelial	AC	0.00	1.00E+00	1.10E-02	1.10E-02	0
602	71	primary	Endothelial	Astrocyte	1.33	9.99E-04	1.00E+00	9.99E-04	1
603	71	primary	Endothelial	Endothelial	3.60	9.99E-04	1.00E+00	9.99E-04	1
604	71	primary	Endothelial	MES	1.19	9.99E-04	1.00E+00	9.99E-04	1
605	71	primary	Endothelial	Macrophage	2.49	1.00E+00	9.99E-04	9.99E-04	-1
606	71	primary	Endothelial	Microglia	1.41	9.99E-04	1.00E+00	9.99E-04	1
607	71	primary	Endothelial	NK cell	1.00	1.00E+00	7.58E-01	7.58E-01	0
608	71	primary	Endothelial	NPC					
609	71	primary	Endothelial	Neuron					
610	71	primary	Endothelial	OPC					
611	71	primary	Endothelial	Oligodendrocyte	1.00	1.00E+00	9.61E-01	9.61E-01	0
612	71	primary	Endothelial	T cell	1.07	5.00E-02	9.51E-01	5.00E-02	0
613	71	primary	MES	AC	1.00	5.14E-01	1.00E+00	5.14E-01	0
614	71	primary	MES	Astrocyte	1.35	9.99E-04	1.00E+00	9.99E-04	1
615	71	primary	MES	Endothelial	1.44	1.00E+00	9.99E-04	9.99E-04	-1
616	71	primary	MES	MES	1.71	9.99E-04	1.00E+00	9.99E-04	1
617	71	primary	MES	Macrophage	3.96	5.00E-02	9.51E-01	5.00E-02	0
618	71	primary	MES	Microglia	1.33	9.99E-04	1.00E+00	9.99E-04	1
619	71	primary	MES	NK cell	1.00	1.00E+00	9.50E-01	9.50E-01	0
620	71	primary	MES	NPC					
621	71	primary	MES	Neuron					
622	71	primary	MES	OPC					
623	71	primary	MES	Oligodendrocyte	1.00	9.80E-01	9.82E-01	9.80E-01	0
624	71	primary	MES	T cell	1.17	9.99E-04	1.00E+00	9.99E-04	1
625	71	primary	Macrophage	AC	1.18	3.00E-03	9.99E-01	3.00E-03	1
626	71	primary	Macrophage	Astrocyte	1.21	9.99E-04	1.00E+00	9.99E-04	1
627	71	primary	Macrophage	Endothelial	1.80	9.99E-04	1.00E+00	9.99E-04	1
628	71	primary	Macrophage	MES	1.31	9.99E-04	1.00E+00	9.99E-04	1
629	71	primary	Macrophage	Macrophage	4.70	9.99E-04	1.00E+00	9.99E-04	1
630	71	primary	Macrophage	Microglia	1.31	9.99E-04	1.00E+00	9.99E-04	1
631	71	primary	Macrophage	NK cell	1.05	9.99E-04	1.00E+00	9.99E-04	1
632	71	primary	Macrophage	NPC					
633	71	primary	Macrophage	Neuron					
634	71	primary	Macrophage	OPC					
635	71	primary	Macrophage	Oligodendrocyte	1.03	3.50E-02	9.66E-01	3.50E-02	0
636	71	primary	Macrophage	T cell	1.10	9.99E-04	1.00E+00	9.99E-04	1
637	71	primary	Microglia	AC	0.00	1.00E+00	2.55E-01	2.55E-01	0
638	71	primary	Microglia	Astrocyte	1.22	9.99E-04	1.00E+00	9.99E-04	1
639	71	primary	Microglia	Endothelial	2.57	9.99E-04	1.00E+00	9.99E-04	1
640	71	primary	Microglia	MES	1.37	9.99E-04	1.00E+00	9.99E-04	1
641	71	primary	Microglia	Macrophage	3.35	1.00E+00	9.99E-04	9.99E-04	-1
642	71	primary	Microglia	Microglia	1.67	9.99E-04	1.00E+00	9.99E-04	1
643	71	primary	Microglia	NK cell	1.08	2.40E-02	9.82E-01	2.40E-02	0
644	71	primary	Microglia	NPC					
645	71	primary	Microglia	Neuron					
646	71	primary	Microglia	OPC					
647	71	primary	Microglia	Oligodendrocyte	1.00	9.99E-01	9.78E-01	9.78E-01	0
648	71	primary	Microglia	T cell	1.10	5.99E-03	9.95E-01	5.99E-03	1
649	71	primary	NK cell	AC	0.00	1.00E+00	9.58E-01	9.58E-01	0
650	71	primary	NK cell	Astrocyte	1.00	4.64E-01	9.96E-01	4.64E-01	0
651	71	primary	NK cell	Endothelial	2.27	9.99E-04	1.00E+00	9.99E-04	1
652	71	primary	NK cell	MES	1.50	1.30E-02	9.94E-01	1.30E-02	0
653	71	primary	NK cell	Macrophage	3.44	9.68E-01	3.30E-02	3.30E-02	0

row	patient	surgery	from cell type	to cell tye	observed count	permutations (greater than)	permutations (less than)	p value	interaction significance/direction
654	71	primary	NK cell	Microglia	1.44	5.29E-02	9.50E-01	5.29E-02	0
655	71	primary	NK cell	NK cell	1.00	2.40E-01	9.96E-01	2.40E-01	0
656	71	primary	NK cell	NPC					
657	71	primary	NK cell	Neuron					
658	71	primary	NK cell	OPC					
659	71	primary	NK cell	Oligodendrocyte	0.00	1.00E+00	7.53E-01	7.53E-01	0
660	71	primary	NK cell	T cell	1.00	9.70E-01	8.62E-01	8.62E-01	0
661	71	primary	NPC	AC					
662	71	primary	NPC	Astrocyte					
663	71	primary	NPC	Endothelial					
664	71	primary	NPC	MES					
665	71	primary	NPC	Macrophage					
666	71	primary	NPC	Microglia					
667	71	primary	NPC	NK cell					
668	71	primary	NPC	NPC					
669	71	primary	NPC	Neuron					
670	71	primary	NPC	OPC					
671	71	primary	NPC	Oligodendrocyte					
672	71	primary	NPC	T cell					
673	71	primary	Neuron	AC					
674	71	primary	Neuron	Astrocyte					
675	71	primary	Neuron	Endothelial					
676	71	primary	Neuron	MES					
677	71	primary	Neuron	Macrophage					
678	71	primary	Neuron	Microglia					
679	71	primary	Neuron	NK cell					
680	71	primary	Neuron	NPC					
681	71	primary	Neuron	Neuron					
682	71	primary	Neuron	OPC					
683	71	primary	Neuron	Oligodendrocyte					
684	71	primary	Neuron	T cell					
685	71	primary	OPC	AC					
686	71	primary	OPC	Astrocyte					
687	71	primary	OPC	Endothelial					
688	71	primary	OPC	MES					
689	71	primary	OPC	Macrophage					
690	71	primary	OPC	Microglia					
691	71	primary	OPC	NK cell					
692	71	primary	OPC	NPC					
693	71	primary	OPC	Neuron					
694	71	primary	OPC	OPC					
695	71	primary	OPC	Oligodendrocyte					
696	71	primary	OPC	T cell					
697	71	primary	Oligodendrocyte	AC	0.00	1.00E+00	9.85E-01	9.85E-01	0
698	71	primary	Oligodendrocyte	Astrocyte	0.00	1.00E+00	7.43E-01	7.43E-01	0
699	71	primary	Oligodendrocyte	Endothelial	1.20	9.89E-01	1.30E-02	1.30E-02	0
700	71	primary	Oligodendrocyte	MES	1.50	6.99E-02	9.75E-01	6.99E-02	0
701	71	primary	Oligodendrocyte	Macrophage	4.73	5.00E-03	9.96E-01	5.00E-03	1
702	71	primary	Oligodendrocyte	Microglia	1.33	2.71E-01	7.94E-01	2.71E-01	0
703	71	primary	Oligodendrocyte	NK cell	0.00	1.00E+00	7.53E-01	7.53E-01	0
704	71	primary	Oligodendrocyte	NPC					
705	71	primary	Oligodendrocyte	Neuron					
706	71	primary	Oligodendrocyte	OPC					
707	71	primary	Oligodendrocyte	Oligodendrocyte	1.00	5.59E-02	1.00E+00	5.59E-02	0
708	71	primary	Oligodendrocyte	T cell	1.00	7.95E-01	9.46E-01	7.95E-01	0
709	71	primary	T cell	AC	0.00	1.00E+00	7.56E-01	7.56E-01	0
710	71	primary	T cell	Astrocyte	1.00	9.82E-01	9.75E-01	9.75E-01	0
711	71	primary	T cell	Endothelial	2.44	9.99E-04	1.00E+00	9.99E-04	1
712	71	primary	T cell	MES	1.63	9.99E-04	1.00E+00	9.99E-04	1
713	71	primary	T cell	Macrophage	3.53	1.00E+00	9.99E-04	9.99E-04	-1
714	71	primary	T cell	Microglia	1.59	9.99E-04	1.00E+00	9.99E-04	1
715	71	primary	T cell	NK cell	1.33	1.20E-02	9.93E-01	1.20E-02	0
716	71	primary	T cell	NPC					
717	71	primary	T cell	Neuron					
718	71	primary	T cell	OPC					
719	71	primary	T cell	Oligodendrocyte	1.00	7.95E-01	9.96E-01	7.95E-01	0
720	71	primary	T cell	T cell	1.12	1.23E-01	8.84E-01	1.23E-01	0
721	71	recurrent	AC	AC	3.24	9.99E-04	1.00E+00	9.99E-04	1
722	71	recurrent	AC	Astrocyte	1.75	9.99E-04	1.00E+00	9.99E-04	1
723	71	recurrent	AC	Endothelial	1.00	1.00E+00	9.99E-04	9.99E-04	-1
724	71	recurrent	AC	MES	1.61	9.99E-04	1.00E+00	9.99E-04	1
725	71	recurrent	AC	Macrophage	1.10	3.65E-01	6.38E-01	3.65E-01	0
726	71	recurrent	AC	Microglia	1.15	8.25E-01	1.77E-01	1.77E-01	0
727	71	recurrent	AC	NK cell	1.09	9.96E-01	5.00E-03	5.00E-03	-1
728	71	recurrent	AC	NPC	1.33	9.99E-04	1.00E+00	9.99E-04	1
729	71	recurrent	AC	Neuron	1.38	2.00E-03	9.99E-01	2.00E-03	1
730	71	recurrent	AC	OPC	1.21	5.87E-01	4.15E-01	4.15E-01	0
731	71	recurrent	AC	Oligodendrocyte	1.00	1.00E+00	9.99E-04	9.99E-04	-1
732	71	recurrent	AC	T cell	1.26	1.00E+00	9.99E-04	9.99E-04	-1
733	71	recurrent	Astrocyte	AC	2.58	9.99E-04	1.00E+00	9.99E-04	1
734	71	recurrent	Astrocyte	Astrocyte	2.05	9.99E-04	1.00E+00	9.99E-04	1
735	71	recurrent	Astrocyte	Endothelial	1.22	4.00E-03	9.98E-01	4.00E-03	1
736	71	recurrent	Astrocyte	MES	2.29	9.99E-04	1.00E+00	9.99E-04	1
737	71	recurrent	Astrocyte	Macrophage	1.12	1.74E-01	8.32E-01	1.74E-01	0
738	71	recurrent	Astrocyte	Microglia	1.22	1.10E-01	8.91E-01	1.10E-01	0
739	71	recurrent	Astrocyte	NK cell	1.12	8.33E-01	1.71E-01	1.71E-01	0
740	71	recurrent	Astrocyte	NPC	1.28	9.99E-04	1.00E+00	9.99E-04	1
741	71	recurrent	Astrocyte	Neuron	1.34	1.07E-01	8.94E-01	1.07E-01	0
742	71	recurrent	Astrocyte	OPC	1.59	9.99E-04	1.00E+00	9.99E-04	1
743	71	recurrent	Astrocyte	Oligodendrocyte	1.48	9.99E-04	1.00E+00	9.99E-04	1
744	71	recurrent	Astrocyte	T cell	1.60	1.42E-01	8.59E-01	1.42E-01	0
745	71	recurrent	Endothelial	AC	1.50	5.00E-03	9.98E-01	5.00E-03	1
746	71	recurrent	Endothelial	Astrocyte	1.10	9.55E-01	5.00E-02	5.00E-02	0
747	71	recurrent	Endothelial	Endothelial	3.09	9.99E-04	1.00E+00	9.99E-04	1
748	71	recurrent	Endothelial	MES	1.50	1.53E-01	8.58E-01	1.53E-01	0
749	71	recurrent	Endothelial	Macrophage	1.23	8.99E-03	9.92E-01	8.99E-03	1
750	71	recurrent	Endothelial	Microglia	1.65	9.99E-04	1.00E+00	9.99E-04	1
751	71	recurrent	Endothelial	NK cell	1.45	9.99E-04	1.00E+00	9.99E-04	1
752	71	recurrent	Endothelial	NPC	1.00	1.00E+00	2.10E-02	2.10E-02	0
753	71	recurrent	Endothelial	Neuron	1.50	9.99E-04	1.00E+00	9.99E-04	1
754	71	recurrent	Endothelial	OPC	1.38	3.00E-03	9.98E-01	3.00E-03	1
755	71	recurrent	Endothelial	Oligodendrocyte	1.21	8.29E-02	9.18E-01	8.29E-02	0
756	71	recurrent	Endothelial	T cell	1.56	4.25E-01	5.76E-01	4.25E-01	0
757	71	recurrent	MES	AC	1.86	9.99E-04	1.00E+00	9.99E-04	1
758	71	recurrent	MES	Astrocyte	1.55	9.99E-04	1.00E+00	9.99E-04	1
759	71	recurrent	MES	Endothelial	1.34	9.99E-04	1.00E+00	9.99E-04	1
760	71	recurrent	MES	MES	3.00	9.99E-04	1.00E+00	9.99E-04	1
761	71	recurrent	MES	Macrophage	1.22	9.99E-04	1.00E+00	9.99E-04	1
762	71	recurrent	MES	Microglia	1.40	9.99E-04	1.00E+00	9.99E-04	1
763	71	recurrent	MES	NK cell	1.27	9.99E-04	1.00E+00	9.99E-04	1
764	71	recurrent	MES	NPC	1.39	9.99E-04	1.00E+00	9.99E-04	1

row	patient	surgery	from cell type	to cell tye	observed count	permutations (greater than)	permutations (less than)	p value	interaction significance/direction
765	71	recurrent	MES	Neuron	1.56	9.99E-04	1.00E+00	9.99E-04	1
766	71	recurrent	MES	OPC	1.44	9.99E-04	1.00E+00	9.99E-04	1
767	71	recurrent	MES	Oligodendrocyte	1.64	9.99E-04	1.00E+00	9.99E-04	1
768	71	recurrent	MES	T cell	1.77	9.99E-04	1.00E+00	9.99E-04	1
769	71	recurrent	Macrophage	AC	2.61	9.99E-04	1.00E+00	9.99E-04	1
770	71	recurrent	Macrophage	Astrocyte	1.74	9.99E-04	1.00E+00	9.99E-04	1
771	71	recurrent	Macrophage	Endothelial	1.94	9.99E-04	1.00E+00	9.99E-04	1
772	71	recurrent	Macrophage	MES	1.94	9.99E-04	1.00E+00	9.99E-04	1
773	71	recurrent	Macrophage	Macrophage	1.08	4.41E-01	5.60E-01	4.41E-01	0
774	71	recurrent	Macrophage	Microglia	1.61	9.99E-04	1.00E+00	9.99E-04	1
775	71	recurrent	Macrophage	NK cell	1.44	9.99E-04	1.00E+00	9.99E-04	1
776	71	recurrent	Macrophage	NPC	1.56	9.99E-04	1.00E+00	9.99E-04	1
777	71	recurrent	Macrophage	Neuron	1.42	1.70E-02	9.84E-01	1.70E-02	0
778	71	recurrent	Macrophage	OPC	1.58	9.99E-04	1.00E+00	9.99E-04	1
779	71	recurrent	Macrophage	Oligodendrocyte	1.36	3.00E-03	9.98E-01	3.00E-03	1
780	71	recurrent	Macrophage	T cell	1.98	9.99E-04	1.00E+00	9.99E-04	1
781	71	recurrent	Microglia	AC	2.50	9.99E-04	1.00E+00	9.99E-04	1
782	71	recurrent	Microglia	Astrocyte	1.42	9.99E-04	1.00E+00	9.99E-04	1
783	71	recurrent	Microglia	Endothelial	1.70	9.99E-04	1.00E+00	9.99E-04	1
784	71	recurrent	Microglia	MES	1.85	9.99E-04	1.00E+00	9.99E-04	1
785	71	recurrent	Microglia	Macrophage	1.24	2.00E-03	9.99E-01	2.00E-03	1
786	71	recurrent	Microglia	Microglia	1.95	9.99E-04	1.00E+00	9.99E-04	1
787	71	recurrent	Microglia	NK cell	1.52	9.99E-04	1.00E+00	9.99E-04	1
788	71	recurrent	Microglia	NPC	1.29	9.99E-04	1.00E+00	9.99E-04	1
789	71	recurrent	Microglia	Neuron	1.41	3.00E-03	9.98E-01	3.00E-03	1
790	71	recurrent	Microglia	OPC	1.42	9.99E-04	1.00E+00	9.99E-04	1
791	71	recurrent	Microglia	Oligodendrocyte	1.25	6.99E-03	9.94E-01	6.99E-03	1
792	71	recurrent	Microglia	T cell	2.22	9.99E-04	1.00E+00	9.99E-04	1
793	71	recurrent	NK cell	AC	2.00	9.99E-04	1.00E+00	9.99E-04	1
794	71	recurrent	NK cell	Astrocyte	1.33	9.99E-04	1.00E+00	9.99E-04	1
795	71	recurrent	NK cell	Endothelial	1.72	9.99E-04	1.00E+00	9.99E-04	1
796	71	recurrent	NK cell	MES	1.31	9.99E-01	2.00E-03	2.00E-03	-1
797	71	recurrent	NK cell	Macrophage	1.13	1.57E-01	8.47E-01	1.57E-01	0
798	71	recurrent	NK cell	Microglia	1.73	9.99E-04	1.00E+00	9.99E-04	1
799	71	recurrent	NK cell	NK cell	1.87	9.99E-04	1.00E+00	9.99E-04	1
800	71	recurrent	NK cell	NPC	1.00	1.00E+00	5.99E-03	5.99E-03	-1
801	71	recurrent	NK cell	Neuron	1.43	2.00E-03	9.99E-01	2.00E-03	1
802	71	recurrent	NK cell	OPC	1.38	9.99E-04	1.00E+00	9.99E-04	1
803	71	recurrent	NK cell	Oligodendrocyte	1.30	9.99E-04	1.00E+00	9.99E-04	1
804	71	recurrent	NK cell	T cell	2.13	9.99E-04	1.00E+00	9.99E-04	1
805	71	recurrent	NPC	AC	2.34	9.99E-04	1.00E+00	9.99E-04	1
806	71	recurrent	NPC	Astrocyte	1.77	9.99E-04	1.00E+00	9.99E-04	1
807	71	recurrent	NPC	Endothelial	1.50	9.99E-04	1.00E+00	9.99E-04	1
808	71	recurrent	NPC	MES	2.30	9.99E-04	1.00E+00	9.99E-04	1
809	71	recurrent	NPC	Macrophage	1.18	5.49E-02	9.48E-01	5.49E-02	0
810	71	recurrent	NPC	Microglia	1.15	6.85E-01	3.20E-01	3.20E-01	0
811	71	recurrent	NPC	NK cell	1.14	5.71E-01	4.46E-01	4.46E-01	0
812	71	recurrent	NPC	NPC	1.55	9.99E-04	1.00E+00	9.99E-04	1
813	71	recurrent	NPC	Neuron	1.76	9.99E-04	1.00E+00	9.99E-04	1
814	71	recurrent	NPC	OPC	1.40	2.00E-03	9.99E-01	2.00E-03	1
815	71	recurrent	NPC	Oligodendrocyte	1.00	1.00E+00	9.99E-04	9.99E-04	-1
816	71	recurrent	NPC	T cell	1.08	1.00E+00	9.99E-04	9.99E-04	-1
817	71	recurrent	Neuron	AC	1.84	9.99E-04	1.00E+00	9.99E-04	1
818	71	recurrent	Neuron	Astrocyte	1.45	9.99E-04	1.00E+00	9.99E-04	1
819	71	recurrent	Neuron	Endothelial	1.41	9.99E-04	1.00E+00	9.99E-04	1
820	71	recurrent	Neuron	MES	1.84	9.99E-04	1.00E+00	9.99E-04	1
821	71	recurrent	Neuron	Macrophage	1.12	1.56E-01	8.46E-01	1.56E-01	0
822	71	recurrent	Neuron	Microglia	1.18	4.54E-01	5.47E-01	4.54E-01	0
823	71	recurrent	Neuron	NK cell	1.20	8.59E-02	9.19E-01	8.59E-02	0
824	71	recurrent	Neuron	NPC	1.48	9.99E-04	1.00E+00	9.99E-04	1
825	71	recurrent	Neuron	Neuron	2.15	9.99E-04	1.00E+00	9.99E-04	1
826	71	recurrent	Neuron	OPC	1.60	9.99E-04	1.00E+00	9.99E-04	1
827	71	recurrent	Neuron	Oligodendrocyte	1.49	9.99E-04	1.00E+00	9.99E-04	1
828	71	recurrent	Neuron	T cell	1.78	9.99E-04	1.00E+00	9.99E-04	1
829	71	recurrent	OPC	AC	1.81	9.99E-04	1.00E+00	9.99E-04	1
830	71	recurrent	OPC	Astrocyte	1.33	9.99E-04	1.00E+00	9.99E-04	1
831	71	recurrent	OPC	Endothelial	1.29	9.99E-04	1.00E+00	9.99E-04	1
832	71	recurrent	OPC	MES	1.57	2.00E-03	9.99E-01	2.00E-03	1
833	71	recurrent	OPC	Macrophage	1.06	8.53E-01	1.54E-01	1.54E-01	0
834	71	recurrent	OPC	Microglia	1.22	1.11E-01	8.90E-01	1.11E-01	0
835	71	recurrent	OPC	NK cell	1.17	3.68E-01	6.37E-01	3.68E-01	0
836	71	recurrent	OPC	NPC	1.08	6.79E-01	3.26E-01	3.26E-01	0
837	71	recurrent	OPC	Neuron	1.84	9.99E-04	1.00E+00	9.99E-04	1
838	71	recurrent	OPC	OPC	2.26	9.99E-04	1.00E+00	9.99E-04	1
839	71	recurrent	OPC	Oligodendrocyte	1.45	9.99E-04	1.00E+00	9.99E-04	1
840	71	recurrent	OPC	T cell	1.74	9.99E-04	1.00E+00	9.99E-04	1
841	71	recurrent	Oligodendrocyte	AC	1.25	9.45E-01	6.09E-02	6.09E-02	0
842	71	recurrent	Oligodendrocyte	Astrocyte	1.21	2.39E-01	7.62E-01	2.39E-01	0
843	71	recurrent	Oligodendrocyte	Endothelial	1.68	9.99E-04	1.00E+00	9.99E-04	1
844	71	recurrent	Oligodendrocyte	MES	1.55	1.90E-02	9.82E-01	1.90E-02	0
845	71	recurrent	Oligodendrocyte	Macrophage	1.23	4.00E-03	9.97E-01	4.00E-03	1
846	71	recurrent	Oligodendrocyte	Microglia	1.48	9.99E-04	1.00E+00	9.99E-04	1
847	71	recurrent	Oligodendrocyte	NK cell	1.15	5.27E-01	4.78E-01	4.78E-01	0
848	71	recurrent	Oligodendrocyte	NPC	1.00	1.00E+00	7.99E-03	7.99E-03	-1
849	71	recurrent	Oligodendrocyte	Neuron	1.70	9.99E-04	1.00E+00	9.99E-04	1
850	71	recurrent	Oligodendrocyte	OPC	1.59	9.99E-04	1.00E+00	9.99E-04	1
851	71	recurrent	Oligodendrocyte	Oligodendrocyte	2.09	9.99E-04	1.00E+00	9.99E-04	1
852	71	recurrent	Oligodendrocyte	T cell	1.67	1.10E-02	9.90E-01	1.10E-02	0
853	71	recurrent	T cell	AC	2.12	9.99E-04	1.00E+00	9.99E-04	1
854	71	recurrent	T cell	Astrocyte	1.37	9.99E-04	1.00E+00	9.99E-04	1
855	71	recurrent	T cell	Endothelial	1.43	9.99E-04	1.00E+00	9.99E-04	1
856	71	recurrent	T cell	MES	1.57	9.99E-04	1.00E+00	9.99E-04	1
857	71	recurrent	T cell	Macrophage	1.13	4.00E-02	9.61E-01	4.00E-02	0
858	71	recurrent	T cell	Microglia	1.37	9.99E-04	1.00E+00	9.99E-04	1
859	71	recurrent	T cell	NK cell	1.52	9.99E-04	1.00E+00	9.99E-04	1
860	71	recurrent	T cell	NPC	1.26	9.99E-04	1.00E+00	9.99E-04	1
861	71	recurrent	T cell	Neuron	1.47	9.99E-04	1.00E+00	9.99E-04	1
862	71	recurrent	T cell	OPC	1.57	9.99E-04	1.00E+00	9.99E-04	1
863	71	recurrent	T cell	Oligodendrocyte	1.49	9.99E-04	1.00E+00	9.99E-04	1
864	71	recurrent	T cell	T cell	2.74	9.99E-04	1.00E+00	9.99E-04	1
865	82	primary	AC	AC	3.30	9.99E-04	1.00E+00	9.99E-04	1
866	82	primary	AC	Astrocyte	1.03	1.71E-01	8.30E-01	1.71E-01	0
867	82	primary	AC	Endothelial	1.54	9.99E-04	1.00E+00	9.99E-04	1
868	82	primary	AC	MES	1.21	1.00E+00	9.99E-04	9.99E-04	-1
869	82	primary	AC	Macrophage	1.02	1.00E+00	9.99E-04	9.99E-04	-1
870	82	primary	AC	Microglia	1.14	8.41E-01	1.60E-01	1.60E-01	0
871	82	primary	AC	NK cell	1.06	5.02E-01	4.99E-01	4.99E-01	0
872	82	primary	AC	NPC	1.54	9.99E-04	1.00E+00	9.99E-04	1
873	82	primary	AC	Neuron	1.08	1.15E-01	8.86E-01	1.15E-01	0
874	82	primary	AC	OPC	1.39	9.99E-04	1.00E+00	9.99E-04	1
875	82	primary	AC	Oligodendrocyte	1.00	1.00E+00	8.30E-01	8.30E-01	0

row	patient	surgery	from cell type	to cell tye	observed count	permutations (greater than)	permutations (less than)	p value	interaction significance/direction
876	82	primary	AC	T cell	1.18	3.11E-01	6.90E-01	3.11E-01	0
877	82	primary	Astrocyte	AC	3.12	9.99E-04	1.00E+00	9.99E-04	1
878	82	primary	Astrocyte	Astrocyte	1.09	3.10E-02	9.70E-01	3.10E-02	0
879	82	primary	Astrocyte	Endothelial	1.58	5.99E-03	9.98E-01	5.99E-03	1
880	82	primary	Astrocyte	MES	2.67	9.99E-04	1.00E+00	9.99E-04	1
881	82	primary	Astrocyte	Macrophage	1.38	4.00E-03	9.97E-01	4.00E-03	1
882	82	primary	Astrocyte	Microglia	1.29	8.19E-02	9.24E-01	8.19E-02	0
883	82	primary	Astrocyte	NK cell	1.00	1.00E+00	6.39E-01	6.39E-01	0
884	82	primary	Astrocyte	NPC	1.74	7.99E-03	9.93E-01	7.99E-03	1
885	82	primary	Astrocyte	Neuron	1.22	5.49E-02	9.56E-01	5.49E-02	0
886	82	primary	Astrocyte	OPC	1.00	1.00E+00	6.09E-02	6.09E-02	0
887	82	primary	Astrocyte	Oligodendrocyte	1.00	6.49E-01	9.92E-01	6.49E-01	0
888	82	primary	Astrocyte	T cell	1.23	2.61E-01	7.48E-01	2.61E-01	0
889	82	primary	Endothelial	AC	2.26	9.99E-04	1.00E+00	9.99E-04	1
890	82	primary	Endothelial	Astrocyte	1.02	4.29E-01	5.85E-01	4.29E-01	0
891	82	primary	Endothelial	Endothelial	2.50	9.99E-04	1.00E+00	9.99E-04	1
892	82	primary	Endothelial	MES	2.24	9.99E-04	1.00E+00	9.99E-04	1
893	82	primary	Endothelial	Macrophage	1.13	4.00E-03	9.97E-01	4.00E-03	1
894	82	primary	Endothelial	Microglia	1.20	3.50E-02	9.66E-01	3.50E-02	0
895	82	primary	Endothelial	NK cell	1.14	9.99E-04	1.00E+00	9.99E-04	1
896	82	primary	Endothelial	NPC	1.48	5.59E-02	9.45E-01	5.59E-02	0
897	82	primary	Endothelial	Neuron	1.03	9.48E-01	5.29E-02	5.29E-02	0
898	82	primary	Endothelial	OPC	1.28	9.99E-04	1.00E+00	9.99E-04	1
899	82	primary	Endothelial	Oligodendrocyte	1.00	1.00E+00	9.05E-01	9.05E-01	0
900	82	primary	Endothelial	T cell	1.19	2.00E-01	8.03E-01	2.00E-01	0
901	82	primary	MES	AC	1.55	1.00E+00	9.99E-04	9.99E-04	-1
902	82	primary	MES	Astrocyte	1.04	7.89E-02	9.23E-01	7.89E-02	0
903	82	primary	MES	Endothelial	1.36	6.99E-03	9.94E-01	6.99E-03	1
904	82	primary	MES	MES	4.29	9.99E-04	1.00E+00	9.99E-04	1
905	82	primary	MES	Macrophage	1.22	9.99E-04	1.00E+00	9.99E-04	1
906	82	primary	MES	Microglia	1.23	9.99E-04	1.00E+00	9.99E-04	1
907	82	primary	MES	NK cell	1.10	2.30E-02	9.78E-01	2.30E-02	0
908	82	primary	MES	NPC	1.24	1.00E+00	9.99E-04	9.99E-04	-1
909	82	primary	MES	Neuron	1.03	9.83E-01	1.80E-02	1.80E-02	0
910	82	primary	MES	OPC	1.07	1.00E+00	9.99E-04	9.99E-04	-1
911	82	primary	MES	Oligodendrocyte	1.02	1.66E-01	8.35E-01	1.66E-01	0
912	82	primary	MES	T cell	1.24	9.99E-04	1.00E+00	9.99E-04	1
913	82	primary	Macrophage	AC	2.40	9.99E-04	1.00E+00	9.99E-04	1
914	82	primary	Macrophage	Astrocyte	1.00	1.00E+00	8.32E-01	8.32E-01	0
915	82	primary	Macrophage	Endothelial	1.55	9.99E-04	1.00E+00	9.99E-04	1
916	82	primary	Macrophage	MES	2.85	9.99E-04	1.00E+00	9.99E-04	1
917	82	primary	Macrophage	Macrophage	1.17	5.19E-02	9.50E-01	5.19E-02	0
918	82	primary	Macrophage	Microglia	1.42	9.99E-04	1.00E+00	9.99E-04	1
919	82	primary	Macrophage	NK cell	1.39	9.99E-04	1.00E+00	9.99E-04	1
920	82	primary	Macrophage	NPC	1.70	9.99E-04	1.00E+00	9.99E-04	1
921	82	primary	Macrophage	Neuron	1.21	5.99E-03	9.95E-01	5.99E-03	1
922	82	primary	Macrophage	OPC	1.22	6.59E-02	9.36E-01	6.59E-02	0
923	82	primary	Macrophage	Oligodendrocyte	1.00	9.77E-01	9.82E-01	9.77E-01	0
924	82	primary	Macrophage	T cell	1.28	2.00E-02	9.81E-01	2.00E-02	0
925	82	primary	Microglia	AC	2.55	9.99E-04	1.00E+00	9.99E-04	1
926	82	primary	Microglia	Astrocyte	1.05	1.72E-01	8.53E-01	1.72E-01	0
927	82	primary	Microglia	Endothelial	1.51	9.99E-04	1.00E+00	9.99E-04	1
928	82	primary	Microglia	MES	2.88	9.99E-04	1.00E+00	9.99E-04	1
929	82	primary	Microglia	Macrophage	1.30	9.99E-04	1.00E+00	9.99E-04	1
930	82	primary	Microglia	Microglia	1.30	9.99E-04	1.00E+00	9.99E-04	1
931	82	primary	Microglia	NK cell	1.28	9.99E-04	1.00E+00	9.99E-04	1
932	82	primary	Microglia	NPC	1.65	9.99E-04	1.00E+00	9.99E-04	1
933	82	primary	Microglia	Neuron	1.14	1.30E-02	9.88E-01	1.30E-02	0
934	82	primary	Microglia	OPC	1.43	9.99E-04	1.00E+00	9.99E-04	1
935	82	primary	Microglia	Oligodendrocyte	1.00	1.00E+00	9.52E-01	9.52E-01	0
936	82	primary	Microglia	T cell	1.25	1.20E-02	9.89E-01	1.20E-02	0
937	82	primary	NK cell	AC	2.44	9.99E-04	1.00E+00	9.99E-04	1
938	82	primary	NK cell	Astrocyte	1.00	1.00E+00	8.47E-01	8.47E-01	0
939	82	primary	NK cell	Endothelial	1.65	9.99E-04	1.00E+00	9.99E-04	1
940	82	primary	NK cell	MES	2.62	9.99E-04	1.00E+00	9.99E-04	1
941	82	primary	NK cell	Macrophage	1.39	9.99E-04	1.00E+00	9.99E-04	1
942	82	primary	NK cell	Microglia	1.31	3.00E-03	9.98E-01	3.00E-03	1
943	82	primary	NK cell	NK cell	1.29	1.20E-02	9.89E-01	1.20E-02	0
944	82	primary	NK cell	NPC	1.75	9.99E-04	1.00E+00	9.99E-04	1
945	82	primary	NK cell	Neuron	1.15	4.90E-02	9.59E-01	4.90E-02	0
946	82	primary	NK cell	OPC	1.30	9.99E-03	9.91E-01	9.99E-03	1
947	82	primary	NK cell	Oligodendrocyte	1.00	9.39E-01	9.81E-01	9.39E-01	0
948	82	primary	NK cell	T cell	1.42	9.99E-04	1.00E+00	9.99E-04	1
949	82	primary	NPC	AC	2.68	9.99E-04	1.00E+00	9.99E-04	1
950	82	primary	NPC	Astrocyte	1.14	9.99E-04	1.00E+00	9.99E-04	1
951	82	primary	NPC	Endothelial	1.59	9.99E-04	1.00E+00	9.99E-04	1
952	82	primary	NPC	MES	3.15	9.99E-04	1.00E+00	9.99E-04	1
953	82	primary	NPC	Macrophage	1.16	9.99E-04	1.00E+00	9.99E-04	1
954	82	primary	NPC	Microglia	1.23	2.00E-03	9.99E-01	2.00E-03	1
955	82	primary	NPC	NK cell	1.08	8.19E-02	9.20E-01	8.19E-02	0
956	82	primary	NPC	NPC	2.14	9.99E-04	1.00E+00	9.99E-04	1
957	82	primary	NPC	Neuron	1.23	9.99E-04	1.00E+00	9.99E-04	1
958	82	primary	NPC	OPC	1.48	9.99E-04	1.00E+00	9.99E-04	1
959	82	primary	NPC	Oligodendrocyte	1.00	1.00E+00	8.92E-01	8.92E-01	0
960	82	primary	NPC	T cell	1.21	2.80E-02	9.73E-01	2.80E-02	0
961	82	primary	Neuron	AC	2.68	9.99E-04	1.00E+00	9.99E-04	1
962	82	primary	Neuron	Astrocyte	1.00	1.00E+00	8.65E-01	8.65E-01	0
963	82	primary	Neuron	Endothelial	1.40	6.79E-02	9.34E-01	6.79E-02	0
964	82	primary	Neuron	MES	2.78	9.99E-04	1.00E+00	9.99E-04	1
965	82	primary	Neuron	Macrophage	1.21	8.99E-03	9.92E-01	8.99E-03	1
966	82	primary	Neuron	Microglia	1.30	5.99E-03	9.95E-01	5.99E-03	1
967	82	primary	Neuron	NK cell	1.11	1.33E-01	8.76E-01	1.33E-01	0
968	82	primary	Neuron	NPC	1.90	9.99E-04	1.00E+00	9.99E-04	1
969	82	primary	Neuron	Neuron	1.37	3.00E-03	9.98E-01	3.00E-03	1
970	82	primary	Neuron	OPC	1.35	9.99E-04	1.00E+00	9.99E-04	1
971	82	primary	Neuron	Oligodendrocyte	1.00	9.47E-01	9.80E-01	9.47E-01	0
972	82	primary	Neuron	T cell	1.29	2.00E-02	9.81E-01	2.00E-02	0
973	82	primary	OPC	AC	2.61	9.99E-04	1.00E+00	9.99E-04	1
974	82	primary	OPC	Astrocyte	1.00	1.00E+00	6.84E-01	6.84E-01	0
975	82	primary	OPC	Endothelial	1.46	9.99E-04	1.00E+00	9.99E-04	1
976	82	primary	OPC	MES	2.67	9.99E-04	1.00E+00	9.99E-04	1
977	82	primary	OPC	Macrophage	1.22	9.99E-04	1.00E+00	9.99E-04	1
978	82	primary	OPC	Microglia	1.13	8.06E-01	2.03E-01	2.03E-01	0
979	82	primary	OPC	NK cell	1.07	3.44E-01	6.71E-01	3.44E-01	0
980	82	primary	OPC	NPC	1.67	9.99E-04	1.00E+00	9.99E-04	1
981	82	primary	OPC	Neuron	1.19	3.00E-03	9.98E-01	3.00E-03	1
982	82	primary	OPC	OPC	1.95	9.99E-04	1.00E+00	9.99E-04	1
983	82	primary	OPC	Oligodendrocyte	1.00	1.00E+00	9.55E-01	9.55E-01	0
984	82	primary	OPC	T cell	1.19	2.83E-01	7.19E-01	2.83E-01	0
985	82	primary	Oligodendrocyte	AC	1.88	6.42E-01	3.61E-01	3.61E-01	0
986	82	primary	Oligodendrocyte	Astrocyte	1.00	6.49E-01	9.89E-01	6.49E-01	0

row	patient	surgery	from cell type	to cell tye	observed count	permutations (greater than)	permutations (less than)	p value	interaction significance/direction
987	82	primary	Oligodendrocyte	Endothelial	1.29	5.48E-01	4.80E-01	4.80E-01	0
988	82	primary	Oligodendrocyte	MES	3.27	9.99E-04	1.00E+00	9.99E-04	1
989	82	primary	Oligodendrocyte	Macrophage	1.20	1.80E-01	8.51E-01	1.80E-01	0
990	82	primary	Oligodendrocyte	Microglia	1.11	5.83E-01	4.64E-01	4.64E-01	0
991	82	primary	Oligodendrocyte	NK cell	1.00	9.39E-01	8.66E-01	8.66E-01	0
992	82	primary	Oligodendrocyte	NPC	1.55	2.71E-01	7.40E-01	2.71E-01	0
993	82	primary	Oligodendrocyte	Neuron	1.00	9.47E-01	8.63E-01	8.63E-01	0
994	82	primary	Oligodendrocyte	OPC	1.40	7.59E-02	9.39E-01	7.59E-02	0
995	82	primary	Oligodendrocyte	Oligodendrocyte	0.00	1.00E+00	8.40E-01	8.40E-01	0
996	82	primary	Oligodendrocyte	T cell	1.33	1.83E-01	8.74E-01	1.83E-01	0
997	82	primary	T cell	AC	2.62	9.99E-04	1.00E+00	9.99E-04	1
998	82	primary	T cell	Astrocyte	1.04	2.29E-01	7.92E-01	2.29E-01	0
999	82	primary	T cell	Endothelial	1.59	9.99E-04	1.00E+00	9.99E-04	1
1000	82	primary	T cell	MES	3.27	9.99E-04	1.00E+00	9.99E-04	1
1001	82	primary	T cell	Macrophage	1.14	1.20E-02	9.89E-01	1.20E-02	0
1002	82	primary	T cell	Microglia	1.40	9.99E-04	1.00E+00	9.99E-04	1
1003	82	primary	T cell	NK cell	1.25	9.99E-04	1.00E+00	9.99E-04	1
1004	82	primary	T cell	NPC	1.64	9.99E-04	1.00E+00	9.99E-04	1
1005	82	primary	T cell	Neuron	1.08	2.91E-01	7.19E-01	2.91E-01	0
1006	82	primary	T cell	OPC	1.56	9.99E-04	1.00E+00	9.99E-04	1
1007	82	primary	T cell	Oligodendrocyte	1.00	9.99E-01	9.61E-01	9.61E-01	0
1008	82	primary	T cell	T cell	1.43	9.99E-04	1.00E+00	9.99E-04	1
1009	82	recurrent	AC	AC	1.00	2.48E-01	9.93E-01	2.48E-01	0
1010	82	recurrent	AC	Astrocyte	2.31	4.00E-03	9.97E-01	4.00E-03	1
1011	82	recurrent	AC	Endothelial	1.25	1.92E-01	8.57E-01	1.92E-01	0
1012	82	recurrent	AC	MES	1.00	3.90E-01	9.97E-01	3.90E-01	0
1013	82	recurrent	AC	Macrophage	1.00	9.56E-01	8.94E-01	8.94E-01	0
1014	82	recurrent	AC	Microglia	1.00	9.90E-01	7.73E-01	7.73E-01	0
1015	82	recurrent	AC	NK cell	1.00	1.00E+00	6.77E-01	6.77E-01	0
1016	82	recurrent	AC	NPC	1.00	5.51E-01	9.93E-01	5.51E-01	0
1017	82	recurrent	AC	Neuron	2.67	5.98E-01	4.18E-01	4.18E-01	0
1018	82	recurrent	AC	OPC	0.00	1.00E+00	5.09E-02	5.09E-02	0
1019	82	recurrent	AC	Oligodendrocyte	1.36	1.28E-01	8.80E-01	1.28E-01	0
1020	82	recurrent	AC	T cell	1.20	8.06E-01	2.11E-01	2.11E-01	0
1021	82	recurrent	Astrocyte	AC	1.03	5.00E-02	9.51E-01	5.00E-02	0
1022	82	recurrent	Astrocyte	Astrocyte	2.48	9.99E-04	1.00E+00	9.99E-04	1
1023	82	recurrent	Astrocyte	Endothelial	1.26	9.99E-04	1.00E+00	9.99E-04	1
1024	82	recurrent	Astrocyte	MES	1.02	1.61E-01	8.41E-01	1.61E-01	0
1025	82	recurrent	Astrocyte	Macrophage	1.08	8.99E-03	9.92E-01	8.99E-03	1
1026	82	recurrent	Astrocyte	Microglia	1.09	6.69E-02	9.34E-01	6.69E-02	0
1027	82	recurrent	Astrocyte	NK cell	1.12	5.99E-03	9.95E-01	5.99E-03	1
1028	82	recurrent	Astrocyte	NPC	1.04	5.59E-02	9.46E-01	5.59E-02	0
1029	82	recurrent	Astrocyte	Neuron	2.63	1.00E+00	9.99E-04	9.99E-04	-1
1030	82	recurrent	Astrocyte	OPC	1.08	9.99E-04	1.00E+00	9.99E-04	1
1031	82	recurrent	Astrocyte	Oligodendrocyte	1.28	9.99E-04	1.00E+00	9.99E-04	1
1032	82	recurrent	Astrocyte	T cell	1.37	2.00E-03	9.99E-01	2.00E-03	1
1033	82	recurrent	Endothelial	AC	1.00	1.00E+00	9.24E-01	9.24E-01	0
1034	82	recurrent	Endothelial	Astrocyte	2.01	9.99E-04	1.00E+00	9.99E-04	1
1035	82	recurrent	Endothelial	Endothelial	1.96	9.99E-04	1.00E+00	9.99E-04	1
1036	82	recurrent	Endothelial	MES	1.00	1.00E+00	9.59E-01	9.59E-01	0
1037	82	recurrent	Endothelial	Macrophage	1.17	9.99E-04	1.00E+00	9.99E-04	1
1038	82	recurrent	Endothelial	Microglia	1.11	7.09E-02	9.32E-01	7.09E-02	0
1039	82	recurrent	Endothelial	NK cell	1.22	9.99E-04	1.00E+00	9.99E-04	1
1040	82	recurrent	Endothelial	NPC	1.00	1.00E+00	8.75E-01	8.75E-01	0
1041	82	recurrent	Endothelial	Neuron	2.37	1.00E+00	9.99E-04	9.99E-04	-1
1042	82	recurrent	Endothelial	OPC	1.05	2.71E-01	7.43E-01	2.71E-01	0
1043	82	recurrent	Endothelial	Oligodendrocyte	1.31	9.99E-04	1.00E+00	9.99E-04	1
1044	82	recurrent	Endothelial	T cell	1.52	9.99E-04	1.00E+00	9.99E-04	1
1045	82	recurrent	MES	AC	1.00	3.90E-01	9.97E-01	3.90E-01	0
1046	82	recurrent	MES	Astrocyte	2.43	2.00E-03	9.99E-01	2.00E-03	1
1047	82	recurrent	MES	Endothelial	1.00	1.00E+00	4.05E-01	4.05E-01	0
1048	82	recurrent	MES	MES	1.00	1.51E-01	1.00E+00	1.51E-01	0
1049	82	recurrent	MES	Macrophage	1.00	8.94E-01	9.31E-01	8.94E-01	0
1050	82	recurrent	MES	Microglia	1.00	9.75E-01	7.93E-01	7.93E-01	0
1051	82	recurrent	MES	NK cell	1.00	9.96E-01	7.18E-01	7.18E-01	0
1052	82	recurrent	MES	NPC	0.00	1.00E+00	5.13E-01	5.13E-01	0
1053	82	recurrent	MES	Neuron	2.10	9.96E-01	5.00E-03	5.00E-03	-1
1054	82	recurrent	MES	OPC	0.00	1.00E+00	1.13E-01	1.13E-01	0
1055	82	recurrent	MES	Oligodendrocyte	1.20	5.11E-01	5.55E-01	5.11E-01	0
1056	82	recurrent	MES	T cell	1.54	1.01E-01	9.08E-01	1.01E-01	0
1057	82	recurrent	Macrophage	AC	1.33	5.99E-03	9.99E-01	5.99E-03	1
1058	82	recurrent	Macrophage	Astrocyte	1.71	7.74E-01	2.27E-01	2.27E-01	0
1059	82	recurrent	Macrophage	Endothelial	1.36	9.99E-04	1.00E+00	9.99E-04	1
1060	82	recurrent	Macrophage	MES	1.00	8.94E-01	9.90E-01	8.94E-01	0
1061	82	recurrent	Macrophage	Macrophage	1.14	8.69E-02	9.30E-01	8.69E-02	0
1062	82	recurrent	Macrophage	Microglia	1.35	2.00E-03	9.99E-01	2.00E-03	1
1063	82	recurrent	Macrophage	NK cell	1.21	2.00E-02	9.81E-01	2.00E-02	0
1064	82	recurrent	Macrophage	NPC	1.25	1.70E-02	9.90E-01	1.70E-02	0
1065	82	recurrent	Macrophage	Neuron	2.81	2.05E-01	7.96E-01	2.05E-01	0
1066	82	recurrent	Macrophage	OPC	1.00	1.00E+00	6.54E-01	6.54E-01	0
1067	82	recurrent	Macrophage	Oligodendrocyte	1.48	9.99E-04	1.00E+00	9.99E-04	1
1068	82	recurrent	Macrophage	T cell	1.80	9.99E-04	1.00E+00	9.99E-04	1
1069	82	recurrent	Microglia	AC	1.00	9.90E-01	9.65E-01	9.65E-01	0
1070	82	recurrent	Microglia	Astrocyte	1.82	1.31E-01	8.70E-01	1.31E-01	0
1071	82	recurrent	Microglia	Endothelial	1.27	7.99E-03	9.93E-01	7.99E-03	1
1072	82	recurrent	Microglia	MES	1.33	5.99E-03	9.99E-01	5.99E-03	1
1073	82	recurrent	Microglia	Macrophage	1.11	6.89E-02	9.38E-01	6.89E-02	0
1074	82	recurrent	Microglia	Microglia	1.39	9.99E-04	1.00E+00	9.99E-04	1
1075	82	recurrent	Microglia	NK cell	1.44	9.99E-04	1.00E+00	9.99E-04	1
1076	82	recurrent	Microglia	NPC	1.00	9.99E-01	9.43E-01	9.43E-01	0
1077	82	recurrent	Microglia	Neuron	2.63	9.27E-01	7.39E-02	7.39E-02	0
1078	82	recurrent	Microglia	OPC	1.11	6.39E-02	9.47E-01	6.39E-02	0
1079	82	recurrent	Microglia	Oligodendrocyte	1.37	9.99E-04	1.00E+00	9.99E-04	1
1080	82	recurrent	Microglia	T cell	1.65	9.99E-04	1.00E+00	9.99E-04	1
1081	82	recurrent	NK cell	AC	1.00	1.00E+00	9.58E-01	9.58E-01	0
1082	82	recurrent	NK cell	Astrocyte	1.63	9.95E-01	5.99E-03	5.99E-03	-1
1083	82	recurrent	NK cell	Endothelial	1.40	9.99E-04	1.00E+00	9.99E-04	1
1084	82	recurrent	NK cell	MES	1.00	9.96E-01	9.82E-01	9.82E-01	0
1085	82	recurrent	NK cell	Macrophage	1.09	8.69E-02	9.14E-01	8.69E-02	0
1086	82	recurrent	NK cell	Microglia	1.32	9.99E-04	1.00E+00	9.99E-04	1
1087	82	recurrent	NK cell	NK cell	1.46	9.99E-04	1.00E+00	9.99E-04	1
1088	82	recurrent	NK cell	NPC	1.00	1.00E+00	9.16E-01	9.16E-01	0
1089	82	recurrent	NK cell	Neuron	2.26	1.00E+00	9.99E-04	9.99E-04	-1
1090	82	recurrent	NK cell	OPC	1.33	9.99E-04	1.00E+00	9.99E-04	1
1091	82	recurrent	NK cell	Oligodendrocyte	1.40	9.99E-04	1.00E+00	9.99E-04	1
1092	82	recurrent	NK cell	T cell	1.91	9.99E-04	1.00E+00	9.99E-04	1
1093	82	recurrent	NPC	AC	1.00	5.51E-01	9.94E-01	5.51E-01	0
1094	82	recurrent	NPC	Astrocyte	1.81	3.64E-01	6.38E-01	3.64E-01	0
1095	82	recurrent	NPC	Endothelial	1.30	8.99E-02	9.18E-01	8.99E-02	0
1096	82	recurrent	NPC	MES	0.00	1.00E+00	5.13E-01	5.13E-01	0
1097	82	recurrent	NPC	Macrophage	1.00	9.81E-01	8.56E-01	8.56E-01	0

row	patient	surgery	from cell type	to cell tye	observed count	permutations (greater than)	permutations (less than)	p value	interaction significance/direction
1098	82	recurrent	NPC	Microglia	1.00	9.99E-01	6.86E-01	6.86E-01	0
1099	82	recurrent	NPC	NK cell	1.20	1.52E-01	8.94E-01	1.52E-01	0
1100	82	recurrent	NPC	NPC	2.00	3.00E-03	1.00E+00	3.00E-03	1
1101	82	recurrent	NPC	Neuron	2.74	4.86E-01	5.26E-01	4.86E-01	0
1102	82	recurrent	NPC	OPC	1.33	3.60E-02	9.81E-01	3.60E-02	0
1103	82	recurrent	NPC	Oligodendrocyte	1.33	1.50E-01	8.86E-01	1.50E-01	0
1104	82	recurrent	NPC	T cell	1.75	2.00E-03	9.99E-01	2.00E-03	1
1105	82	recurrent	Neuron	AC	1.00	1.00E+00	5.69E-01	5.69E-01	0
1106	82	recurrent	Neuron	Astrocyte	1.99	9.99E-04	1.00E+00	9.99E-04	1
1107	82	recurrent	Neuron	Endothelial	1.25	9.99E-04	1.00E+00	9.99E-04	1
1108	82	recurrent	Neuron	MES	1.07	2.00E-03	9.99E-01	2.00E-03	1
1109	82	recurrent	Neuron	Macrophage	1.04	4.66E-01	5.37E-01	4.66E-01	0
1110	82	recurrent	Neuron	Microglia	1.07	2.23E-01	7.80E-01	2.23E-01	0
1111	82	recurrent	Neuron	NK cell	1.23	9.99E-04	1.00E+00	9.99E-04	1
1112	82	recurrent	Neuron	NPC	1.02	2.36E-01	7.76E-01	2.36E-01	0
1113	82	recurrent	Neuron	Neuron	3.17	9.99E-04	1.00E+00	9.99E-04	1
1114	82	recurrent	Neuron	OPC	1.12	9.99E-04	1.00E+00	9.99E-04	1
1115	82	recurrent	Neuron	Oligodendrocyte	1.37	9.99E-04	1.00E+00	9.99E-04	1
1116	82	recurrent	Neuron	T cell	1.52	9.99E-04	1.00E+00	9.99E-04	1
1117	82	recurrent	OPC	AC	0.00	1.00E+00	5.09E-02	5.09E-02	0
1118	82	recurrent	OPC	Astrocyte	2.31	9.99E-04	1.00E+00	9.99E-04	1
1119	82	recurrent	OPC	Endothelial	1.15	3.97E-01	6.23E-01	3.97E-01	0
1120	82	recurrent	OPC	MES	0.00	1.00E+00	1.13E-01	1.13E-01	0
1121	82	recurrent	OPC	Macrophage	1.00	1.00E+00	6.12E-01	6.12E-01	0
1122	82	recurrent	OPC	Microglia	1.11	2.02E-01	8.16E-01	2.02E-01	0
1123	82	recurrent	OPC	NK cell	1.33	4.00E-03	9.98E-01	4.00E-03	1
1124	82	recurrent	OPC	NPC	1.00	9.79E-01	9.72E-01	9.72E-01	0
1125	82	recurrent	OPC	Neuron	3.11	9.99E-04	1.00E+00	9.99E-04	1
1126	82	recurrent	OPC	OPC	1.12	8.49E-02	9.16E-01	8.49E-02	0
1127	82	recurrent	OPC	Oligodendrocyte	1.37	1.40E-02	9.87E-01	1.40E-02	0
1128	82	recurrent	OPC	T cell	1.21	9.52E-01	4.90E-02	4.90E-02	0
1129	82	recurrent	Oligodendrocyte	AC	1.00	1.00E+00	8.92E-01	8.92E-01	0
1130	82	recurrent	Oligodendrocyte	Astrocyte	1.86	2.00E-03	9.99E-01	2.00E-03	1
1131	82	recurrent	Oligodendrocyte	Endothelial	1.38	9.99E-04	1.00E+00	9.99E-04	1
1132	82	recurrent	Oligodendrocyte	MES	1.09	2.80E-02	9.79E-01	2.80E-02	0
1133	82	recurrent	Oligodendrocyte	Macrophage	1.01	8.81E-01	1.29E-01	1.29E-01	0
1134	82	recurrent	Oligodendrocyte	Microglia	1.18	9.99E-04	1.00E+00	9.99E-04	1
1135	82	recurrent	Oligodendrocyte	NK cell	1.29	9.99E-04	1.00E+00	9.99E-04	1
1136	82	recurrent	Oligodendrocyte	NPC	1.04	1.44E-01	8.72E-01	1.44E-01	0
1137	82	recurrent	Oligodendrocyte	Neuron	2.79	1.01E-01	9.00E-01	1.01E-01	0
1138	82	recurrent	Oligodendrocyte	OPC	1.10	5.00E-03	9.96E-01	5.00E-03	1
1139	82	recurrent	Oligodendrocyte	Oligodendrocyte	1.68	9.99E-04	1.00E+00	9.99E-04	1
1140	82	recurrent	Oligodendrocyte	T cell	1.48	9.99E-04	1.00E+00	9.99E-04	1
1141	82	recurrent	T cell	AC	1.00	1.00E+00	8.61E-01	8.61E-01	0
1142	82	recurrent	T cell	Astrocyte	1.77	4.01E-01	6.00E-01	4.01E-01	0
1143	82	recurrent	T cell	Endothelial	1.31	9.99E-04	1.00E+00	9.99E-04	1
1144	82	recurrent	T cell	MES	1.00	1.00E+00	9.24E-01	9.24E-01	0
1145	82	recurrent	T cell	Macrophage	1.07	7.19E-02	9.32E-01	7.19E-02	0
1146	82	recurrent	T cell	Microglia	1.19	9.99E-04	1.00E+00	9.99E-04	1
1147	82	recurrent	T cell	NK cell	1.30	9.99E-04	1.00E+00	9.99E-04	1
1148	82	recurrent	T cell	NPC	1.00	1.00E+00	7.44E-01	7.44E-01	0
1149	82	recurrent	T cell	Neuron	2.58	1.00E+00	9.99E-04	9.99E-04	-1
1150	82	recurrent	T cell	OPC	1.08	1.10E-02	9.91E-01	1.10E-02	0
1151	82	recurrent	T cell	Oligodendrocyte	1.32	9.99E-04	1.00E+00	9.99E-04	1
1152	82	recurrent	T cell	T cell	1.95	9.99E-04	1.00E+00	9.99E-04	1
1153	84	primary	AC	AC	0.00	1.00E+00	9.98E-01	9.98E-01	0
1154	84	primary	AC	Astrocyte	3.67	8.19E-02	9.61E-01	8.19E-02	0
1155	84	primary	AC	Endothelial	1.00	7.88E-01	7.68E-01	7.68E-01	0
1156	84	primary	AC	MES	1.50	9.23E-01	1.20E-01	1.20E-01	0
1157	84	primary	AC	Macrophage	3.00	2.00E-03	1.00E+00	2.00E-03	1
1158	84	primary	AC	Microglia	0.00	1.00E+00	6.12E-01	6.12E-01	0
1159	84	primary	AC	NK cell	1.00	5.07E-01	9.45E-01	5.07E-01	0
1160	84	primary	AC	NPC	0.00	1.00E+00	9.77E-01	9.77E-01	0
1161	84	primary	AC	Neuron	0.00	1.00E+00	8.60E-01	8.60E-01	0
1162	84	primary	AC	OPC	0.00	1.00E+00	9.69E-01	9.69E-01	0
1163	84	primary	AC	Oligodendrocyte					
1164	84	primary	AC	T cell	0.00	1.00E+00	6.97E-01	6.97E-01	0
1165	84	primary	Astrocyte	AC	1.00	1.00E+00	9.97E-01	9.97E-01	0
1166	84	primary	Astrocyte	Astrocyte	3.81	9.99E-04	1.00E+00	9.99E-04	1
1167	84	primary	Astrocyte	Endothelial	1.42	9.99E-04	1.00E+00	9.99E-04	1
1168	84	primary	Astrocyte	MES	2.32	9.96E-01	5.00E-03	5.00E-03	-1
1169	84	primary	Astrocyte	Macrophage	1.19	9.99E-04	1.00E+00	9.99E-04	1
1170	84	primary	Astrocyte	Microglia	1.11	2.00E-03	9.99E-01	2.00E-03	1
1171	84	primary	Astrocyte	NK cell	1.35	9.99E-04	1.00E+00	9.99E-04	1
1172	84	primary	Astrocyte	NPC	1.00	1.00E+00	9.19E-01	9.19E-01	0
1173	84	primary	Astrocyte	Neuron	1.10	9.99E-04	1.00E+00	9.99E-04	1
1174	84	primary	Astrocyte	OPC	1.07	3.00E-03	9.98E-01	3.00E-03	1
1175	84	primary	Astrocyte	Oligodendrocyte					
1176	84	primary	Astrocyte	T cell	1.11	9.99E-04	1.00E+00	9.99E-04	1
1177	84	primary	Endothelial	AC	1.00	7.88E-01	1.00E+00	7.88E-01	0
1178	84	primary	Endothelial	Astrocyte	2.49	9.39E-01	6.19E-02	6.19E-02	0
1179	84	primary	Endothelial	Endothelial	2.24	9.99E-04	1.00E+00	9.99E-04	1
1180	84	primary	Endothelial	MES	2.59	9.99E-04	1.00E+00	9.99E-04	1
1181	84	primary	Endothelial	Macrophage	1.13	1.53E-01	8.48E-01	1.53E-01	0
1182	84	primary	Endothelial	Microglia	1.16	2.00E-03	9.99E-01	2.00E-03	1
1183	84	primary	Endothelial	NK cell	1.49	9.99E-04	1.00E+00	9.99E-04	1
1184	84	primary	Endothelial	NPC	1.00	9.99E-01	9.76E-01	9.76E-01	0
1185	84	primary	Endothelial	Neuron	1.10	1.60E-02	9.85E-01	1.60E-02	0
1186	84	primary	Endothelial	OPC	1.00	1.00E+00	9.54E-01	9.54E-01	0
1187	84	primary	Endothelial	Oligodendrocyte					
1188	84	primary	Endothelial	T cell	1.16	2.00E-03	9.99E-01	2.00E-03	1
1189	84	primary	MES	AC	1.00	9.99E-01	9.95E-01	9.95E-01	0
1190	84	primary	MES	Astrocyte	2.18	1.00E+00	9.99E-04	9.99E-04	-1
1191	84	primary	MES	Endothelial	1.35	9.99E-04	1.00E+00	9.99E-04	1
1192	84	primary	MES	MES	3.97	9.99E-04	1.00E+00	9.99E-04	1
1193	84	primary	MES	Macrophage	1.15	9.99E-04	1.00E+00	9.99E-04	1
1194	84	primary	MES	Microglia	1.12	9.99E-04	1.00E+00	9.99E-04	1
1195	84	primary	MES	NK cell	1.24	9.99E-04	1.00E+00	9.99E-04	1
1196	84	primary	MES	NPC	1.03	6.49E-02	9.44E-01	6.49E-02	0
1197	84	primary	MES	Neuron	1.06	5.99E-03	9.95E-01	5.99E-03	1
1198	84	primary	MES	OPC	1.03	5.09E-02	9.54E-01	5.09E-02	0
1199	84	primary	MES	Oligodendrocyte					
1200	84	primary	MES	T cell	1.14	9.99E-04	1.00E+00	9.99E-04	1
1201	84	primary	Macrophage	AC	1.00	5.10E-01	9.99E-01	5.10E-01	0
1202	84	primary	Macrophage	Astrocyte	3.44	9.99E-04	1.00E+00	9.99E-04	1
1203	84	primary	Macrophage	Endothelial	1.54	9.99E-04	1.00E+00	9.99E-04	1
1204	84	primary	Macrophage	MES	2.19	9.98E-01	3.00E-03	3.00E-03	-1
1205	84	primary	Macrophage	Macrophage	1.43	9.99E-04	1.00E+00	9.99E-04	1
1206	84	primary	Macrophage	Microglia	1.27	9.99E-04	1.00E+00	9.99E-04	1
1207	84	primary	Macrophage	NK cell	1.66	9.99E-04	1.00E+00	9.99E-04	1
1208	84	primary	Macrophage	NPC	1.00	9.65E-01	9.91E-01	9.65E-01	0

row	patient	surgery	from cell type	to cell tye	observed count	permutations (greater than)	permutations (less than)	p value	interaction significance/direction
1209	84	primary	Macrophage	Neuron	1.17	3.00E-03	9.98E-01	3.00E-03	1
1210	84	primary	Macrophage	OPC	1.00	9.91E-01	9.81E-01	9.81E-01	0
1211	84	primary	Macrophage	Oligodendrocyte					
1212	84	primary	Macrophage	T cell	1.13	2.90E-02	9.73E-01	2.90E-02	0
1213	84	primary	Microglia	AC	0.00	1.00E+00	6.12E-01	6.12E-01	0
1214	84	primary	Microglia	Astrocyte	3.30	9.99E-04	1.00E+00	9.99E-04	1
1215	84	primary	Microglia	Endothelial	1.58	9.99E-04	1.00E+00	9.99E-04	1
1216	84	primary	Microglia	MES	2.48	2.80E-02	9.73E-01	2.80E-02	0
1217	84	primary	Microglia	Macrophage	1.34	9.99E-04	1.00E+00	9.99E-04	1
1218	84	primary	Microglia	Microglia	1.20	2.50E-02	9.76E-01	2.50E-02	0
1219	84	primary	Microglia	NK cell	1.59	9.99E-04	1.00E+00	9.99E-04	1
1220	84	primary	Microglia	NPC	1.00	8.83E-01	9.96E-01	8.83E-01	0
1221	84	primary	Microglia	Neuron	1.07	1.60E-01	8.66E-01	1.60E-01	0
1222	84	primary	Microglia	OPC	0.00	1.00E+00	3.40E-02	3.40E-02	0
1223	84	primary	Microglia	Oligodendrocyte					
1224	84	primary	Microglia	T cell	1.29	9.99E-04	1.00E+00	9.99E-04	1
1225	84	primary	NK cell	AC	1.00	5.07E-01	9.99E-01	5.07E-01	0
1226	84	primary	NK cell	Astrocyte	2.38	9.97E-01	4.00E-03	4.00E-03	-1
1227	84	primary	NK cell	Endothelial	1.63	9.99E-04	1.00E+00	9.99E-04	1
1228	84	primary	NK cell	MES	2.46	3.70E-02	9.64E-01	3.70E-02	0
1229	84	primary	NK cell	Macrophage	1.56	9.99E-04	1.00E+00	9.99E-04	1
1230	84	primary	NK cell	Microglia	1.25	9.99E-04	1.00E+00	9.99E-04	1
1231	84	primary	NK cell	NK cell	1.93	9.99E-04	1.00E+00	9.99E-04	1
1232	84	primary	NK cell	NPC	1.00	9.69E-01	9.87E-01	9.69E-01	0
1233	84	primary	NK cell	Neuron	1.00	1.00E+00	7.00E-01	7.00E-01	0
1234	84	primary	NK cell	OPC	1.00	9.85E-01	9.76E-01	9.76E-01	0
1235	84	primary	NK cell	Oligodendrocyte					
1236	84	primary	NK cell	T cell	1.12	3.90E-02	9.62E-01	3.90E-02	0
1237	84	primary	NPC	AC	0.00	1.00E+00	9.77E-01	9.77E-01	0
1238	84	primary	NPC	Astrocyte	2.54	4.96E-01	5.31E-01	4.96E-01	0
1239	84	primary	NPC	Endothelial	1.00	9.99E-01	3.09E-01	3.09E-01	0
1240	84	primary	NPC	MES	2.83	7.59E-02	9.32E-01	7.59E-02	0
1241	84	primary	NPC	Macrophage	1.00	9.65E-01	7.68E-01	7.68E-01	0
1242	84	primary	NPC	Microglia	1.00	8.83E-01	8.72E-01	8.72E-01	0
1243	84	primary	NPC	NK cell	1.00	9.69E-01	7.28E-01	7.28E-01	0
1244	84	primary	NPC	NPC	0.00	1.00E+00	9.54E-01	9.54E-01	0
1245	84	primary	NPC	Neuron	0.00	1.00E+00	4.77E-01	4.77E-01	0
1246	84	primary	NPC	OPC	0.00	1.00E+00	8.71E-01	8.71E-01	0
1247	84	primary	NPC	Oligodendrocyte					
1248	84	primary	NPC	T cell	0.00	1.00E+00	2.10E-01	2.10E-01	0
1249	84	primary	Neuron	AC	0.00	1.00E+00	8.60E-01	8.60E-01	0
1250	84	primary	Neuron	Astrocyte	4.20	9.99E-04	1.00E+00	9.99E-04	1
1251	84	primary	Neuron	Endothelial	1.44	2.20E-02	9.80E-01	2.20E-02	0
1252	84	primary	Neuron	MES	2.12	9.75E-01	2.60E-02	2.60E-02	0
1253	84	primary	Neuron	Macrophage	1.04	8.17E-01	1.84E-01	1.84E-01	0
1254	84	primary	Neuron	Microglia	1.00	1.00E+00	4.24E-01	4.24E-01	0
1255	84	primary	Neuron	NK cell	1.00	1.00E+00	1.66E-01	1.66E-01	0
1256	84	primary	Neuron	NPC	0.00	1.00E+00	4.77E-01	4.77E-01	0
1257	84	primary	Neuron	Neuron	1.19	3.80E-02	9.63E-01	3.80E-02	0
1258	84	primary	Neuron	OPC	1.00	5.80E-01	9.98E-01	5.80E-01	0
1259	84	primary	Neuron	Oligodendrocyte					
1260	84	primary	Neuron	T cell	1.00	1.00E+00	6.40E-01	6.40E-01	0
1261	84	primary	OPC	AC	0.00	1.00E+00	9.69E-01	9.69E-01	0
1262	84	primary	OPC	Astrocyte	2.81	1.63E-01	8.43E-01	1.63E-01	0
1263	84	primary	OPC	Endothelial	1.00	1.00E+00	1.79E-01	1.79E-01	0
1264	84	primary	OPC	MES	3.08	9.99E-03	9.91E-01	9.99E-03	1
1265	84	primary	OPC	Macrophage	1.00	9.91E-01	6.71E-01	6.71E-01	0
1266	84	primary	OPC	Microglia	0.00	1.00E+00	3.40E-02	3.40E-02	0
1267	84	primary	OPC	NK cell	2.00	1.30E-02	9.99E-01	1.30E-02	0
1268	84	primary	OPC	NPC	0.00	1.00E+00	8.71E-01	8.71E-01	0
1269	84	primary	OPC	Neuron	1.33	1.60E-02	9.86E-01	1.60E-02	0
1270	84	primary	OPC	OPC	1.00	1.01E-01	9.99E-01	1.01E-01	0
1271	84	primary	OPC	Oligodendrocyte					
1272	84	primary	OPC	T cell	1.00	8.67E-01	9.12E-01	8.67E-01	0
1273	84	primary	Oligodendrocyte	AC					
1274	84	primary	Oligodendrocyte	Astrocyte					
1275	84	primary	Oligodendrocyte	Endothelial					
1276	84	primary	Oligodendrocyte	MES					
1277	84	primary	Oligodendrocyte	Macrophage					
1278	84	primary	Oligodendrocyte	Microglia					
1279	84	primary	Oligodendrocyte	NK cell					
1280	84	primary	Oligodendrocyte	NPC					
1281	84	primary	Oligodendrocyte	Neuron					
1282	84	primary	Oligodendrocyte	OPC					
1283	84	primary	Oligodendrocyte	Oligodendrocyte					
1284	84	primary	Oligodendrocyte	T cell					
1285	84	primary	T cell	AC	0.00	1.00E+00	6.97E-01	6.97E-01	0
1286	84	primary	T cell	Astrocyte	2.48	8.18E-01	1.83E-01	1.83E-01	0
1287	84	primary	T cell	Endothelial	1.47	9.99E-04	1.00E+00	9.99E-04	1
1288	84	primary	T cell	MES	3.07	9.99E-04	1.00E+00	9.99E-04	1
1289	84	primary	T cell	Macrophage	1.44	9.99E-04	1.00E+00	9.99E-04	1
1290	84	primary	T cell	Microglia	1.20	1.20E-02	9.89E-01	1.20E-02	0
1291	84	primary	T cell	NK cell	1.68	9.99E-04	1.00E+00	9.99E-04	1
1292	84	primary	T cell	NPC	0.00	1.00E+00	2.10E-01	2.10E-01	0
1293	84	primary	T cell	Neuron	1.00	1.00E+00	8.46E-01	8.46E-01	0
1294	84	primary	T cell	OPC	1.00	8.67E-01	9.92E-01	8.67E-01	0
1295	84	primary	T cell	Oligodendrocyte					
1296	84	primary	T cell	T cell	1.23	2.30E-02	9.81E-01	2.30E-02	0
1297	84	recurrent	AC	AC	0.00	1.00E+00	1.00E+00	1.00E+00	0
1298	84	recurrent	AC	Astrocyte	0.00	1.00E+00	3.40E-02	3.40E-02	0
1299	84	recurrent	AC	Endothelial	0.00	1.00E+00	8.52E-01	8.52E-01	0
1300	84	recurrent	AC	MES	5.00	9.99E-03	9.99E-01	9.99E-03	1
1301	84	recurrent	AC	Macrophage	0.00	1.00E+00	8.24E-01	8.24E-01	0
1302	84	recurrent	AC	Microglia	0.00	1.00E+00	8.91E-01	8.91E-01	0
1303	84	recurrent	AC	NK cell	0.00	1.00E+00	8.14E-01	8.14E-01	0
1304	84	recurrent	AC	NPC					
1305	84	recurrent	AC	Neuron	0.00	1.00E+00	9.48E-01	9.48E-01	0
1306	84	recurrent	AC	OPC					
1307	84	recurrent	AC	Oligodendrocyte	0.00	1.00E+00	6.00E-01	6.00E-01	0
1308	84	recurrent	AC	T cell	1.00	6.99E-02	9.97E-01	6.99E-02	0
1309	84	recurrent	Astrocyte	AC	0.00	1.00E+00	3.40E-02	3.40E-02	0
1310	84	recurrent	Astrocyte	Astrocyte	4.05	9.99E-04	1.00E+00	9.99E-04	1
1311	84	recurrent	Astrocyte	Endothelial	1.26	9.99E-04	1.00E+00	9.99E-04	1
1312	84	recurrent	Astrocyte	MES	1.99	9.99E-04	1.00E+00	9.99E-04	1
1313	84	recurrent	Astrocyte	Macrophage	1.12	2.00E-03	9.99E-01	2.00E-03	1
1314	84	recurrent	Astrocyte	Microglia	1.08	2.80E-02	9.73E-01	2.80E-02	0
1315	84	recurrent	Astrocyte	NK cell	1.13	6.99E-03	9.94E-01	6.99E-03	1
1316	84	recurrent	Astrocyte	NPC					
1317	84	recurrent	Astrocyte	Neuron	1.12	9.99E-04	1.00E+00	9.99E-04	1
1318	84	recurrent	Astrocyte	OPC					
1319	84	recurrent	Astrocyte	Oligodendrocyte	1.54	9.99E-04	1.00E+00	9.99E-04	1

row	patient	surgery	from cell type	to cell tye	observed count	permutations (greater than)	permutations (less than)	p value	interaction significance/direction
1320	84	recurrent	Astrocyte	T cell	1.00	1.00E+00	9.99E-04	9.99E-04	-1
1321	84	recurrent	Endothelial	AC	0.00	1.00E+00	8.52E-01	8.52E-01	0
1322	84	recurrent	Endothelial	Astrocyte	2.53	1.00E+00	9.99E-04	9.99E-04	-1
1323	84	recurrent	Endothelial	Endothelial	2.32	9.99E-04	1.00E+00	9.99E-04	1
1324	84	recurrent	Endothelial	MES	1.93	5.01E-01	5.01E-01	5.01E-01	0
1325	84	recurrent	Endothelial	Macrophage	1.35	9.99E-04	1.00E+00	9.99E-04	1
1326	84	recurrent	Endothelial	Microglia	1.15	6.49E-02	9.37E-01	6.49E-02	0
1327	84	recurrent	Endothelial	NK cell	1.37	9.99E-04	1.00E+00	9.99E-04	1
1328	84	recurrent	Endothelial	NPC					
1329	84	recurrent	Endothelial	Neuron	1.00	1.00E+00	8.28E-01	8.28E-01	0
1330	84	recurrent	Endothelial	OPC					
1331	84	recurrent	Endothelial	Oligodendrocyte	1.38	3.20E-02	9.69E-01	3.20E-02	0
1332	84	recurrent	Endothelial	T cell	1.13	6.89E-02	9.52E-01	6.89E-02	0
1333	84	recurrent	MES	AC	1.00	8.32E-01	1.00E+00	8.32E-01	0
1334	84	recurrent	MES	Astrocyte	2.70	1.00E+00	9.99E-04	9.99E-04	-1
1335	84	recurrent	MES	Endothelial	1.30	9.99E-04	1.00E+00	9.99E-04	1
1336	84	recurrent	MES	MES	2.91	9.99E-04	1.00E+00	9.99E-04	1
1337	84	recurrent	MES	Macrophage	1.22	9.99E-04	1.00E+00	9.99E-04	1
1338	84	recurrent	MES	Microglia	1.11	2.00E-03	9.99E-01	2.00E-03	1
1339	84	recurrent	MES	NK cell	1.26	9.99E-04	1.00E+00	9.99E-04	1
1340	84	recurrent	MES	NPC					
1341	84	recurrent	MES	Neuron	1.10	2.00E-03	9.99E-01	2.00E-03	1
1342	84	recurrent	MES	OPC					
1343	84	recurrent	MES	Oligodendrocyte	1.70	9.99E-04	1.00E+00	9.99E-04	1
1344	84	recurrent	MES	T cell	1.18	9.99E-04	1.00E+00	9.99E-04	1
1345	84	recurrent	Macrophage	AC	0.00	1.00E+00	8.24E-01	8.24E-01	0
1346	84	recurrent	Macrophage	Astrocyte	3.45	9.99E-04	1.00E+00	9.99E-04	1
1347	84	recurrent	Macrophage	Endothelial	1.53	9.99E-04	1.00E+00	9.99E-04	1
1348	84	recurrent	Macrophage	MES	2.35	9.99E-04	1.00E+00	9.99E-04	1
1349	84	recurrent	Macrophage	Macrophage	1.45	9.99E-04	1.00E+00	9.99E-04	1
1350	84	recurrent	Macrophage	Microglia	1.15	5.39E-02	9.53E-01	5.39E-02	0
1351	84	recurrent	Macrophage	NK cell	1.29	3.00E-03	9.98E-01	3.00E-03	1
1352	84	recurrent	Macrophage	NPC					
1353	84	recurrent	Macrophage	Neuron	1.00	1.00E+00	7.94E-01	7.94E-01	0
1354	84	recurrent	Macrophage	OPC					
1355	84	recurrent	Macrophage	Oligodendrocyte	1.36	7.39E-02	9.29E-01	7.39E-02	0
1356	84	recurrent	Macrophage	T cell	1.33	9.99E-04	1.00E+00	9.99E-04	1
1357	84	recurrent	Microglia	AC	0.00	1.00E+00	8.91E-01	8.91E-01	0
1358	84	recurrent	Microglia	Astrocyte	3.18	7.99E-03	9.93E-01	7.99E-03	1
1359	84	recurrent	Microglia	Endothelial	1.50	9.99E-04	1.00E+00	9.99E-04	1
1360	84	recurrent	Microglia	MES	2.38	9.99E-04	1.00E+00	9.99E-04	1
1361	84	recurrent	Microglia	Macrophage	1.12	2.40E-01	7.61E-01	2.40E-01	0
1362	84	recurrent	Microglia	Microglia	1.35	8.99E-03	9.92E-01	8.99E-03	1
1363	84	recurrent	Microglia	NK cell	1.38	2.00E-03	9.99E-01	2.00E-03	1
1364	84	recurrent	Microglia	NPC					
1365	84	recurrent	Microglia	Neuron	1.00	9.99E-01	8.79E-01	8.79E-01	0
1366	84	recurrent	Microglia	OPC					
1367	84	recurrent	Microglia	Oligodendrocyte	1.25	5.75E-01	4.58E-01	4.58E-01	0
1368	84	recurrent	Microglia	T cell	1.31	6.99E-03	9.94E-01	6.99E-03	1
1369	84	recurrent	NK cell	AC	0.00	1.00E+00	8.14E-01	8.14E-01	0
1370	84	recurrent	NK cell	Astrocyte	3.16	2.00E-03	9.99E-01	2.00E-03	1
1371	84	recurrent	NK cell	Endothelial	1.76	9.99E-04	1.00E+00	9.99E-04	1
1372	84	recurrent	NK cell	MES	2.39	9.99E-04	1.00E+00	9.99E-04	1
1373	84	recurrent	NK cell	Macrophage	1.31	2.00E-03	9.99E-01	2.00E-03	1
1374	84	recurrent	NK cell	Microglia	1.31	9.99E-04	1.00E+00	9.99E-04	1
1375	84	recurrent	NK cell	NK cell	1.68	9.99E-04	1.00E+00	9.99E-04	1
1376	84	recurrent	NK cell	NPC					
1377	84	recurrent	NK cell	Neuron	1.11	7.69E-02	9.46E-01	7.69E-02	0
1378	84	recurrent	NK cell	OPC					
1379	84	recurrent	NK cell	Oligodendrocyte	1.59	9.99E-04	1.00E+00	9.99E-04	1
1380	84	recurrent	NK cell	T cell	1.57	9.99E-04	1.00E+00	9.99E-04	1
1381	84	recurrent	NPC	AC					
1382	84	recurrent	NPC	Astrocyte					
1383	84	recurrent	NPC	Endothelial					
1384	84	recurrent	NPC	MES					
1385	84	recurrent	NPC	Macrophage					
1386	84	recurrent	NPC	Microglia					
1387	84	recurrent	NPC	NK cell					
1388	84	recurrent	NPC	NPC					
1389	84	recurrent	NPC	Neuron					
1390	84	recurrent	NPC	OPC					
1391	84	recurrent	NPC	Oligodendrocyte					
1392	84	recurrent	NPC	T cell					
1393	84	recurrent	Neuron	AC	0.00	1.00E+00	9.48E-01	9.48E-01	0
1394	84	recurrent	Neuron	Astrocyte	3.51	9.99E-04	1.00E+00	9.99E-04	1
1395	84	recurrent	Neuron	Endothelial	1.50	5.00E-03	9.99E-01	5.00E-03	1
1396	84	recurrent	Neuron	MES	2.94	9.99E-04	1.00E+00	9.99E-04	1
1397	84	recurrent	Neuron	Macrophage	1.10	4.01E-01	6.45E-01	4.01E-01	0
1398	84	recurrent	Neuron	Microglia	1.00	9.99E-01	7.28E-01	7.28E-01	0
1399	84	recurrent	Neuron	NK cell	1.25	9.49E-02	9.40E-01	9.49E-02	0
1400	84	recurrent	Neuron	NPC					
1401	84	recurrent	Neuron	Neuron	1.17	2.70E-02	9.74E-01	2.70E-02	0
1402	84	recurrent	Neuron	OPC					
1403	84	recurrent	Neuron	Oligodendrocyte	1.56	1.20E-02	9.90E-01	1.20E-02	0
1404	84	recurrent	Neuron	T cell	1.25	7.49E-02	9.50E-01	7.49E-02	0
1405	84	recurrent	OPC	AC					
1406	84	recurrent	OPC	Astrocyte					
1407	84	recurrent	OPC	Endothelial					
1408	84	recurrent	OPC	MES					
1409	84	recurrent	OPC	Macrophage					
1410	84	recurrent	OPC	Microglia					
1411	84	recurrent	OPC	NK cell					
1412	84	recurrent	OPC	NPC					
1413	84	recurrent	OPC	Neuron					
1414	84	recurrent	OPC	OPC					
1415	84	recurrent	OPC	Oligodendrocyte					
1416	84	recurrent	OPC	T cell					
1417	84	recurrent	Oligodendrocyte	AC	0.00	1.00E+00	6.00E-01	6.00E-01	0
1418	84	recurrent	Oligodendrocyte	Astrocyte	2.44	1.00E+00	9.99E-04	9.99E-04	-1
1419	84	recurrent	Oligodendrocyte	Endothelial	1.30	9.99E-04	1.00E+00	9.99E-04	1
1420	84	recurrent	Oligodendrocyte	MES	2.14	9.99E-04	1.00E+00	9.99E-04	1
1421	84	recurrent	Oligodendrocyte	Macrophage	1.06	7.58E-01	2.56E-01	2.56E-01	0
1422	84	recurrent	Oligodendrocyte	Microglia	1.14	9.99E-03	9.92E-01	9.99E-03	1
1423	84	recurrent	Oligodendrocyte	NK cell	1.20	9.99E-04	1.00E+00	9.99E-04	1
1424	84	recurrent	Oligodendrocyte	NPC					
1425	84	recurrent	Oligodendrocyte	Neuron	1.00	1.00E+00	5.58E-01	5.58E-01	0
1426	84	recurrent	Oligodendrocyte	OPC					
1427	84	recurrent	Oligodendrocyte	Oligodendrocyte	2.78	9.99E-04	1.00E+00	9.99E-04	1
1428	84	recurrent	Oligodendrocyte	T cell	1.42	9.99E-04	1.00E+00	9.99E-04	1
1429	84	recurrent	T cell	AC	1.00	6.99E-02	1.00E+00	6.99E-02	0
1430	84	recurrent	T cell	Astrocyte	2.93	3.85E-01	6.16E-01	3.85E-01	0

row	patient	surgery	from cell type	to cell tye	observed count	permutations (greater than)	permutations (less than)	p value	interaction significance/direction
1431	84	recurrent	T cell	Endothelial	1.80	9.99E-04	1.00E+00	9.99E-04	1
1432	84	recurrent	T cell	MES	2.50	9.99E-04	1.00E+00	9.99E-04	1
1433	84	recurrent	T cell	Macrophage	1.12	2.44E-01	7.59E-01	2.44E-01	0
1434	84	recurrent	T cell	Microglia	1.35	6.99E-03	9.94E-01	6.99E-03	1
1435	84	recurrent	T cell	NK cell	1.42	3.00E-03	9.98E-01	3.00E-03	1
1436	84	recurrent	T cell	NPC					
1437	84	recurrent	T cell	Neuron	1.00	9.88E-01	9.14E-01	9.14E-01	0
1438	84	recurrent	T cell	OPC					
1439	84	recurrent	T cell	Oligodendrocyte	1.56	3.00E-03	9.98E-01	3.00E-03	1
1440	84	recurrent	T cell	T cell	1.59	3.00E-03	9.98E-01	3.00E-03	1

Supplementary Table 11. Summarised pair-wise cell-cell interactions compared to a null model of spatial randomness. Statistical significance and direction of the interactions were determined using a permutation test, with p-values indicating interactions more or less likely than random. The interaction significance/direction denotes the strength and direction of all the pair-wise interactions: >=1 (significant attraction interactions); <= -1 (significant avoidance interactions); 0 (neutral and/or non-statistically significant interactions). When an interaction type is significant across both primary and recurrent surgeries, both surgery-specific p values are listed.

row	from cell	to cell	significant interaction type	present across (surgery)	interaction significance	primary p value	recurrent p value
1	AC	AC	Interacting	Primary	3	9.99E-04	
2	AC	Astrocyte			0		
3	AC	Endothelial			2		
4	AC	MES			-1		
5	AC	Macrophage			1		
6	AC	Microglia			0		
7	AC	NK cell			0		
8	AC	NPC			1		
9	AC	Neuron			0		
10	AC	OPC			2		
11	AC	Oligodendrocyte			1		
12	AC	T cell			1		
13	Astrocyte	AC			2		
14	Astrocyte	Astrocyte	Interacting	Both	3	9.99E-04	9.99E-04
15	Astrocyte	Endothelial	Interacting	Recurrent	2		9.99E-04
16	Astrocyte	MES	Interacting	Recurrent	2		9.99E-04
17	Astrocyte	Macrophage	Interacting	Recurrent	1		2.00E-03
18	Astrocyte	Microglia			2		
19	Astrocyte	NK cell	Interacting	Recurrent	0		9.99E-04
20	Astrocyte	NPC	Interacting	Recurrent	1		9.99E-04
21	Astrocyte	Neuron			2		
22	Astrocyte	OPC	Interacting	Recurrent	2		9.99E-04
23	Astrocyte	Oligodendrocyte	Interacting	Recurrent	0		9.99E-04
24	Astrocyte	T cell			1		
25	Endothelial	AC			2		
26	Endothelial	Astrocyte	Interacting	Primary	3	9.99E-04	
27	Endothelial	Endothelial	Interacting	Both	5	9.99E-04	9.99E-04
28	Endothelial	MES	Interacting	Primary	4	9.99E-04	
29	Endothelial	Macrophage	Interacting	Recurrent	0		9.99E-04
30	Endothelial	Microglia	Interacting	Primary	3	9.99E-04	
31	Endothelial	NK cell	Interacting	Both	4	9.99E-04	9.99E-04
32	Endothelial	NPC			0		
33	Endothelial	Neuron			1		
34	Endothelial	OPC			2		
35	Endothelial	Oligodendrocyte			1		
36	Endothelial	T cell			2		
37	MES	AC			-1		
38	MES	Astrocyte			2		
39	MES	Endothelial	Interacting	Both	3	9.99E-04	9.99E-04
40	MES	MES	Interacting	Both	5	9.99E-04	9.99E-04
41	MES	Macrophage	Interacting	Recurrent	1		9.99E-04
42	MES	Microglia			2		
43	MES	NK cell	Interacting	Both	3	9.99E-04	9.99E-04
44	MES	NPC			-2		
45	MES	Neuron			0		
46	MES	OPC			-2		
47	MES	Oligodendrocyte	Interacting	Recurrent	1		9.99E-04
48	MES	T cell	Interacting	Primary	4	9.99E-04	
49	Macrophage	AC	Interacting	Primary	3	9.99E-04	
50	Macrophage	Astrocyte	Interacting	Primary	4	9.99E-04	
51	Macrophage	Endothelial	Interacting	Both	5	9.99E-04	9.99E-04
52	Macrophage	MES	Interacting	Both	3	9.99E-04	9.99E-04
53	Macrophage	Macrophage	Interacting	Both	3	9.99E-04	9.99E-04
54	Macrophage	Microglia	Interacting	Both	5	9.99E-04	9.99E-04
55	Macrophage	NK cell	Interacting	Both	5	9.99E-04	9.99E-04
56	Macrophage	NPC			2		
57	Macrophage	Neuron	Interacting	Primary	3	9.99E-04	
58	Macrophage	OPC			1		
59	Macrophage	Oligodendrocyte	Interacting	Recurrent	2		9.99E-04
60	Macrophage	T cell	Interacting	Recurrent	2		9.99E-04
61	Microglia	AC			2		
62	Microglia	Astrocyte	Interacting	Primary	4	9.99E-04	
63	Microglia	Endothelial	Interacting	Both	5	9.99E-04	9.99E-04
64	Microglia	MES	Interacting	Both	4	9.99E-04	9.99E-04
65	Microglia	Macrophage	Interacting	Both	3	9.99E-04	9.99E-04
66	Microglia	Microglia	Interacting	Both	4	9.99E-04	9.99E-04
67	Microglia	NK cell	Interacting	Recurrent	2		9.99E-04
68	Microglia	NPC	Interacting	Recurrent	1		9.99E-04
69	Microglia	Neuron			0		
70	Microglia	OPC			2		
71	Microglia	Oligodendrocyte	Interacting	Recurrent	2		9.99E-04
72	Microglia	T cell	Interacting	Both	4	9.99E-04	9.99E-04

row	from cell	to cell	significant interaction type	present across (surgery)	interaction significance	primary p value	recurrent p value
73	NK cell	AC			2		
74	NK cell	Astrocyte			0		
75	NK cell	Endothelial	Interacting	Both	5	9.99E-04	9.99E-04
76	NK cell	MES			1		
77	NK cell	Macrophage	Interacting	Recurrent	2		9.99E-04
78	NK cell	Microglia	Interacting	Both	4	9.99E-04	9.99E-04
79	NK cell	NK cell	Interacting	Both	3	9.99E-04	9.99E-04
80	NK cell	NPC			2		
81	NK cell	Neuron			1		
82	NK cell	OPC			2		
83	NK cell	Oligodendrocyte	Interacting	Recurrent	0		9.99E-04
84	NK cell	T cell	Interacting	Both	3	9.99E-04	9.99E-04
85	NPC	AC			1		
86	NPC	Astrocyte	Interacting	Recurrent	1		9.99E-04
87	NPC	Endothelial			2		
88	NPC	MES			0		
89	NPC	Macrophage			1		
90	NPC	Microglia			1		
91	NPC	NK cell			-1		
92	NPC	NPC	Interacting	Recurrent	1		9.99E-04
93	NPC	Neuron			2		
94	NPC	OPC			2		
95	NPC	Oligodendrocyte			1		
96	NPC	T cell			0		
97	Neuron	AC			2		
98	Neuron	Astrocyte	Interacting	Recurrent	2	9.99E-04	9.99E-04
99	Neuron	Endothelial	Interacting	Recurrent	1	9.99E-04	9.99E-04
100	Neuron	MES	Interacting	Recurrent	2	9.99E-04	9.99E-04
101	Neuron	Macrophage			2		
102	Neuron	Microglia			1		
103	Neuron	NK cell			-1		
104	Neuron	NPC			2		
105	Neuron	Neuron			2		
106	Neuron	OPC			2		
107	Neuron	Oligodendrocyte			1		
108	Neuron	T cell			1		
109	OPC	AC			1		
110	OPC	Astrocyte			1		
111	OPC	Endothelial			1		
112	OPC	MES			1		
113	OPC	Macrophage			1		
114	OPC	Microglia			1		
115	OPC	NK cell			-1		
116	OPC	NPC			1		
117	OPC	Neuron			2		
118	OPC	OPC			2		
119	OPC	Oligodendrocyte			1		
120	OPC	T cell			1		
121	Oligodendrocyte	AC			1		
122	Oligodendrocyte	Astrocyte			1		
123	Oligodendrocyte	Endothelial	Interacting	Recurrent	2		9.99E-04
124	Oligodendrocyte	MES	Interacting	Primary	3	9.99E-04	
125	Oligodendrocyte	Macrophage			2		
126	Oligodendrocyte	Microglia	Interacting	Recurrent	2		9.99E-04
127	Oligodendrocyte	NK cell			-1		
128	Oligodendrocyte	NPC			1		
129	Oligodendrocyte	Neuron			1		
130	Oligodendrocyte	OPC			1		
131	Oligodendrocyte	Oligodendrocyte	Interacting	Recurrent	2		9.99E-04
132	Oligodendrocyte	T cell	Interacting	Recurrent	2		9.99E-04
133	T cell	AC			2		
134	T cell	Astrocyte			1		
135	T cell	Endothelial	Interacting	Both	5	9.99E-04	9.99E-04
136	T cell	MES	Interacting	Both	5	9.99E-04	9.99E-04
137	T cell	Macrophage			0		
138	T cell	Microglia	Interacting	Both	4	9.99E-04	9.99E-04
139	T cell	NK cell	Interacting	Both	3	9.99E-04	9.99E-04
140	T cell	NPC			1		
141	T cell	Neuron			1		
142	T cell	OPC			2		
143	T cell	Oligodendrocyte	Interacting	Recurrent	2		9.99E-04
144	T cell	T cell	Interacting	Both	3	9.99E-04	9.99E-04

Supplementary Table 12. Summary of defined cellular neighborhoods (CNs). The table details how each CN aligns with Greenwald et al.'s metaprograms, and their relative proportions in primary and recurrent samples.

cellular neighbourhood	Greenwald et.al layer	Greenwald et.al label	n cells (primary)	proportion (primary)	n cells (recurrent)	proportion (recurrent)
CN1	3	Mac	6943	12.4%	26	0.1%
CN2	2	MES-Ast	2938	5.2%	6143	16.1%
CN3	5	Oligo	3454	6.2%	3009	7.9%
CN4	3	T-cell	6721	12.0%	4418	11.6%
CN5	3	Vasc	5171	9.2%	1560	4.1%
CN6	1	MES-Hyp	9186	16.4%	2451	6.4%
CN7	5	Neuron	1469	2.6%	5737	15.1%
CN8	4	AC	7961	14.2%	887	2.3%
CN9	5	Reactive-Ast/Neuron	765	1.4%	4931	12.9%
CN10	3	T-cell	3454	6.2%	543	1.4%
CN11	5	Reactive-Ast	2725	4.9%	7641	20.1%
CN12	2	Inflammatory-Mac	5179	9.3%	746	2.0%

Supplementary Table 13. Comparison of cellular state (hypoxia and Epithelial to mesenchymal transition - EMT) protein marker abundance between primary and recurrent surgery samples and across each defined cellular neighbourhood (CN). Statistical significance was assessed using an unpaired Wilcoxon test, with adjusted p-values calculated using the false discovery rate (FDR) method. The p value significance levels are denoted using the following symbols: **** ($p < 0.0001$); *** ($p < 0.001$); ** ($p < 0.01$); * ($p < 0.05$); n.s (not significant).

Greenwald_et_al_label (CN)	cellular state	comparison groups	n cells (primary)	n cells (recurrent)	p value	adjusted p value	p significance
MES-Hyp (CN6)	hypoxia	Prim vs Rec	6527	1407	3.70E-20	6.78E-20	****
MES-Ast (CN2)	hypoxia	Prim vs Rec	748	811	1.52E-74	1.67E-73	****
Inflammatory-Mac (CN12)	hypoxia	Prim vs Rec	777	71	8.52E-01	9.13E-01	n.s
Mac (CN1)	hypoxia	Prim vs Rec	262	2	6.77E-02	8.27E-02	n.s
Vasc (CN5)	hypoxia	Prim vs Rec	554	36	8.02E-03	1.10E-02	**
T-cell (CN4 & CN10)	hypoxia	Prim vs Rec	2406	621	1.40E-30	3.08E-30	****
AC (CN8)	hypoxia	Prim vs Rec	5258	633	6.80E-66	3.74E-65	****
Neuron (CN7)	hypoxia	Prim vs Rec	311	310	9.13E-01	9.13E-01	n.s
Reactive-Ast/Neuron (CN9)	hypoxia	Prim vs Rec	106	225	1.07E-09	1.68E-09	****
Reactive-Ast (CN11)	hypoxia	Prim vs Rec	248	478	1.36E-41	3.74E-41	****
Oligo (CN3)	hypoxia	Prim vs Rec	873	440	4.17E-42	1.53E-41	****
MES-Hyp (CN6)	EMT	Prim vs Rec	6527	1407	6.11E-01	6.12E-01	n.s
MES-Ast (CN2)	EMT	Prim vs Rec	748	811	2.69E-27	9.86E-27	****
Inflammatory-Mac (CN12)	EMT	Prim vs Rec	777	71	1.43E-04	2.25E-04	***
Mac (CN1)	EMT	Prim vs Rec	262	2	6.12E-01	6.12E-01	n.s
Vasc (CN5)	EMT	Prim vs Rec	554	36	4.34E-07	7.96E-07	****
T-cell (CN4 & CN10)	EMT	Prim vs Rec	2406	621	2.82E-59	1.55E-58	****
AC (CN8)	EMT	Prim vs Rec	5258	633	1.04E-195	1.14E-194	****
Neuron (CN7)	EMT	Prim vs Rec	311	310	1.14E-01	1.39E-01	n.s
Reactive-Ast/Neuron (CN9)	EMT	Prim vs Rec	106	225	6.90E-04	9.49E-04	***
Reactive-Ast (CN11)	EMT	Prim vs Rec	248	478	1.80E-11	3.96E-11	****
Oligo (CN3)	EMT	Prim vs Rec	873	440	3.60E-22	9.90E-22	****

Supplemental Methods

Imaging Mass Cytometry (IMC) Analysis

Data acquisition and initial processing of samples was as previously detailed [15].

Briefly, we analysed paired GBM patient samples collected at two distinct timepoints: during the initial de-bulking surgery (primary samples) and at the time of first recurrence (Supplementary Table 1 and Supplementary Figure 1). For each patient/surgery tissue sample we sampled three spatially distinct 1mm^2 regions of interest (ROIs) across 5 patients (30 total regions) (Supplementary Table 2 and Supplementary Figure 2). An antibody panel of 34 proteins was designed and control tissues were used to validate each antibody (Supplementary Table 3). Following this 5µm tissue sections were stained with a cocktail of all conjugated antibodies and each ROI was ablated at a 200Hz frequency with a 1-micron diameter laser. The raw MCD files were exported as exported in OME-TIFF format.

All IMC image processing and downstream analysis steps were performed using the R statistical software package (>= version 4.3.0) and Python (version 3.11.3). Cell-cell interactions, neighbourhoods and contexts were generated using functions from imcRtools (v1.10.0). All plots were generated using ggplot2 (version 3.5.1).

Cell Segmentation

Steinbock [16] (v0.13.5) converted raw MCD files into multi-channel TIFF images (n=30), which were processed with tifffile (v2023.4.12). Nuclear (Ir191, Ir193) and cytoplasmic (Sm149, Eu153, Dy164, Yb171) channels were combined into single RGB images using pandas (v2.0.3) and numpy (v1.24.0), followed by random cropping into 100µm2 sections. Cellpose (v2.0) was used for segmentation as previously described [17]. Random RGB crops were segmented with the pre-trained cytoplasm model (“cyto2”), iteratively re-annotated, and refined by updating the model and adjusting the mean pixel diameter. The final model was applied to full 1000µm2 ROIs using the “cellpose_train” command

with default settings. Signal intensities were extracted using the “measure intensities” function in Steinbock: pixels were aggregated by computing the mean intensity across channels. Additional spatial features were calculated using the “measure regionprops” function (Supplementary Figure 3).

Single-Cell & Image Processing

The quantified single cell data and images were processed as previously detailed [15]. Briefly, segmented cell expression counts were transformed using an inverse hyperbolic sine (asinh) function (cofactor = 5). Following this, single-cells were integrated to correct for unwanted sources of variation present across each patient using harmony (Supplementary Figures 4-5).

Cell Phenotyping

Cells with high expression (>90th percentile) across multiple markers (>50% of marker) were excluded from phenotyping. Expression counts were z-score normalised, was ranked from low (1) to high (20) based on expression levels. Labels were assigned using a logical gating approach detailed in Supplementary Table 4: labels were only assigned if the marker expression rank exceeded a user-defined threshold. Related cell types such as stromal and malignant cells shared markers which differed only in their expression levels making them difficult to label. To account for this we applied marker-specific thresholds across multiple marker and generated final cell-type labels by combining cells falling within the intersecting marker expression criteria. Cells were classified as having low (< -1.2) or high (> 1.2) hypoxia (HIF1A+) and epithelial-to-mesenchymal transition (EMT) (SNAI1+) based on their z-score normalised expression counts.

Measurement of Intra-patient heterogeneity

Shannon entropy (H) was used to measure the compositional diversity of cell types across ROIs. To account for differences in cell frequencies across ROIs, 1,000 cells were randomly sub-sampled per group (i), in each of ten iterations. In each round, Shannon entropy was calculated using cell type frequencies (P_c) as:

$$H_i = -\sum_{c} P_c \log_2(P_c) \quad (1)$$

The Wilcoxon rank-sum test was used to compare Shannon entropy (H) between primary and recurrent surgeries, both across all data and within each patient (n = 5).

Spatial Interaction Graphs

Spatial interaction graphs were generated for each ROI (n = 30) using the buildSpatialGraph function from imcRtools with the Delaunay triangulation method. To reduce spurious connections, edges were pruned by setting the max_dist argument to 50µm.

Testing Cell-Cell Interactions

Cell-cell spatial interactions were tested using the testInteractions from imcRtools with the previously described "histoCAT" method [18]. Briefly, spatial interaction graph edges were summed and aggregated across individual patient/surgery ROIs and then divided by the number of cells of type A that had at least one neighbour of type B. These observed interactions were compared against a derived null distribution, describing the interactions formed under spatial randomness. Significance was determined using two one-tailed permutation tests:

$$P_{AB} = \begin{cases} 1, & C_{obs} = 0; \\ \dfrac{\sum\left(C_{perm} \geq (\leq) C_{obs}\right) + 1}{N_{perm} + 1}, & otherwise, \end{cases} \quad (2)$$

where C_{perm} is the number of cell pairs (A, B) in each permutation, C_{obs} is the actual number of cell pairs (A, B) given a defined distance, and N_{perm} is the number of permutations.

Cell Neighbourhoods (CNs) and Spatial Contexts (SCs)

Cellular neighbourhoods (CNs) and Spatial Contexts (SCs) were defined using a previously established method[19]. Briefly, for each cell in each ROI (n = 30) , the proportion of neighbouring cell types was aggregated using the aggregateNeighbors function from imcRtools. These proportions were then clustered using k-means with k = 12, determined through a parameter sweep and visual inspection.

CNs were further aggregated and SCs identified using the detectSpatialContext function from imcRtools with default parameters. The filterSpatialContext function from imcRtools was used to further refine SCs, retaining the most dominant: 1.) SCs present in >3 patients; and 2.) SCs comprising >5% of total cells within each surgery type.

Spatial Transcriptomics Tissue Preparation

Formalin-fixed paraffin-embedded (FFPE) glioblastoma (GBM) tissue cores, embedded in a tissue microarray (TMA), were profiled using the NanoString CosMX™ Spatial Molecular Imager (SMI) with the Human Discovery 6k RNA Panel. Tissue sections were stained using multiplexed immunofluorescence (IF) markers, including DAPI, histone, rRNA, and GFAP. Probes targeting 6,195 RNA transcripts were washed over the TMA cores and hybridised in situ, followed by imaging at subcellular resolution. Field-of-view (FOV) placement across the TMA cores was guided by haematoxylin and eosin (H&E) reference images and quality control assessments.

Spatial Transcriptomics Cell Phenotyping

Cell segmentation was performed using a machine learning-based, multimodal approach utilising the IF stain markers. Transcript counts were assigned to individual cells using spatial coordinates. Cells located in regions with technical issues, like autofluorescence, were excluded. Furthermore, cells with ambiguous, or low-confidence segmentation because of poor IF signal were also removed.

Probes signal was corrected for by subtracting background signal, using negative probes, before the counts were scaled per cell, to normalise for sequencing. Cells with abnormally low total transcript and gene counts were excluded. Low-variance genes were filtered out before performing dimensionality reduction, using Uniform Manifold Approximation and Projection (UMAP), and cell clustering, using both unsupervised Leiden clustering and semi-supervised InSituType classification, based on a curated panel of 850 genes relevant to the GBM tumour microenvironment. Marker gene expression profiles were then used to annotate clusters and identify key cell types, including cancer cells, immune cells, and neuronal cells. Final cell type annotations were supported by marker gene heatmaps and spatial mapping of cell distributions across samples.

Mapping Spatial Transcripomic Cell Types to Greenwald MPs

The cell types identified using spatial transcripomics data were mapped to the metaprograms (MPs) defined by Greenwald et al. (2024)[11], by taking the top 20 marker genes per cluster identified through differential expression analysis. Enrichment analysis was then performed between these markers and the MP gene sets from Greenwald et al. (2024)[11], with each cluster assigned to the MP showing the highest degree of overlap.

In parallel, average gene expression was computed per cluster, scaled, and converted into z-scores across all clusters. These were integrated with a separately filtered set of marker genes (adjusted $p < 0.01$) for further MP alignment. This two-step enrichment approach yielded consistent and biologically interpretable matches between the NanoString-derived clusters and Greenwald MPs.
